# Gabapentin Utilization and Adverse Effects Among US Hemodialysis Patients Diagnosed With Pruritus or Neuropathic Pain

**DOI:** 10.1016/j.xkme.2026.101341

**Published:** 2026-03-18

**Authors:** Claudio Rigatto, Ting Lu, Thilo Schaufler, Despina Ruessmann, Ronald L. Pisoni, Brian Bieber, Chad Cogan, Angelo Karaboyas, Michael Strupp

**Affiliations:** 1 Chronic Disease Innovation Centre, Seven Oaks General Hospital, Winnipeg, Manitoba, Canada; 2 Department of Internal Medicine, Section of Nephrology, Max Rady Faculty of Health Sciences, University of Manitoba, Winnipeg, Manitoba, Canada; 3 Arbor Research Collaborative for Health, Ann Arbor, MI; 4 CSL Vifor, Glattbrugg, Switzerland; 5 Department of Neurology, LMU Hospital, LMU Munich, Germany

**Keywords:** pruritus, gabapentin, hemodialysis, USRDS, adverse events

## Abstract

**Rationale & Objective:**

Gabapentinoids, including gabapentin and pregabalin, are indicated for patients with neuropathic pain and are also prescribed off-label to treat chronic kidney disease-associated pruritus in hemodialysis (HD) patients. With limited data on effectiveness in pruritus and concerns about adverse effects, the risk-benefit profile is controversial.

**Study Design:**

A retrospective analysis of registry data.

**Setting & Participants:**

A total of 533,232 HD patients from the United States Renal Data System from 2016-2020

**Exposures:**

Gabapentinoid use/dose, updated over time based on prescription changes.

**Outcomes:**

Five adverse effects based on ICD-10 codes: altered mental state, dizziness, fracture, falls, somnolence.

**Analytical Approach:**

We investigated gabapentinoid utilization patterns and associations between dose and adverse effects using Cox-based recurrent event Anderson-Gill models. Analyses were adjusted for potential confounders and stratified by time-updated diagnosis groups (NP vs pruritus).

**Results:**

Among patients with neuropathic pain, 19% used gabapentin with a mean dose 503 mg/day. Among patients with pruritus, 11% used gabapentin with mean dose 433 mg/day. Event rates (per 100 patient-years) for adverse effects were 37.8 for altered mental state, 20.4 for falls, 19.3 for dizziness, 15.2 for somnolence, and 9.3 for fracture. Adjusted models showed a clear dose-response association (*P* < 0.001) between gabapentin dose and all adverse effects—especially among patients with pruritus—with elevated risk observed at doses as low as 100 mg/day. Pregabalin use was only 2%, with a dose-response adverse effect profile similar to gabapentin.

**Limitations:**

Under-ascertainment of pruritus via diagnosis codes; residual confounding.

**Conclusions:**

Gabapentinoid utilization patterns differed by diagnosis, with use and doses higher among those with neuropathic pain versus pruritus. Adverse effects—even at low doses—were common, particularly when used off-label in patients with pruritus and no neuropathic pain. With new alternatives available, prescribers should weigh the clinically relevant adverse effects of gabapentinoids when providing treatment options to patients with pruritus.

## Plain-Language Summary

Gabapentin and pregabalin are medicines often used to treat nerve pain. They are also used to treat itching in people on dialysis, even though they are not approved for that purpose. This analysis of registry data showed that people with nerve pain were given higher doses of gabapentin than those with itching. However, even small doses of gabapentin were linked to side effects like confusion, dizziness, and falls—especially in people who were taking it for itching. These results show that doctors should be very careful when giving gabapentin for itching and may need to look for safer treatments.

Gabapentinoids, including gabapentin and pregabalin, are indicated for patients with neuropathic pain, in the general population as well as the hemodialysis (HD) setting.^1^ While effective,^2^^,^^3^ risk of adverse events—including altered mental status, dizziness, falls, and somnolence—is high.^4^^,^^5^ When also considering the risks of abuse and withdrawal,^6^^,^^7^ these medications have a controversial risk-benefit profile,^8^ particularly among patients with kidney failure and neuropathic pain for whom the label-recommended dosages are lower.^9^

Gabapentinoids have also been shown to reduce itch symptoms, though studies have generally been small and without a robust comparison group.^10^, ^11^, ^12^, ^13^ Despite limited evidence, gabapentinoids are being prescribed off-label to treat chronic kidney disease-associated pruritus,^14^ with about 10% use observed in the HD setting.^15^ Recent research from the Dialysis Outcomes and Practice Patterns Study has shown that pruritus is prevalent in 35%-40% of HD patients and associated with numerous adverse clinical and patient-reported outcomes.^16^^,^^17^ When considering the high burden of pruritus in the HD population, effectiveness and adverse events must carefully be weighed to determine the course of treatment,^18^ particularly in chronic diseases which require lifelong treatment.

Prior research demonstrated a strong dose-response association between gabapentinoids and selected adverse events in the US HD population using data from the United States Renal Data System (USRDS).^19^ In this study, we build on these results with contemporary USRDS data to better understand different aspects of gabapentinoid utilization in this setting. We investigated gabapentinoid use, dose, time to discontinuation, and associations with relevant adverse events—all stratified by prior diagnosis of neuropathic pain versus pruritus.

## Methods

### Study Design

This study is a retrospective analysis of USRDS data. Our objectives are to describe the utilization of gabapentinoids—primarily gabapentin but also pregabalin—in the US HD setting, and their associations with adverse events, focusing on subgroups of patients with a diagnosis of neuropathic pain or pruritus. This approach, isolating subsets of patients within a diagnosis group who may share underlying clinical risks, should make non-users of gabapentin more comparable to users, thereby reducing confounding.

### Data Source

The USRDS is a national data system that collects, analyzes, and distributes information about chronic kidney disease and patients with kidney failure in the United States (US), and offers access to researchers through project-specific data use agreements. For this analysis, Institutional review board exemption was received, with E&I Assigned Study ID: 23118 – 01; informed consent was not needed due to all data being de-identified and analyzed retrospectively.

**Figure 1:**
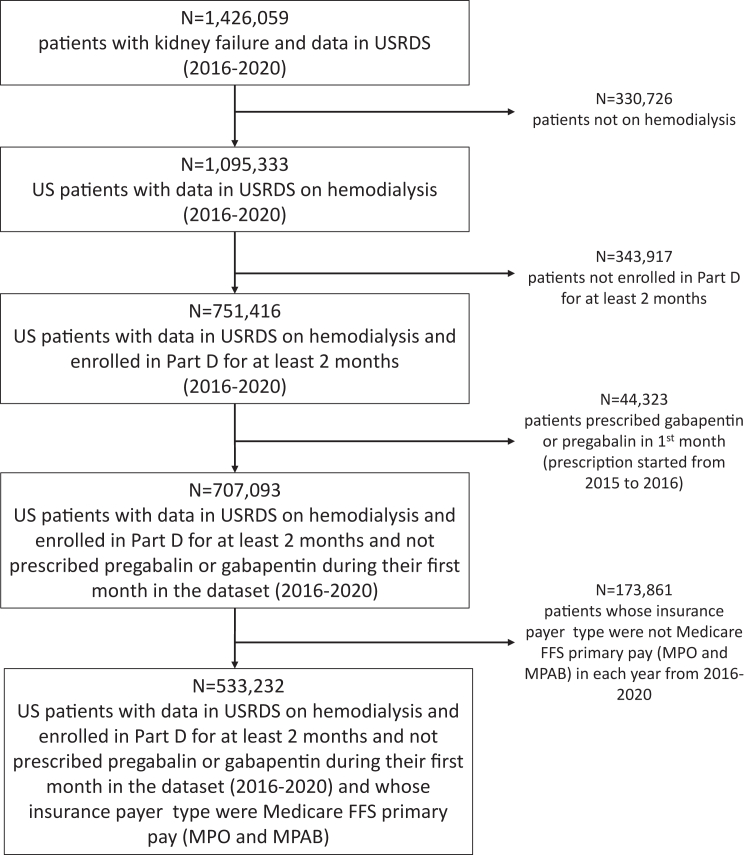
Flow chart of study inclusion/exclusion criteria.

Analyses were limited to the dialysis fee-for-service Medicare population (where outcomes can be defined via claims) aged 18 years or older and focused on the subset of beneficiaries with Medicare Part D coverage (where gabapentinoid prescriptions can be defined). The pool of eligible patients was defined as US HD patients recorded in the USRDS between 2016-2020. To qualify, patients must have been enrolled in Medicare Part D for at least 2 months, and have Medicare fee-for-service as their primary payer, categorized as either Medicare Primary Only or Medicare Primary with Additional Benefits. To implement a new user approach, patients prescribed a gabapentinoid during their first month in the dataset were excluded. The number of patients meeting inclusion/exclusion criteria is summarized in Fig 1; ultimately, 533,232 HD patients were included in the analysis.

### Variables

#### Exposure

In the primary analysis examining the association between gabapentin dose and adverse events, the exposure was defined as the time-updated gabapentin dose in milligrams per day. This is calculated for each prescription record in the Part D claims data from 2016-2020 using the following formula: Gabapentin dose (mg/day) = (Quantity dispensed ∗ Active ingredient strength in mg)/days supply. The days supply number is recorded in kidney failure Medicare prescription drug event data, which contains details on each drug prescription. Based on the observed distribution of doses, we categorized the gabapentin dose into six groups: 0, 100, 200, 300, 400-800, and 900+ mg/day. Most (83%) doses were in increments of 100; others were rounded to the nearest 100 for ease of presentation. Missing dose records were excluded; outlier dose records were also excluded, defined as <40 mg/day or >2,700 mg/day. For the secondary analysis of the association between pregabalin dose and adverse events, the exposure was similarly defined as the time-updated pregabalin dose in milligrams per day. Based on the distribution of doses and clinical criteria, we categorized the pregabalin dose into 4 groups: 0, 25-75, 100-150, and 200+ mg/day. Most (95%) doses were in increments of 25; others were rounded to the nearest 25. We defined the gabapentin or pregabalin dose for each day a patient was represented in the dataset, with doses updated over time as prescriptions changed.

#### Outcomes

We included 5 adverse effect outcomes in our analysis: altered mental state, dizziness, falls, fracture, and somnolence. These outcomes were defined using ICD-10 and ICD-9 code sets described in Appendix A in the Supplementary Material. For each patient, every event related to these adverse effects was recorded separately during the period from 2016-2020. To avoid bias from closely spaced events that may be related, a 90-day moratorium period was imposed so that only independent events were considered.

#### Stratification

For each day from 2016-2020, patients were assigned to one of the diagnosis groups based on ICD-10 codes (Appendix B): pruritus, neuropathic pain, Both pruritus and neuropathic pain, or neither. Group assignment was time-updated, meaning a previous diagnosis group could be replaced by a new diagnosis group based on the most recent diagnosis. Patients begin in the neither group and can transition to either the pruritus or neuropathic pain group, and then potentially transition to the both group during follow-up. Patients in the pruritus or neuropathic pain groups cannot revert back to the neither group, and patients in the both group cannot revert back to the pruritus or neuropathic pain group–there is no expiration date to a diagnosis. Throughout follow-up, a patient could thus belong to 1, 2, or 3 diagnosis groups over time.

#### Covariates

Demographic information (eg, age, sex, race, ethnicity) was ascertained from the Master Beneficiary Summary File (MBSF). Comorbidity history was captured from the CMS 2728 form as of the start of dialysis. Concomitant medications (e.g., opioids, benzodiazepines, antihistamines) were captured in the Medicare Part D data.

### Statistical Analysis

First, we examined patient characteristics across different diagnosis groups and gabapentin dose categories to explore variations in patient profiles within each diagnosis group and across different levels of gabapentin dose. Second, we analyzed the utilization patterns of gabapentin by calculating the point prevalence of gabapentin prescriptions on December 31st each year, stratified by diagnosis group. We also investigated the distribution of gabapentin administration frequency per day across diagnosis groups. Additionally, we explored the distribution of initial gabapentin prescription lengths by diagnosis group and evaluated the time to gabapentin discontinuation based on prescription refill patterns, specifically identifying discontinuation events where gaps between fills exceeded 30 days. Third, we calculated the adverse event rates per 100 patient-years for each diagnosis group and gabapentin dose category.

Finally, to evaluate the association between gabapentin dose and adverse events, we utilized the Cox-based recurrent event Anderson-Gill model. This choice was motivated by the need to manage the complexities associated with time-to-event data where multiple events per subject may occur. The gabapentin dose was modeled as a time-updated covariate, reflecting changes in prescription during follow-up. Models were adjusted for potential confounders based on clinical judgement, including age, sex, time on dialysis, the number of prior events, race, Hispanic ethnicity, primary cause of kidney failure, and the following drug classes: opioids, benzodiazepines, and antihistamines. To account for potential correlations between recurrent events within the same subject, the AG model uses the sandwich covariance estimator. To investigate potentially differential effects of gabapentin dose based on the diagnostic context, we performed this analysis both overall and stratified by time-updated diagnosis group. To formally assess these differential effects, we parameterized dose as a continuous variable and included an interaction term between dose and diagnosis category.

We repeated the analyses described above for pregabalin, another gabapentinoid medication. Because prescription prevalence is much higher for gabapentin than pregabalin, we focus mostly on gabapentin and provide pregabalin results in the supplementary appendix.

## Results

### Study Sample

This study utilized data from 533,232 HD patients included in the USRDS database between 2016-2020 (Fig 1). During the study period, diagnosis prevalence ranged from 7%-8% for pruritus and 29%-34% for neuropathic pain (Table S1). By the end of the study period, 9% of patients had diagnoses of both pruritus and neuropathic pain, 8% pruritus only, 28% neuropathic pain only, and 55% never had a diagnosis of either condition (neither). Patients in the pruritus-only group had a mean age of 61 versus 65 years for neuropathic pain only. The prevalence of diabetes was much higher among patients with neuropathic pain (75%) than with pruritus (47%); other comorbidities including atherosclerotic heart disease and congestive heart failure were also more common among patients with neuropathic pain versus pruritus. A detailed comparison of patient characteristics based on the first appearance in each diagnosis group over the study period is shown in Table 1, where patients can contribute to multiple diagnosis groups. Similarly, patients can contribute to multiple dose levels over the study period. Patients receiving higher (vs lower/none) doses of gabapentin were slightly younger and more likely to have diabetes during the time that they received the dose; minimal differences in other demographic and comorbid factors were observed across gabapentin dose over the study period (Table 2).

**Table 1:** Patient Characteristics, by Diagnosis Group

Diagnosis Group^a^				
Patient Characteristics	Neither	Pruritus Only	Neuropathic Pain Only	Both
**N patients**	498,510	49,646	172,869	41,343
**Patient-y**	623,123	68,040	305,925	68,068
**Age (y), mean ± SD**	64 ± 14	61 ± 15	65 ± 13	62 ± 13
**Female sex, %**	44%	51%	46%	53%
**Race %**				
White	60%	53%	61%	54%
Black	33%	39%	33%	40%
Other	7%	8%	6%	6%
**Hispanic ethnicity**	15%	14%	17%	17%
**Cause of kidney failure %**				
Diabetes	62%	51%	80%	76%
Hypertension	21%	24%	11%	13%
Glomerulonephritis	6%	10%	3%	5%
Other	11%	14%	5%	7%
**Comorbidity**^b^				
Amputation	3%	2%	4%	4%
Atherosclerotic heart disease	13%	10%	15%	12%
Malignant neoplasia, Cancer	6%	5%	5%	4%
Congestive heart failure	28%	24%	30%	28%
Chronic obstructive pulmonary disease	8%	7%	8%	7%
Cerebrovascular disease, CVA, TIA	9%	7%	9%	7%
Diabetes	58%	47%	75%	71%
History of hypertension	89%	88%	90%	89%
Inability to ambulate	6%	5%	6%	5%
Institutionalized	7%	6%	7%	6%
Institutionalized - Nursing Home	6%	5%	6%	5%
Needs assistance with daily activities	12%	10%	12%	10%
Other cardiac disease	18%	15%	17%	15%
Peripheral vascular disease	9%	7%	11%	10%
Current smoker	7%	8%	6%	7%

Abbreviations: CVA, cerebrovascular accident; TIA, transient ischemic attack.

a Patient characteristics as of 1st appearance in each diagnosis group; patients could contribute up to 3 records to the table (ie, neither -> pruritus -> both or neither -> neuropathic pain -> both) and so the number of patients in each group does not sum to the total number of unique patients included, which was N = 533,232.

b From the CMS 2,728 form as of start of dialysis.

**Table 2:** Patient Characteristics by Gabapentin Dose

Gabapentin Dose, mg/day						
Patient Characteristics	0	100	200	300	400-800	900+
**Dose range**	0	40-149	150-249	250-349	350-899	900-2700
**Patient-years**	908,072	23,545	21,043	44,270	26,787	26,437
**N patients**^a^	499,799	47,172	39,444	72,486	43,963	39,730
**Age (y), mean ± SD**	64 ± 14	65 ± 13	65 ± 13	63 ± 13	63 ± 12	61 ± 12
**Female sex, %**	47%	53%	54%	51%	51%	48%
**Race %**						
White	58%	56%	58%	57%	60%	59%
Black	35%	37%	36%	37%	34%	36%
Other/Unknown	7%	7%	6%	6%	6%	5%
**Hispanic ethnicity**	16%	17%	16%	17%	14%	15%
**Cause of kidney failure %**						
Diabetes	67%	73%	75%	76%	76%	79%
Hypertension	18%	13%	12%	12%	11%	11%
Glomerulonephritis	6%	6%	5%	5%	5%	4%
Other	10%	9%	8%	7%	8%	6%
**Comorbidity**^b^						
Amputation	4%	3%	4%	4%	5%	5%
Atherosclerotic heart disease	13%	14%	14%	13%	13%	13%
Malignant neoplasia, Cancer	5%	6%	5%	4%	4%	4%
Congestive heart failure	28%	27%	30%	31%	29%	30%
Chronic obstructive pulmonary disease	8%	9%	9%	10%	9%	10%
Cerebrovascular disease, CVA, TIA	8%	9%	10%	10%	9%	9%
Diabetes	63%	66%	67%	69%	71%	73%
History of hypertension	89%	88%	89%	89%	89%	89%
Inability to ambulate	6%	6%	6%	7%	6%	7%
Institutionalized	7%	8%	8%	7%	7%	7%
Institutionalized - Nursing Home	6%	7%	7%	6%	6%	6%
Needs assistance with daily activities	12%	11%	12%	11%	11%	12%
Other cardiac disease	17%	16%	17%	17%	16%	16%
Peripheral vascular disease	10%	11%	12%	11%	11%	12%
Tobacco use (current smoker)	7%	8%	8%	8%	8%	8%

Abbreviations: CVA, cerebrovascular accident; TIA, transient ischemic attack.

Each patient can contribute to multiple records in the table because each patient could have multiple prescription periods.

a Patient characteristics, N = 533,232 patients, in every prescription period in follow-up time between 2016 to 2020.

b From the CMS 2,728 form as of start of dialysis.

### Gabapentin Utilization

**Figure 2:**
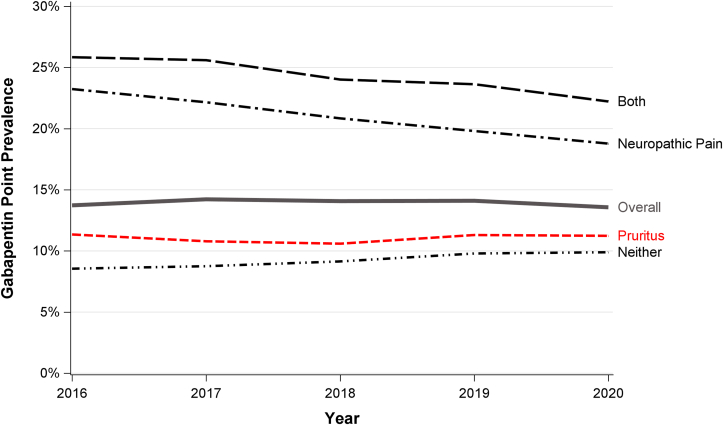
Gabapentin prescription prevalence by calendar year and diagnosis group. Active prescription as of December 31st of each year. Individual patients can contribute up to 5 total records, on December 31 of each year from 2016-2020, n = 416,074, unique HD patient (N = 1,063,373 total records) represented from 2016 to 2020 who were treated with HD on December 31 of any year. Diagnosis cumulatively determined based on updated diagnoses starting on January 1, 2016, or date of first dialysis from 2016 to 2020. HD, hemodialysis.

Gabapentin prescription prevalence remained consistent at about 14% across calendar years from 2016 to 2020 (Fig 2). In 2020, based on December 31 point prevalence, gabapentin use was 22% in the both group, 19% for neuropathic pain only, 11% for pruritus only, and 10% in the neither group. Gabapentin use appeared to decline over the study period in the both and neuropathic pain groups, but remained steady in the pruritus only group. Gabapentin administration frequency was concentrated at 1 (30%), 2 (26%), and 3 (30%) times per day, and initial prescription duration was concentrated at 30 days (63%) and 90 days (19%); these prescription patterns were consistent across diagnosis groups.

**Figure 3:**
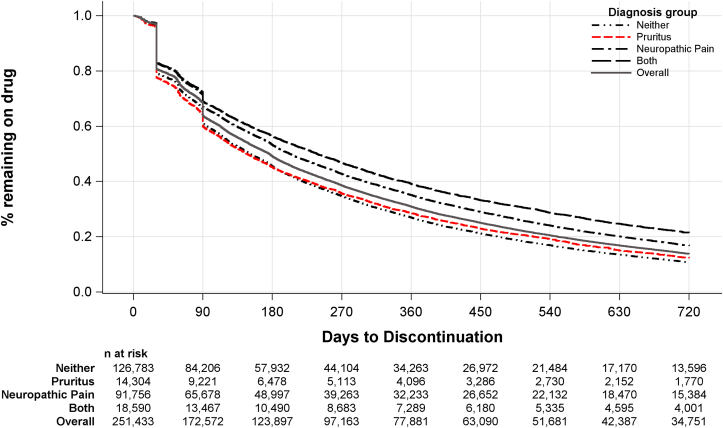
Time to gabapentin discontinuation.

Time-to-discontinuation for gabapentin initiators is illustrated in Fig 3. Sharp declines are observed at 30 and 90 days, reflecting initial prescriptions that are not renewed or refilled. The proportion of gabapentin initiators who remained on therapy was 49% after 6 months, 31% after 12 months, and 14% after 24 months. Discontinuation was more common among patients with pruritus than with neuropathic pain.

**Figure 4:**
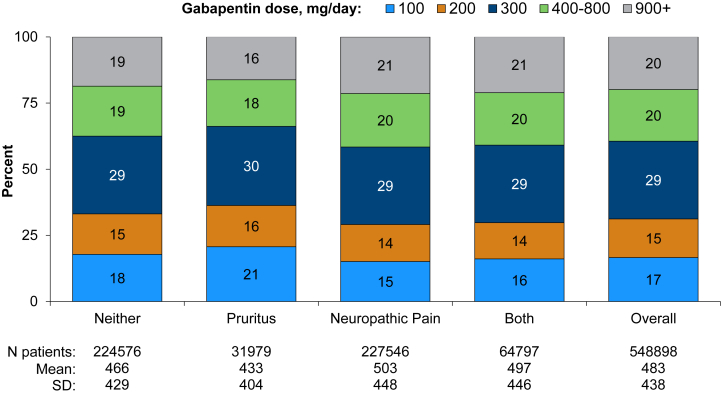
Gabapentin dose category, by diagnosis group. Dose information in every prescription period within each diagnosis period in follow-up time from 2016 to 2020. Diagnosis cumulatively determined based on updated diagnoses starting on January 1, 2016, or date of first dialysis from 2016 to 2020. N = 163,001, unique fee-for-service patients who are Gabapentin initiators, excluding patients who did not have any Gabapentin prescription from 2016 to 2020, and patients who had gabapentinoid (gabapentin or pregabalin) prescription in 1st month of 2016 and patients who already prescribed gabapentinoid (gabapentin or pregabalin) as of their first appearance in the dataset. About 6,466 (1.2%) missing or outlier dose records has been excluded from the bar plot, which is defined by <40 mg/day or >2,700 mg/day or actual missing.

**Figure 5:**
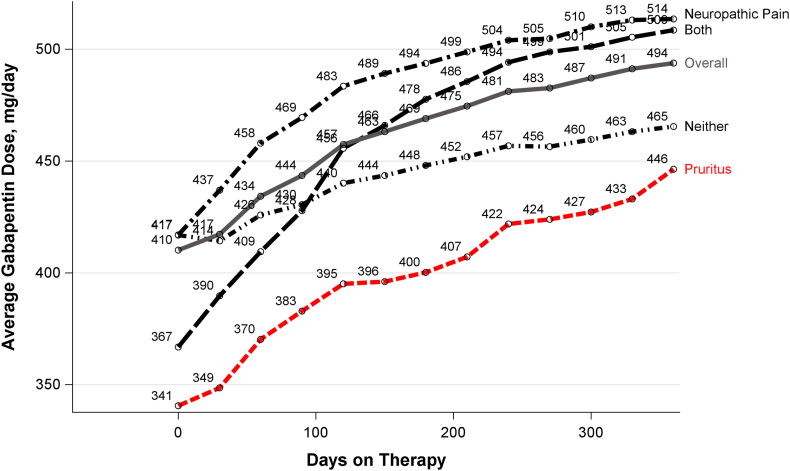
Gabapentin dose by time on therapy and diagnosis group; data shown for up to 1 year after prescription initiation.

The mean gabapentin dose was 483 (std dev: 438) mg/day overall; 39% of users were receiving at least 400 mg/day, and 20% of users were receiving at least 900 mg/day (Fig 4). The mean dose was higher among patients with neuropathic pain than pruritus (503 mg/day vs 433 mg/day). The mean gabapentin dose was 410 mg/day at initiation of therapy, and increased to 494 mg/day after 12 months on therapy. In all diagnosis groups, mean gabapentin dose increased with days on therapy (Fig 5).

### Gabapentin and Adverse Events

The overall event rates (per 100 patient-years) for the 5 adverse effects of interest were 37.8 for altered mental state, 20.4 for falls, 19.3 for dizziness, 15.2 for somnolence, and 9.3 for fracture. For all of these adverse events, rates were highest in the Both group, second highest in the neuropathic pain only group, third highest in the pruritus only group, and lowest in the neither group (Table 3). For altered mental state, falls, and somnolence, crude event rates increased with gabapentin dose overall and across subgroups (Fig S1).

**Table 3:** Absolute Event Rates (per 100 Patient-Years) by Diagnosis Group

Outcome	Event Rates per 100 Patient-Years					Total Patient-Y	N events
	Neither	Pruritus	Neuropathic Pain	Both	All		
Altered mental state	31.1	37.5	47.4	58.3	37.8	976,495	368,954
Dizziness	16.4	20.4	23.0	28.1	19.3	1,005,625	193,763
Falls	16.6	19.5	26.4	30.5	20.4	1,004,821	204,965
Fracture	7.7	8.9	11.9	13.0	9.3	1,029,353	95,554
Somnolence	12.3	15.3	19.4	24.2	15.2	1,020,943	155,612

**Figure 6:**
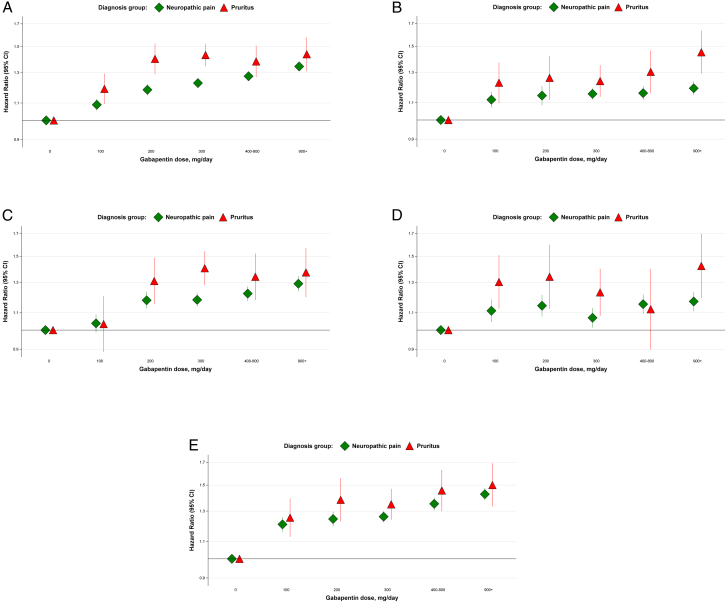
Association between gabapentin dose and adverse events, by diagnosis group. (A) Altered mental state; (B) Dizziness; (C) Somnolence; (D) Fracture; (E) Falls. The dose-response estimation is based on the Andersen-Gill model (an extension of the Cox proportional hazards model for analyzing recurrent event data. It allows for multiple event occurrences per subject, incorporates time-dependent covariates), adjusted by age, time on dialysis (year), the number of prior events, race (White, Black, Asian, and other/unknown), sex (female and male), hispanic (Yes and No/unknown), primary cause of kidney failure (diabetes, glomerulonephritis, hypertension, and other).

Overall, we observed elevated event rates among gabapentin users (vs non-users), with hazard ratios (HRs) showing a clear dose-response pattern for all 5 adverse effects (*P* < 0.001) (Table S2). In the pruritus only group, the associations were especially strong and more prominent than in the neuropathic pain only group or the both group (Fig 6). For example, the HRs comparing gabapentin dose of 900+ to 0 mg/day ranged from 1.37 to 1.50 for pruritus only, 1.17 to 1.43 for neuropathic pain only, and 1.18 to 1.33 for the both group. With the exception of somnolence (HR = 1.05), we observed a 20%-35% higher rate of events even for low-dose gabapentin (100 mg/day vs 0 mg/day) in the subset of patients with pruritus. We observed a stronger association between dose and adverse events in the pruritus-only group versus neuropathic pain only group for all outcomes (*P* for interaction < 0.01). For details of the association between gabapentin dose and adverse events—overall and within diagnosis groups—in adjusted models see Table S2.

### Pregabalin Utilization and Adverse Events

Pregabalin prevalence was lower compared to gabapentin and remained consistent across time from 2016-2020, with point prevalence (December 31) ranging from 2.2% to 2.5% and period prevalence (any prescription during the year) from 4.0% to 4.4%. Dosing patterns were similar to gabapentin, with dosing frequency concentrated at 1 (36%), 2 (42%), or 3 (18%) times per day, and the initial prescription length concentrated at 30 days (66%) and 90 (10%) days. The mean pregabalin dose was 130 mg/day overall; 57% of users were receiving at least 100 mg/day, and 85% of users were receiving at least 200 mg/day. The mean dose was higher among patients with neuropathic pain than pruritus (132 mg/day vs 115 mg/day). Similar to gabapentin, we observed a strong dose-response association between pregabalin and all five adverse effects in the overall cohort. In the subset of patients with pruritus, the association was especially strong for altered mental state, somnolence, and falls (Table S3).

## Discussion

In this study of over 500,000 US HD patients, we found that gabapentinoid utilization patterns differ by diagnosis group, with use and doses higher among patients with neuropathic pain vs. pruritus. We observed strong dose-response associations between gabapentinoid dose and adverse effects including altered mental status, dizziness, falls, fractures, and somnolence. These associations were especially strong among patients with pruritus; in this subgroup, adverse event rates were about 50% higher among patients receiving 900+ mg/day gabapentin versus non-users, and even relatively low-dose gabapentin—as low as 100-200 mg/day—was associated with a 20%-45% higher rate of adverse events.

These results were qualitatively consistent with other large cohort studies showing that gabapentin use was associated with hospitalization in the general population^20^ and in the nondialysis chronic kidney disease population.^5^ In the US dialysis setting, these findings build off of those by Ishida et al,^19^ who reported strong associations between gabapentinoids and adverse events including altered mental state, falls, and fracture. The dose-response associations observed in our overall 2016-2020 USRDS cohort were consistent with the 2011 USRDS cohort used by others.^19^ In addition to using more contemporary data, we extended the prior findings to focus on additional outcomes (dizziness and somnolence), explore diagnosis-specific subgroups (pruritus and neuropathic pain), and leverage the larger sample size (5-year cohort) to more granularly define the high-end range of gabapentin doses by splitting the highest exposure group in Ishida et al^19^ (>300 mg) into 400-800 mg and 900+ mg. These updates provide additional insights beyond simply updating the prior results in a contemporary cohort. Further, we found that, despite publication of the prior results in 2018, there were minimal changes in gabapentin prescription practices for pruritus from 2016-2020.

In the subset of patients with pruritus, the highest gabapentin doses (900+ mg/day, 16% of patients) were strongly associated with all 5 of the adverse events tested, with HRs ranging from 1.48 to 1.66. In the neuropathic pain subset, the gabapentin doses of 900+ mg/day were also associated with a greater risk of adverse events, though the magnitudes of the associations were smaller. In both groups, we observed a clear dose-response pattern, with elevated risk at even the lowest observed gabapentin dose levels (100-200 mg/day). For altered mental status, falls, and somnolence, absolute event rates were higher in the neuropathic pain versus pruritus group, and similarly increased in a dose-response pattern (Fig S1). Overall, the event rates (per 100 patient-years) were 37.8 for altered mental status, 20.4 for falls, 19.3 for dizziness, 15.2 for somnolence, and 9.3 for fractures—affecting a substantial proportion of the dialysis population. Generally, in scenarios where the event rate is high (vs relatively low), a greater number of cases can be attributed to the exposure. Thus, modifying risk factors—even with modest associations—can substantially reduce the total number of events, and consequently, health care costs associated with these events.

Although gabapentinoid prescription practices have been described, this study stratifies these findings by diagnosis group as a proxy for treatment indication. Gabapentin use was about twice as high among patients with neuropathic pain compared with pruritus (∼20% vs 10%) (Fig 2), though mean dose was only about 15% higher (503 vs 443 mg/day) in the neuropathic pain versus pruritus group. The proportion of gabapentin users receiving at least 300 mg/day was 70% in the neuropathic pain group; we had anticipated substantially lower doses in the pruritus group, but even so, 64% were receiving 300+ mg/day and 16% were receiving 900+ mg/day (Fig 3). These doses far exceed the label-recommended maximum of 300-350 mg/day.^9^

The implications of higher adverse event rates for off-label gabapentinoid prescriptions to treat pruritus are relevant to HD patients and providers, particularly in the context of effective therapies with favorable risk-benefit profiles.^21^ Although gabapentin doses were lower in the pruritus (vs neuropathic pain) group, these doses—even as low as 100-200 mg/day—were still associated with adverse events, particularly among patients with pruritus. The adverse effects of gabapentin are well-known,^2^ supported by our results, and should motivate consideration of alternative therapies. However, our data suggest that pregabalin is not the solution either, with a adverse effect profile similar to gabapentin (Table S1). Consideration of alternative therapies to treat neuropathic pain is recommended as well, though other options (eg, opioids, antidepressants, topical creams, and off-label anti-seizure medications) have their own limitations.

A key strength of this study is the large sample size (N > 500,000); this allowed us to define and analyze granular dosing groups, even in small subgroups of patients with a diagnosis of neuropathic pain or pruritus, thus revealing strong and clear dose-response associations. Other strengths include the contemporary (2016-2020) nature of the data, and the claims data where prescriptions are well-recorded and adverse events are well-captured via ICD-10 codes.

Our study also had some limitations. First, we observed clear and substantial under-ascertainment of pruritus when relying on diagnosis claims: 7%-9% each year, compared with 35%-40% of patients indicating being at least moderately bothered by itchy skin when asked directly.^16^ This misclassification is likely to be primarily in one direction; patients with a diagnosis of pruritus are almost certainly bothered by itch, while many patients without a diagnosis are likely still bothered by itch. We thus mostly avoided comparison and interpretation of the neither group in our study, which likely includes many patients with symptomatic but undiagnosed pruritus. Second, even if perfectly captured, these diagnoses would not be a perfect proxy for indication/motivation to treat, which is unknown and generally not recorded. This is especially complicated for the both group of patients with a diagnosis of both neuropathic pain and pruritus; we thus also generally avoided comparison and interpretation of this group, instead focusing on the neuropathic pain only and pruritus only groups. Third, as in any observational study, residual confounding may bias results when the treatment groups are not randomized. In addition to extensive covariate adjustment, our subgroup analyses inherently reduce this potential bias. By restricting to patients with a diagnosis of pruritus (or neuropathic pain), our reference groups (diagnosis of pruritus or neuropathic pain + no gabapentin) should be more comparable to treated patients than in an analysis of the overall cohort where many untreated patients would not even be candidates for gabapentinoid therapy.

In conclusion, in this large cohort of US HD patients, we found that gabapentin use was twice as high—with only slightly higher doses—in patients diagnosed with neuropathic pain vs pruritus. We observed a strong dose-response association between gabapentin and adverse effects, including altered mental status, dizziness, falls, fractures, and somnolence, with ∼50% increased risk at doses of 900+ mg/day. Even gabapentin doses as low as 100-200 mg/day were associated with adverse events, especially when used off-label among patients with a diagnosis of pruritus and no diagnosis of neuropathic pain. With new alternatives available, prescribers should consider the clinically relevant adverse effects of gabapentinoids when providing treatment options to their patients with pruritus.

## Footnotes

Supplementary File (PDF)
**Appendix A.** ICD-10 and ICD-9 codes for adverse events including (a) Altered mental state; (b) Dizziness; (c) Somnolence; (d) Fracture; and (e) Falls. **Appendix B.** ICD-10 codes for diagnosis groups: (a) pruritus, (b) neuropathic pain. **Figure S1:** Adverse event rates, by gabapentin dose and diagnosis group. **Table S1:** Pruritus and neuropathic pain diagnosis prevalence by year. **Table S2:** Association between gabapentin dose and adverse events. **Table S3:** Association between pregabalin dose and adverse events.
