# Supplementary material for: Gabapentin Utilization and Adverse Effects Among US Hemodialysis Patients Diagnosed With Pruritus or Neuropathic Pain
Source: Kidney Med. 2026 Mar 18;8(5):101341. doi: 10.1016/j.xkme.2026.101341 (PMC13098409; doi:10.1016/j.xkme.2026.101341)
Supplement: Supplementary File (PDF) — Appendix A-B; Figure S1; Table S1-S3. [file mmc1.pdf]

Supplementary Table of Contents

Figure S1. Adverse event rates, by gabapentin dose and diagnosis group

Table S1. Pruritus and neuropathic pain diagnosis prevalence by year

Table S2. Association between gabapentin dose and adverse events

Table S3. Association between pregabalin dose and adverse events

Appendix A. ICD-10 and ICD-9 codes for adverse events including (a) Altered mental state; (b) Dizziness; (c) Somnolence; (d) Fracture; and (e) Falls

Appendix B. ICD-10 codes for diagnosis groups: (a) pruritus, (b) neuropathic pain

Supplementary Material

Figure S1

Figure S1. Adverse event rates, by gabapentin dose and diagnosis group

(a) Altered mental state

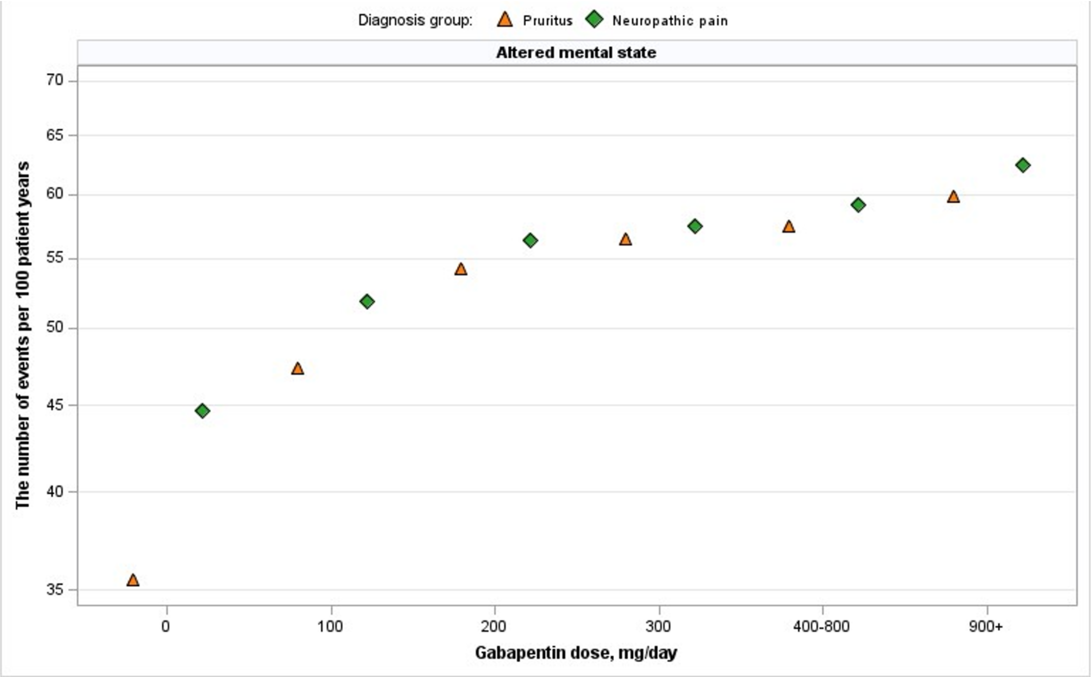

(b) Dizziness

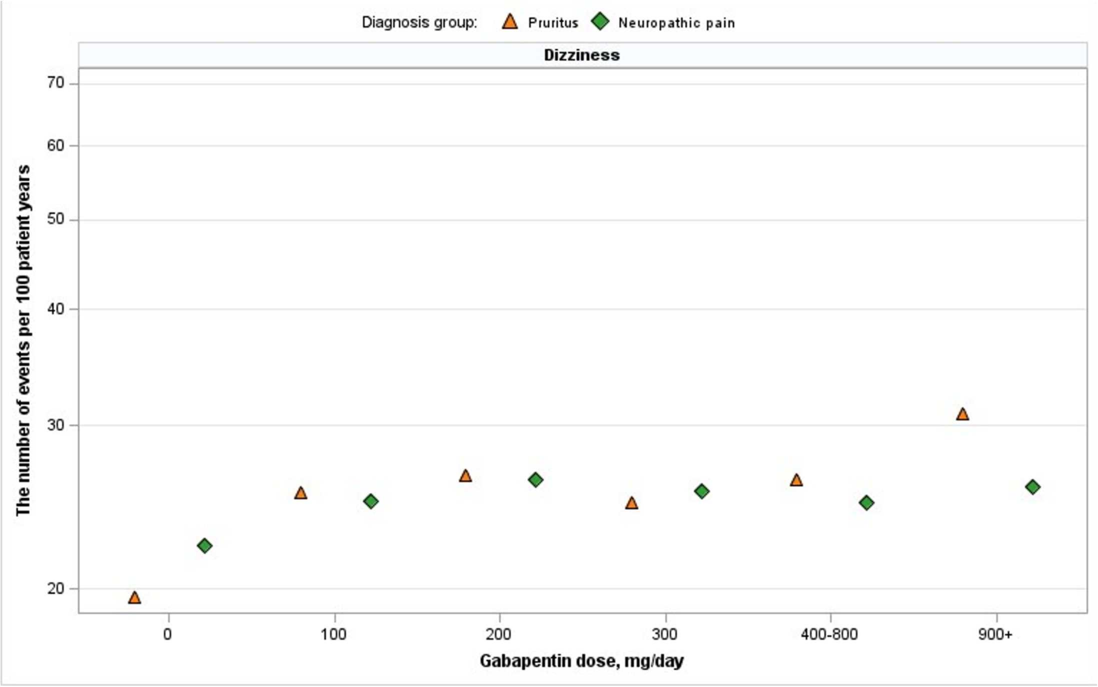

(c) Somnolence

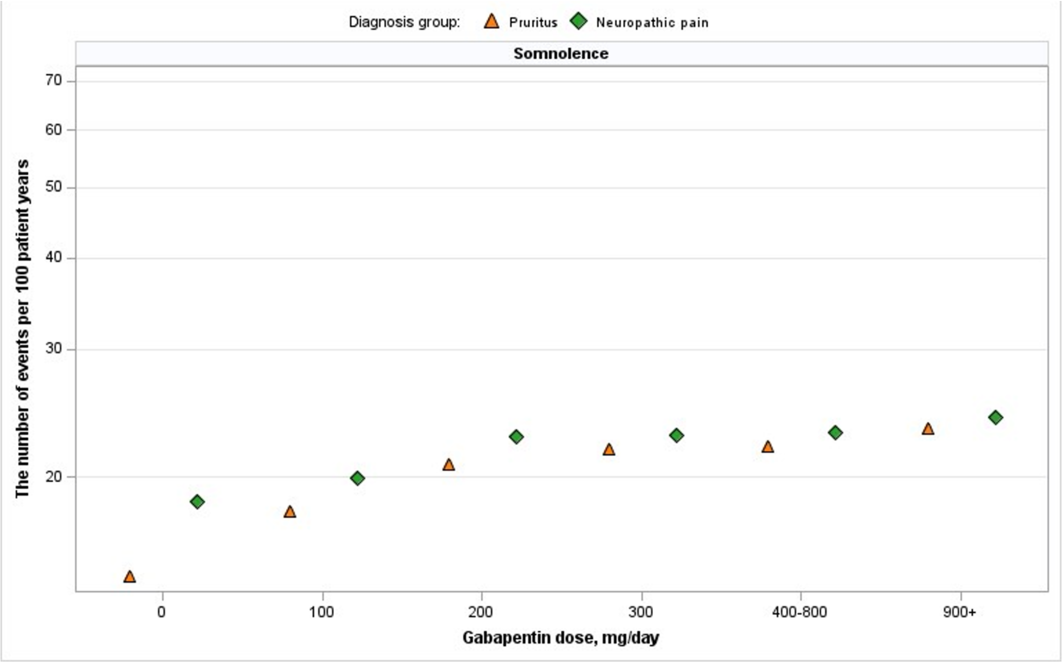

(d) Fracture

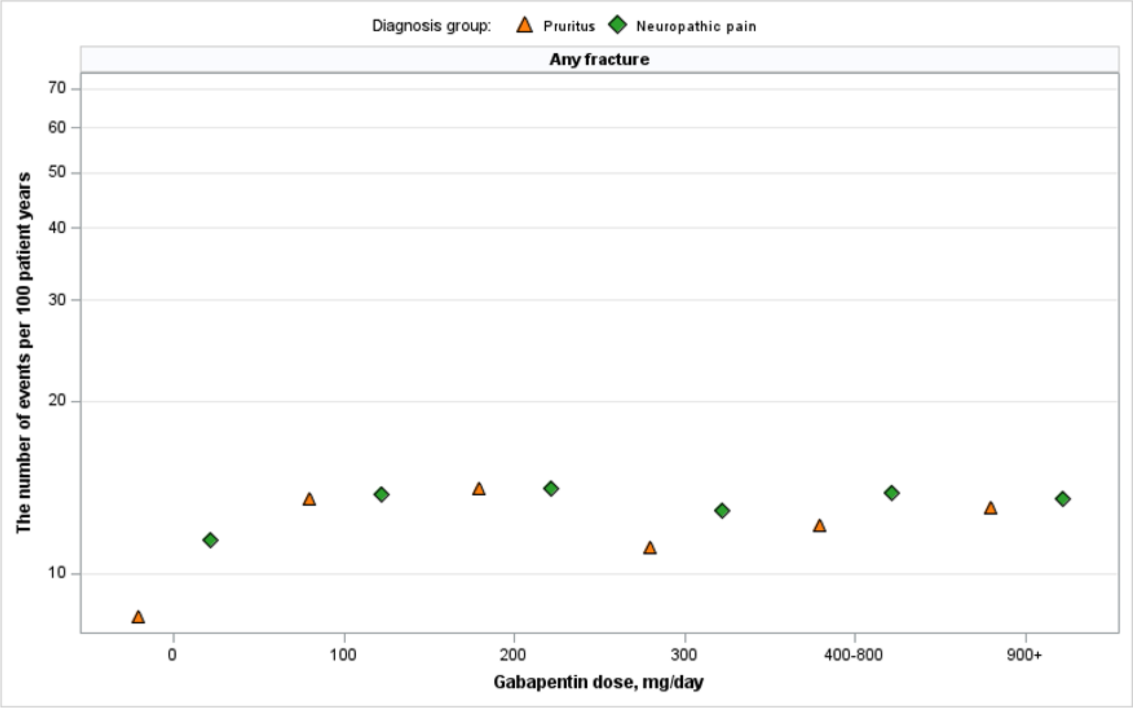

(e) Falls

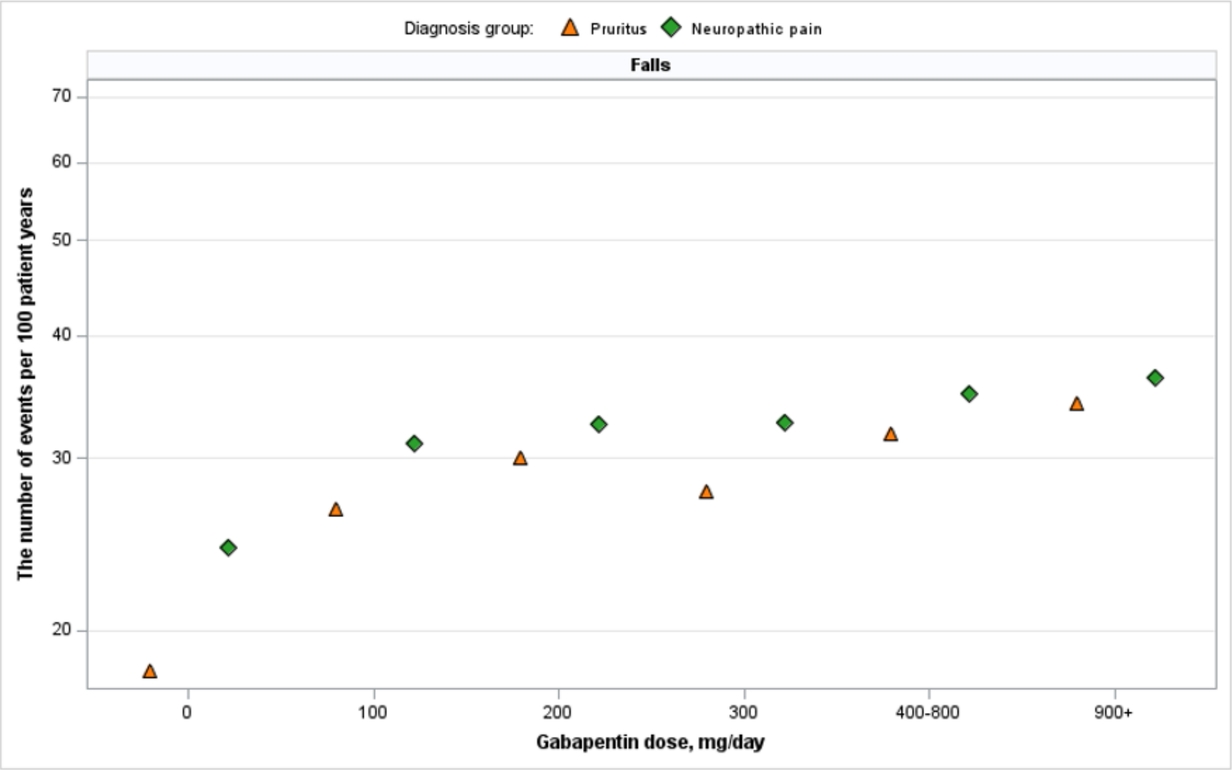
Diagnosis group:
Pruritus
Neuropathic pain
Falls
70
60
50
40
30
20
The number of events per 100 patient years
0
100
200
300
400-800
900+
Gabapentin dose, mg/day

Table S1

Table S1. Pruritus and neuropathic pain diagnosis prevalence by year

Year	Diagnosis		
	Pruritus	Neuropathic pain	N patients
2016	21,142 (7.2%)	89,853 (30.6%)	293,636
2017	22,467 (7.6%)	100,215 (33.9%)	295,619
2018	23,159 (8.0%)	95,820 (33.1%)	289,485
2019	23,390 (8.3%)	87,643 (31.1%)	281,809
2020	22,705 (8.5%)	77,731 (29.1%)	267,116
All	42,125 (7.9%)	168,501 (31.6%)	533,232

Table S2

Table S2. Association[1] (hazard ratio, 95% CI) between gabapentin dose and adverse events, overall and by diagnosis group

	All	Pruritus only	Neuropathic pain only	Both pruritus and neuropathic pain
**Altered mental state**				
**Dose = 0**	1(ref)	1(ref)	1(ref)	1(ref)
Dose 100	1.16(1.13-1.18)	1.19(1.09-1.29)	1.09(1.05-1.13)	1.10(1.05-1.17)
Dose 200	1.27(1.24-1.30)	1.40(1.29-1.53)	1.18(1.15-1.22)	1.15(1.09-1.22)
Dose 300	1.31(1.28-1.33)	1.43(1.35-1.52)	1.23(1.20-1.26)	1.14(1.09-1.18)
Dose 400-800	1.37(1.35-1.40)	1.38(1.27-1.51)	1.27(1.24-1.31)	1.31(1.25-1.37)
Dose 900+	1.45(1.42-1.47)	1.44(1.31-1.58)	1.35(1.31-1.38)	1.29(1.23-1.36)
**Dizziness**				
**Dose = 0**	1(ref)	1(ref)	1(ref)	1(ref)
Dose 100	1.18(1.15-1.22)	1.23(1.10-1.37)	1.12(1.07-1.17)	1.06(0.97-1.15)
Dose 200	1.23(1.19-1.27)	1.26(1.12-1.42)	1.14(1.08-1.21)	1.17(1.07-1.27)
Dose 300	1.25(1.22-1.28)	1.24(1.14-1.35)	1.15(1.12-1.19)	1.12(1.06-1.19)
Dose 400-800	1.24(1.21-1.28)	1.30(1.16-1.47)	1.16(1.12-1.20)	1.11(1.03-1.19)
Dose 900+	1.32(1.29-1.36)	1.45(1.29-1.63)	1.19(1.15-1.24)	1.22(1.14-1.30)
**Somnolence**				
**Dose = 0**	1(ref)	1(ref)	1(ref)	1(ref)
Dose 100	1.08(1.05-1.12)	1.03(0.89-1.21)	1.04(0.99-1.09)	0.99(0.90-1.09)
Dose 200	1.26(1.22-1.30)	1.31(1.15-1.49)	1.18(1.12-1.23)	1.18(1.08-1.28)
Dose 300	1.27(1.24-1.31)	1.40(1.28-1.54)	1.18(1.14-1.22)	1.13(1.06-1.20)
Dose 400-800	1.31(1.28-1.35)	1.34(1.18-1.52)	1.22(1.17-1.27)	1.25(1.16-1.34)
Dose 900+	1.40(1.36-1.44)	1.37(1.20-1.57)	1.29(1.24-1.34)	1.19(1.11-1.29)
**Any fracture**				
**Dose = 0**	1(ref)	1(ref)	1(ref)	1(ref)
Dose 100	1.15(1.10-1.21)	1.30(1.12-1.51)	1.11(1.04-1.18)	1.01(0.89-1.15)
Dose 200	1.19(1.14-1.24)	1.34(1.12-1.60)	1.14(1.08-1.21)	1.11(0.97-1.27)
Dose 300	1.19(1.15-1.23)	1.23(1.08-1.40)	1.07(1.01-1.13)	1.16(1.08-1.26)
Dose 400-800	1.21(1.15-1.26)	1.12(0.90-1.40)	1.15(1.09-1.22)	1.02(0.90-1.17)
Dose 900+	1.31(1.26-1.36)	1.42(1.19-1.70)	1.17(1.11-1.23)	1.18(1.08-1.30)
**Falls**				
**Dose = 0**	1(ref)	1(ref)	1(ref)	1(ref)
Dose 100	1.25(1.22-1.29)	1.25(1.13-1.39)	1.21(1.16-1.26)	1.08(1.00-1.16)
Dose 200	1.31(1.28-1.35)	1.38(1.23-1.56)	1.24(1.19-1.30)	1.21(1.11-1.33)

Dose 300	1.36(1.33-1.39)	1.35(1.24-1.47)	1.26(1.22-1.30)	1.23(1.17-1.30)
Dose 400-800	1.43(1.40-1.47)	1.46(1.30-1.63)	1.35(1.31-1.40)	1.30(1.22-1.39)
Dose 900+	1.53(1.49-1.57)	1.50(1.33-1.69)	1.43(1.38-1.47)	1.33(1.24-1.44)

1. The dose-response estimation is based on the Andersen-Gill model (an extension of the Cox proportional hazards model for analyzing recurrent event data. It allows for multiple event occurrences per subject, incorporates time-dependent covariates) , adjusted by age, time on dialysis (year), the number of prior events, race (White, Black, Asian, other/unknown), sex (female, male), hispanic (Yes, No/unknown), primary cause of kidney failure(Diabetes, Glomerulonephritis, Hypertension and other), and the following drug classes: opioids, benzodiazepines, and antihistamines.

Table S3

Table S3. Association[1] (hazard ratio, 95% CI) between pregabalin dose and adverse events, overall and by diagnosis group

New Dose Categorization				
Outcome/Dose	**All**	**Pruritus only**	**Neuropathic pain only**	**Both Pruritus and Neuropathic pain**
**Altered mental state**				
**Dose = 0**	1(ref)	1(ref)	1(ref)	1(ref)
Dose 25-75	1.19(1.15-1.22)	1.13(0.99-1.30)	1.17(1.13-1.22)	1.02(0.95-1.10)
Dose 100-150	1.34(1.29-1.39)	1.34(1.17-1.54)	1.28(1.22-1.34)	1.28(1.19-1.37)
Dose 200+	1.47(1.41-1.52)	1.84(1.54-2.18)	1.45(1.38-1.52)	1.36(1.25-1.48)
**Dizziness**				
**Dose = 0**	1(ref)	1(ref)	1(ref)	1(ref)
Dose 25-75	1.21(1.16-1.26)	1.06(0.88-1.29)	1.18(1.11-1.25)	1.07(0.95-1.20)
Dose 100-150	1.26(1.21-1.32)	1.08(0.87-1.34)	1.22(1.15-1.30)	1.12(1.01-1.25)
Dose 200+	1.26(1.19-1.34)	1.20(0.90-1.61)	1.23(1.13-1.32)	1.18(1.05-1.34)
**Somnolence**				
**Dose = 0**	1(ref)	1(ref)	1(ref)	1(ref)
Dose 25-75	1.16(1.11-1.22)	1.08(0.87-1.34)	1.11(1.04-1.18)	1.03(0.92-1.15)
Dose 100-150	1.26(1.19-1.32)	1.21(0.96-1.53)	1.11(1.02-1.19)	1.17(1.05-1.30)
Dose 200+	1.41(1.32-1.50)	1.68(1.27-2.23)	1.35(1.25-1.46)	1.35(1.19-1.52)
**Any fracture**				
**Dose = 0**	1(ref)	1(ref)	1(ref)	1(ref)
Dose 25-75	1.21(1.14-1.29)	1.26(0.98-1.62)	1.15(1.06-1.25)	1.12(0.97-1.29)
Dose 100-150	1.20(1.13-1.28)	0.96(0.69-1.33)	1.06(0.97-1.15)	1.18(1.02-1.37)
Dose 200+	1.29(1.20-1.40)	1.17(0.76-1.79)	1.21(1.09-1.34)	1.05(0.87-1.27)
**Falls**				
**Dose = 0**	1(ref)	1(ref)	1(ref)	1(ref)
Dose 25-75	1.24(1.19-1.29)	1.36(1.14-1.63)	1.20(1.14-1.27)	1.08(0.97-1.21)
Dose 100-150	1.35(1.29-1.40)	1.26(1.03-1.53)	1.28(1.21-1.35)	1.27(1.15-1.39)
Dose 200+	1.50(1.42-1.57)	1.43(1.11-1.84)	1.34(1.25-1.44)	1.47(1.31-1.63)

1. The dose-response estimation is based on the Andersen-Gill model (an extension of the Cox proportional hazards model for analyzing recurrent event data. It allows for multiple event occurrences per subject, incorporates time-dependent covariates) , adjusted by age, time on dialysis (year), the number of prior events, race (White, Black, Asian, other/unknown), sex (female, male), hispanic (Yes, No/unknown), primary cause of kidney failure (Diabetes, Glomerulonephritis, Hypertension and other), and the following drug classes: opioids, benzodiazepines, and antihistamines.

Appendix A

ICD-10 and ICD-9 codes for adverse events including (a) Altered mental state; (b) Dizziness; (c) Somnolence; (d) Fracture; and (e) Falls

(a) Altered mental state

F05	Delirium due to known physiological condition
F29	Unspecified psychosis not due to a substance or known physiological condition
G92	Toxic encephalopathy
G9340	Encephalopathy, unspecified
G9349	Other encephalopathy
I6783	Posterior reversible encephalopathy syndrome
R400	Somnolence
R401	Stupor
R404	Transient alteration of awareness
R410	Disorientation, unspecified
R4182	Altered mental status, unspecified
2929	Unspecified drug-induced mental disorder
2930	Delirium due to conditions classified elsewhere
2931	Subacute delirium
2989	Unspecified psychosis
34830	Encephalopathy, unspecified
34839	Other encephalopathy
34982	Toxic encephalopathy
78002	Transient alteration of awareness
78009	Other alteration of consciousness
78097	Altered mental status

(a)

(b) Dizziness

Code	Description
R42	Dizziness and giddiness
7804	Dizziness and giddiness

(c) Somnolence

Code	Description
R400	Somnolence
R401	Stupor
R4020	Unspecified coma
R402110	Coma scale, eyes open, never, unspecified time
R402111	Coma scale, eyes open, never, in the field [EMT or ambulance]
R402112	Coma scale, eyes open, never, at arrival to emergency department
R402113	Coma scale, eyes open, never, at hospital admission

R402114	Coma scale, eyes open, never, 24 hours or more after hospital admission
R402120	Coma scale, eyes open, to pain, unspecified time
R402121	Coma scale, eyes open, to pain, in the field [EMT or ambulance]
R402122	Coma scale, eyes open, to pain, at arrival to emergency department
R402123	Coma scale, eyes open, to pain, at hospital admission
R402124	Coma scale, eyes open, to pain, 24 hours or more after hospital admission
R402130	Coma scale, eyes open, to sound, unspecified time
R402131	Coma scale, eyes open, to sound, in the field [EMT or ambulance]
R402132	Coma scale, eyes open, to sound, at arrival to emergency department
R402133	Coma scale, eyes open, to sound, at hospital admission
R402134	Coma scale, eyes open, to sound, 24 hours or more after hospital admission
R402140	Coma scale, eyes open, spontaneous, unspecified time
R402141	Coma scale, eyes open, spontaneous, in the field [EMT or ambulance]
R402142	Coma scale, eyes open, spontaneous, at arrival to emergency department
R402143	Coma scale, eyes open, spontaneous, at hospital admission
R402144	Coma scale, eyes open, spontaneous, 24 hours or more after hospital admission
R402210	Coma scale, best verbal response, none, unspecified time
R402211	Coma scale, best verbal response, none, in the field [EMT or ambulance]
R402212	Coma scale, best verbal response, none, at arrival to emergency department
R402213	Coma scale, best verbal response, none, at hospital admission
R402214	Coma scale, best verbal response, none, 24 hours or more after hospital admission
R402220	Coma scale, best verbal response, incomprehensible words, unspecified time
R402221	Coma scale, best verbal response, incomprehensible words, in the field [EMT or ambulance]
R402222	Coma scale, best verbal response, incomprehensible words, at arrival to emergency department
R402223	Coma scale, best verbal response, incomprehensible words, at hospital admission
R402224	Coma scale, best verbal response, incomprehensible words, 24 hours or more after hospital admission
R402230	Coma scale, best verbal response, inappropriate words, unspecified time
R402231	Coma scale, best verbal response, inappropriate words, in the field [EMT or ambulance]
R402232	Coma scale, best verbal response, inappropriate words, at arrival to emergency department
R402233	Coma scale, best verbal response, inappropriate words, at hospital admission
R402234	Coma scale, best verbal response, inappropriate words, 24 hours or more after hospital admission
R402240	Coma scale, best verbal response, confused conversation, unspecified time
R402241	Coma scale, best verbal response, confused conversation, in the field [EMT or ambulance]
R402242	Coma scale, best verbal response, confused conversation, at arrival to emergency department
R402243	Coma scale, best verbal response, confused conversation, at hospital admission
R402244	Coma scale, best verbal response, confused conversation, 24 hours or more after hospital admission

R402250	Coma scale, best verbal response, oriented, unspecified time
R402251	Coma scale, best verbal response, oriented, in the field [EMT or ambulance]
R402252	Coma scale, best verbal response, oriented, at arrival to emergency department
R402253	Coma scale, best verbal response, oriented, at hospital admission
R402254	Coma scale, best verbal response, oriented, 24 hours or more after hospital admission
R402310	Coma scale, best motor response, none, unspecified time
R402311	Coma scale, best motor response, none, in the field [EMT or ambulance]
R402312	Coma scale, best motor response, none, at arrival to emergency department
R402313	Coma scale, best motor response, none, at hospital admission
R402314	Coma scale, best motor response, none, 24 hours or more after hospital admission
R402320	Coma scale, best motor response, extension, unspecified time
R402321	Coma scale, best motor response, extension, in the field [EMT or ambulance]
R402322	Coma scale, best motor response, extension, at arrival to emergency department
R402323	Coma scale, best motor response, extension, at hospital admission
R402324	Coma scale, best motor response, extension, 24 hours or more after hospital admission
R402330	Coma scale, best motor response, abnormal flexion, unspecified time
R402331	Coma scale, best motor response, abnormal flexion, in the field [EMT or ambulance]
R402332	Coma scale, best motor response, abnormal flexion, at arrival to emergency department
R402333	Coma scale, best motor response, abnormal flexion, at hospital admission
R402334	Coma scale, best motor response, abnormal flexion, 24 hours or more after hospital admission
R402340	Coma scale, best motor response, flexion withdrawal, unspecified time
R402341	Coma scale, best motor response, flexion withdrawal, in the field [EMT or ambulance]
R402342	Coma scale, best motor response, flexion withdrawal, at arrival to emergency department
R402343	Coma scale, best motor response, flexion withdrawal, at hospital admission
R402344	Coma scale, best motor response, flexion withdrawal, 24 hours or more after hospital admission
R402350	Coma scale, best motor response, localizes pain, unspecified time
R402351	Coma scale, best motor response, localizes pain, in the field [EMT or ambulance]
R402352	Coma scale, best motor response, localizes pain, at arrival to emergency department
R402353	Coma scale, best motor response, localizes pain, at hospital admission
R402354	Coma scale, best motor response, localizes pain, 24 hours or more after hospital admission
R402360	Coma scale, best motor response, obeys commands, unspecified time
R402361	Coma scale, best motor response, obeys commands, in the field [EMT or ambulance]
R402362	Coma scale, best motor response, obeys commands, at arrival to emergency department
R402363	Coma scale, best motor response, obeys commands, at hospital admission
R402364	Coma scale, best motor response, obeys commands, 24 hours or more after hospital admission
R402410	Glasgow coma scale score 13-15, unspecified time
R402411	Glasgow coma scale score 13-15, in the field [EMT or ambulance]
R402412	Glasgow coma scale score 13-15, at arrival to emergency department
R402413	Glasgow coma scale score 13-15, at hospital admission

R402414	Glasgow coma scale score 13-15, 24 hours or more after hospital admission
R402420	Glasgow coma scale score 9-12, unspecified time
R402421	Glasgow coma scale score 9-12, in the field [EMT or ambulance]
R402422	Glasgow coma scale score 9-12, at arrival to emergency department
R402423	Glasgow coma scale score 9-12, at hospital admission
R402424	Glasgow coma scale score 9-12, 24 hours or more after hospital admission
R402430	Glasgow coma scale score 3-8, unspecified time
R402431	Glasgow coma scale score 3-8, in the field [EMT or ambulance]
R402432	Glasgow coma scale score 3-8, at arrival to emergency department
R402433	Glasgow coma scale score 3-8, at hospital admission
R402434	Glasgow coma scale score 3-8, 24 hours or more after hospital admission
R402440	Other coma, without documented Glasgow coma scale score, or with partial score reported, unspecified time
R402441	Other coma, without documented Glasgow coma scale score, or with partial score reported, in the field [EMT or ambulance]
R402442	Other coma, without documented Glasgow coma scale score, or with partial score reported, at arrival to emergency department
R402443	Other coma, without documented Glasgow coma scale score, or with partial score reported, at hospital admission
R402444	Other coma, without documented Glasgow coma scale score, or with partial score reported, 24 hours or more after hospital admission
R402A	Nontraumatic coma due to underlying condition
R403	Persistent vegetative state
R404	Transient alteration of awareness
78001	Coma
78002	Trans alter awareness
78003	Persistent vegtv state
78009	Other alter consciousnes

(d) Fracture

Code	Description
7855	Internal fixation of femur without fracture reduction
7905	Closed reduction of fracture of femur without internal fixation
7915	Closed reduction of fracture of femur with internal fixation
7925	Open reduction of fracture of femur without internal fixation
7935	Open reduction of fracture of femur with internal fixation
7965	Debridement of open fracture of femur
8052	Closed fracture of dorsal (thoracic) vertebra without mention of spinal cord injury
8053	Open fracture of dorsal (thoracic) vertebra without mention of spinal cord injury
8054	Closed fracture of lumbar vertebra without mention of spinal cord injury
8055	Open fracture of lumbar vertebra without mention of spinal cord injury
8056	Closed fracture of sacrum and coccyx without mention of spinal cord injury
8057	Open fracture of sacrum and coccyx without mention of spinal cord injury

8058	Closed fracture of unspecified vertebral column without mention of spinal cord injury
8059	Open fracture of unspecified part of vertebral column without mention of spinal cord injury
8064	Closed fracture of lumbar spine with spinal cord injury
8065	Open fracture of lumbar spine with spinal cord injury
8068	Closed fracture of unspecified vertebra with spinal cord injury
8069	Open fracture of unspecified vertebra with spinal cord injury
8080	Closed fracture of acetabulum
8081	Open fracture of acetabulum
8082	Closed fracture of pubis
8083	Open fracture of pubis
8088	Closed unspecified fracture of pelvis
8089	Open unspecified fracture of pelvis
8208	Closed fracture of unspecified part of neck of femur
8209	Open fracture of unspecified part of neck of femur
8240	Closed fracture of medial malleolus
8241	Open fracture of medial malleolus
8242	Closed fracture of lateral malleolus
8243	Open fracture of lateral malleolus
8244	Closed bimalleolar fracture
8245	Open bimalleolar fracture
8246	Closed trimalleolar fracture
8247	Open trimalleolar fracture
8248	Unspecified closed fracture of ankle
8249	Unspecified open fracture of ankle
8250	Closed fracture of calcaneus
8251	Open fracture of calcaneus
22305	Closed tx spine process fx
22310	Closed tx vert fx w/o manj
22315	Closed tx vert fx w/manj
22318	Treat odontoid fx w/o graft
22319	Treat odontoid fx w/graft
22325	Treat spine fracture
22326	Treat neck spine fracture
22327	Treat thorax spine fracture
22328	Treat each add spine fx
22510	Perq cervicothoracic inject
22511	Perq lumbosacral injection
22512	Vertebroplasty addl inject
22513	Perq vertebral augmentation
22514	Perq vertebral augmentation
22515	Perq vertebral augmentation
22520	Percut vertebroplasty thor
22521	Percut vertebroplasty lumb

22522	Percut vertebroplasty addl
22523	Percut kyphoplasty thor
22524	Percut kyphoplasty lumbar
22525	Percut kyphoplasty add-on
23500	Closed treatment of clavicular fracture; without manipulation
23505	Closed treatment of clavicular fracture; with manipulation
23515	Open treatment of clavicular fracture, includes internal fixation, when performed
23600	Closed treatment of proximal humeral (surgical or anatomical neck) fracture; without manipulation
23605	Closed treatment of proximal humeral (surgical or anatomical neck) fracture; with manipulation, with or without skeletal traction
23615	Open treatment of proximal humeral (surgical or anatomical neck) fracture, includes internal fixation, when performed, includes repair of tuberosity(s), when performed;
23616	Open treatment of proximal humeral (surgical or anatomical neck) fracture, includes internal fixation, when performed, includes repair of tuberosity(s), when performed; with proximal humeral prosthetic replacement
23620	Closed treatment of greater humeral tuberosity fracture; without manipulation
23625	Closed treatment of greater humeral tuberosity fracture; with manipulation
23630	Open treatment of greater humeral tuberosity fracture, includes internal fixation, when performed
23665	Closed treatment of shoulder dislocation, with fracture of greater humeral tuberosity, with manipulation
23670	Open treatment of shoulder dislocation, with fracture of greater humeral tuberosity, includes internal fixation, when performed
23675	Closed treatment of shoulder dislocation, with surgical or anatomical neck fracture, with manipulation
23680	Open treatment of shoulder dislocation, with surgical or anatomical neck fracture, includes internal fixation, when performed
24500	Closed treatment of humeral shaft fracture; without manipulation
24505	Closed treatment of humeral shaft fracture; with manipulation, with or without skeletal traction
24515	Open treatment of humeral shaft fracture with plate/screws, with or without cerclage
24516	Treatment of humeral shaft fracture, with insertion of intramedullary implant, with or without cerclage and/or locking screws
24530	Closed treatment of supracondylar or transcondylar humeral fracture, with or without intercondylar extension; without manipulation
24535	Closed treatment of supracondylar or transcondylar humeral fracture, with or without intercondylar extension; with manipulation, with or without skin or skeletal traction
24538	Percutaneous skeletal fixation of supracondylar or transcondylar humeral fracture, with or without intercondylar extension
24545	Open treatment of humeral supracondylar or transcondylar fracture, includes internal fixation, when performed; without intercondylar extension
24546	Open treatment of humeral supracondylar or transcondylar fracture, includes internal fixation, when performed; with intercondylar extension
24560	Closed treatment of humeral epicondylar fracture, medial or lateral; without manipulation
24565	Closed treatment of humeral epicondylar fracture, medial or lateral; with manipulation
24566	Percutaneous skeletal fixation of humeral epicondylar fracture, medial or lateral, with manipulation
24575	Open treatment of humeral epicondylar fracture, medial or lateral, includes internal fixation, when performed
24576	Closed treatment of humeral condylar fracture, medial or lateral; without manipulation
24577	Closed treatment of humeral condylar fracture, medial or lateral; with manipulation

24579	Open treatment of humeral condylar fracture, medial or lateral, includes internal fixation, when performed
24582	Percutaneous skeletal fixation of humeral condylar fracture, medial or lateral, with manipulation
24586	Open treatment of periarticular fracture and/or dislocation of the elbow (fracture distal humerus and proximal ulna and/or proximal radius);
24587	Open treatment of periarticular fracture and/or dislocation of the elbow (fracture distal humerus and proximal ulna and/or proximal radius); with implant arthroplasty
24620	Closed treatment of Monteggia type of fracture dislocation at elbow (fracture proximal end of ulna with dislocation of radial head), with manipulation
24635	Open treatment of Monteggia type of fracture dislocation at elbow (fracture proximal end of ulna with dislocation of radial head), includes internal fixation, when performed
24650	Closed treatment of radial head or neck fracture; without manipulation
24655	Closed treatment of radial head or neck fracture; with manipulation
24665	Open treatment of radial head or neck fracture, includes internal fixation or radial head excision, when performed;
24666	Open treatment of radial head or neck fracture, includes internal fixation or radial head excision, when performed; with radial head prosthetic replacement
24670	Closed treatment of ulnar fracture, proximal end (eg, olecranon or coronoid process[es]); without manipulation
24675	Closed treatment of ulnar fracture, proximal end (eg, olecranon or coronoid process[es]); with manipulation
24685	Open treatment of ulnar fracture, proximal end (eg, olecranon or coronoid process[es]), includes internal fixation, when performed
25500	Closed treatment of radial shaft fracture; without manipulation
25505	Closed treatment of radial shaft fracture; with manipulation
25515	Open treatment of radial shaft fracture, includes internal fixation, when performed
25520	Closed treatment of radial shaft fracture and closed treatment of dislocation of distal radioulnar joint (Galeazzi fracture/dislocation)
25525	Open treatment of radial shaft fracture, includes internal fixation, when performed, and closed treatment of distal radioulnar joint dislocation (Galeazzi fracture/ dislocation), includes percutaneous skeletal fixation, when performed
25526	Open treatment of radial shaft fracture, includes internal fixation, when performed, and open treatment of distal radioulnar joint dislocation (Galeazzi fracture/ dislocation), includes internal fixation, when performed, includes repair of triangular fibrocartilage complex
25530	Closed treatment of ulnar shaft fracture; without manipulation
25535	Closed treatment of ulnar shaft fracture; with manipulation
25545	Open treatment of ulnar shaft fracture, includes internal fixation, when performed
25560	Closed treatment of radial and ulnar shaft fractures; without manipulation
25565	Closed treatment of radial and ulnar shaft fractures; with manipulation
25574	Open treatment of radial AND ulnar shaft fractures, with internal fixation, when performed; of radius OR ulna
25575	Open treatment of radial AND ulnar shaft fractures, with internal fixation, when performed; of radius AND ulna
25600	Closed treatment of distal radial fracture (eg, Colles or Smith type) or epiphyseal separation, includes closed treatment of fracture of ulnar styloid, when performed; without manipulation
25605	Closed treatment of distal radial fracture (eg, Colles or Smith type) or epiphyseal separation, includes closed treatment of fracture of ulnar styloid, when performed; with manipulation
25606	Percutaneous skeletal fixation of distal radial fracture or epiphyseal separation
25607	Open treatment of distal radial extra-articular fracture or epiphyseal separation, with internal fixation

25608	Open treatment of distal radial intra-articular fracture or epiphyseal separation; with internal fixation of 2 fragments
25609	Open treatment of distal radial intra-articular fracture or epiphyseal separation; with internal fixation of 3 or more fragments
25611	Percutaneous skeletal fixation of distal radial fracture (eg, Colles or Smith type) or epiphyseal separation, with or without fracture of ulnar styloid, requiring manipulation, with or without external fixation
25620	Open treatment of distal radial fracture (eg, Colles or Smith type) or epiphyseal separation, with or without fracture of ulnar styloid, with or without internal or external fixation
25650	Closed treatment of ulnar styloid fracture
25651	Percutaneous skeletal fixation of ulnar styloid fracture
25652	Open treatment of ulnar styloid fracture
27125	Partial hip replacement
27200	Treat tail bone fracture
27202	Treat tail bone fracture
27220	Treat hip socket fracture
27222	Treat hip socket fracture
27226	Treat hip wall fracture
27227	Treat hip fracture(s)
27228	Treat hip fracture(s)
27230	Treat thigh fracture
27232	Treat thigh fracture
27235	Treat thigh fracture
27236	Treat thigh fracture
27238	Treat thigh fracture
27240	Treat thigh fracture
27244	Treat thigh fracture
27245	Treat thigh fracture
27246	Treat thigh fracture
27248	Treat thigh fracture
27254	Treat hip dislocation
27267	Closed treatment of femoral fracture, proximal end, head; without manipulation
27268	Closed treatment of femoral fracture, proximal end, head; with manipulation
27269	Open treatment of femoral fracture, proximal end, head, includes internal fixation, when performed
27500	Closed treatment of femoral shaft fracture, without manipulation
27500	Treatment of thigh fracture
27501	Closed treatment of supracondylar or transcondylar femoral fracture with or without intercondylar extension, without manipulation
27501	Treatment of thigh fracture
27502	Closed treatment of femoral shaft fracture, with manipulation, with or without skin or skeletal traction
27502	Treatment of thigh fracture
27503	Closed treatment of supracondylar or transcondylar femoral fracture with or without intercondylar extension, with manipulation, with or without skin or skeletal traction
27503	Treatment of thigh fracture
27506	Open treatment of femoral shaft fracture, with or without external fixation, with insertion of intramedullary implant, with or without cerclage and/or locking screws

27506	Treatment of thigh fracture
27507	Open treatment of femoral shaft fracture with plate/screws, with or without cerclage
27507	Treatment of thigh fracture
27508	Closed treatment of femoral fracture, distal end, medial or lateral condyle, without manipulation
27508	Treatment of thigh fracture
27509	Percutaneous skeletal fixation of femoral fracture, distal end, medial or lateral condyle, or supracondylar or transcondylar, with or without intercondylar extension, or distal femoral epiphyseal separation
27509	Treatment of thigh fracture
27510	Closed treatment of femoral fracture, distal end, medial or lateral condyle, with manipulation
27510	Treatment of thigh fracture
27511	Open treatment of femoral supracondylar or transcondylar fracture without intercondylar extension, includes internal fixation, when performed
27511	Treatment of thigh fracture
27513	Open treatment of femoral supracondylar or transcondylar fracture with intercondylar extension, includes internal fixation, when performed
27513	Treatment of thigh fracture
27514	Open treatment of femoral fracture, distal end, medial or lateral condyle, includes internal fixation, when performed
27514	Treatment of thigh fracture
27530	Treat knee fracture
27532	Treat knee fracture
27535	Treat knee fracture
27550	Treat knee dislocation
27552	Treat knee dislocation
27556	Treat knee dislocation
27557	Treat knee dislocation
27558	Treat knee dislocation
27759	Treatment of tibia fracture
27760	Closed treatment of medial malleolus fracture; without manipulation
27762	Closed treatment of medial malleolus fracture; with manipulation, with or without skin or skeletal traction
27766	Open treatment of medial malleolus fracture, includes internal fixation, when performed
27767	Closed treatment of posterior malleolus fracture; without manipulation
27768	Closed treatment of posterior malleolus fracture; with manipulation
27769	Open treatment of posterior malleolus fracture, includes internal fixation, when performed
27780	Treatment of fibula fracture
27781	Treatment of fibula fracture
27784	Treatment of fibula fracture
27786	Closed treatment of distal fibular fracture (lateral malleolus); without manipulation
27788	Closed treatment of distal fibular fracture (lateral malleolus); with manipulation
27792	Open treatment of distal fibular fracture (lateral malleolus), includes internal fixation, when performed
27808	Closed treatment of bimalleolar ankle fracture (eg, lateral and medial malleoli, or lateral and posterior malleoli or medial and posterior malleoli); without manipulation
27810	Closed treatment of bimalleolar ankle fracture (eg, lateral and medial malleoli, or lateral and posterior malleoli or medial and posterior malleoli); with manipulation

27814	Open treatment of bimalleolar ankle fracture (eg, lateral and medial malleoli, or lateral and posterior malleoli, or medial and posterior malleoli), includes internal fixation, when performed
27816	Closed treatment of trimalleolar ankle fracture; without manipulation
27818	Closed treatment of trimalleolar ankle fracture; with manipulation
27822	Open treatment of trimalleolar ankle fracture, includes internal fixation, when performed, medial and/or lateral malleolus; without fixation of posterior lip
27823	Open treatment of trimalleolar ankle fracture, includes internal fixation, when performed, medial and/or lateral malleolus; with fixation of posterior lip
27824	Treat lower leg fracture
27825	Treat lower leg fracture
27826	Treat lower leg fracture
27827	Treat lower leg fracture
27828	Treat lower leg fracture
28430	Closed treatment of talus fracture; without manipulation
28435	Closed treatment of talus fracture; with manipulation
28436	Percutaneous skeletal fixation of talus fracture, with manipulation
28445	Open treatment of talus fracture, includes internal fixation, when performed
72291	Perq verte/sacroplsty fluor
72292	Perq verte/sacroplsty ct
73311	Pathologic fracture of humerus
73312	Pathologic fracture of distal radius and ulna
73313	Pathologic fracture of vertebrae
73315	Pathologic fracture of other specified part of femur
73316	Pathologic fracture of tibia and fibula
77082	Dxa bone density vert fx
77085	Dxa bone density study
77086	Fracture assessment via dxa
80500	Closed fracture of cervical vertebra, unspecified level
80501	Closed fracture of first cervical vertebra
80502	Closed fracture of second cervical vertebra
80503	Closed fracture of third cervical vertebra
80504	Closed fracture of fourth cervical vertebra
80505	Closed fracture of fifth cervical vertebra
80506	Closed fracture of sixth cervical vertebra
80507	Closed fracture of seventh cervical vertebra
80508	Closed fracture of multiple cervical vertebrae
80620	Closed fracture of T1-T6 level with unspecified spinal cord injury
80621	Closed fracture of T1-T6 level with complete lesion of cord
80622	Closed fracture of T1-T6 level with anterior cord syndrome
80623	Closed fracture of T1-T6 level with central cord syndrome
80624	Closed fracture of T1-T6 level with other specified spinal cord injury
80625	Closed fracture of T7-T12 level with unspecified spinal cord injury
80626	Closed fracture of T7-T12 level with complete lesion of cord
80627	Closed fracture of T7-T12 level with anterior cord syndrome

80628	Closed fracture of T7-T12 level with central cord syndrome
80629	Closed fracture of T7-T12 level with other specified spinal cord injury
80630	Open fracture of T1-T6 level with unspecified spinal cord injury
80631	Open fracture of T1-T6 level with complete lesion of cord
80632	Open fracture of T1-T6 level with anterior cord syndrome
80633	Open fracture of T1-T6 level with central cord syndrome
80634	Open fracture of T1-T6 level with other specified spinal cord injury
80635	Open fracture of T7-T12 level with unspecified spinal cord injury
80636	Open fracture of T7-T12 level with complete lesion of cord
80637	Open fracture of T7-T12 level with anterior cord syndrome
80638	Open fracture of T7-T12 level with central cord syndrome
80639	Open fracture of T7-T12 level with other specified spinal cord injury
80660	Closed fracture of sacrum and coccyx with unspecified spinal cord injury
80661	Closed fracture of sacrum and coccyx with complete cauda equina lesion
80662	Closed fracture of sacrum and coccyx with other cauda equina injury
80669	Closed fracture of sacrum and coccyx with other spinal cord injury
80670	Open fracture of sacrum and coccyx with unspecified spinal cord injury
80671	Open fracture of sacrum and coccyx with complete cauda equina lesion
80672	Open fracture of sacrum and coccyx with other cauda equina injury
80679	Open fracture of sacrum and coccyx with other spinal cord injury
80841	Closed fracture of ilium
80842	Closed fracture of ischium
80843	Multiple closed pelvic fractures with disruption of pelvic circle
80844	Multiple closed pelvic fractures without disruption of pelvic circle
80849	Closed fracture of other specified part of pelvis
80851	Open fracture of ilium
80852	Open fracture of ischium
80853	Multiple open pelvic fractures with disruption of pelvic circle
80854	Multiple open pelvic fractures without disruption of pelvic circle
80859	Open fracture of other specified part of pelvis
81000	Unspecified part of closed fracture of clavicle
81001	Closed fracture of sternal end of clavicle
81002	Closed fracture of shaft of clavicle
81003	Closed fracture of acromial end of clavicle
81010	Unspecified part of open fracture of clavicle
81011	Open fracture of sternal end of clavicle
81012	Open fracture of shaft of clavicle
81013	Open fracture of acromial end of clavicle
81200	Closed fracture of unspecified part of upper end of humerus
81201	Closed fracture of surgical neck of humerus
81202	Closed fracture of anatomical neck of humerus
81203	Closed fracture of greater tuberosity of humerus
81209	Other closed fractures of upper end of humerus

81210	Open fracture of unspecified part of upper end of humerus
81211	Open fracture of surgical neck of humerus
81212	Open fracture of anatomical neck of humerus
81213	Open fracture of greater tuberosity of humerus
81219	Other open fracture of upper end of humerus
81220	Closed fracture of unspecified part of humerus
81221	Closed fracture of shaft of humerus
81230	Open fracture of unspecified part of humerus
81231	Open fracture of shaft of humerus
81240	Closed fracture of unspecified part of lower end of humerus
81241	Closed fracture of supracondylar humerus
81242	Closed fracture of lateral condyle of humerus
81243	Closed fracture of medial condyle of humerus
81244	Closed fracture of unspecified condyle(s) of humerus
81249	Other closed fracture of lower end of humerus
81250	Open fracture of unspecified part of lower end of humerus
81251	Open fracture of supracondylar humerus
81252	Open fracture of lateral condyle of humerus
81253	Open fracture of medial condyle of humerus
81254	Open fracture of unspecified condyle(s) of humerus
81259	Other open fracture of lower end of humerus
81300	Unspecified fracture of radius and ulna, upper end of forearm, closed
81301	Closed fracture of olecranon process of ulna
81302	Closed fracture of coronoid process of ulna
81303	Closed Monteggia's fracture
81304	Other and unspecified closed fractures of proximal end of ulna (alone)
81305	Closed fracture of head of radius
81306	Closed fracture of neck of radius
81307	Other and unspecified closed fractures of proximal end of radius (alone)
81308	Closed fracture of radius with ulna, upper end (any part)
81310	Unspecified open fracture of upper end of forearm
81311	Open fracture of olecranon process of ulna
81312	Open fracture of coronoid process of ulna
81313	Open Monteggia's fracture
81314	Other and unspecified open fractures of proximal end of ulna (alone)
81315	Open fracture of head of radius
81316	Open fracture of neck of radius
81317	Other and unspecified open fractures of proximal end of radius (alone)
81318	Open fracture of radius with ulna, upper end (any part)
81320	Unspecified closed fracture of shaft of radius or ulna
81321	Closed fracture of shaft of radius (alone)
81322	Closed fracture of shaft of ulna (alone)
81323	Closed fracture of shaft of radius with ulna

81330	Unspecified open fracture of shaft of radius or ulna
81331	Open fracture of shaft of radius (alone)
81332	Open fracture of shaft of ulna (alone)
81333	Open fracture of shaft of radius with ulna
81340	Unspecified closed fracture of lower end of forearm
81341	Closed Colles' fracture
81342	Other closed fractures of distal end of radius (alone)
81343	Closed fracture of distal end of ulna (alone)
81344	Closed fracture of lower end of radius with ulna
81345	Torus fracture of radius (alone)
81346	Torus fracture of ulna (alone)
81347	Torus fracture of radius and ulna
81350	Unspecified open fracture of lower end of forearm
81351	Open Colles' fracture
81352	Other open fractures of distal end of radius (alone)
81353	Open fracture of distal end of ulna (alone)
81354	Open fracture of lower end of radius with ulna
81380	Closed fracture of unspecified part of forearm
81381	Closed fracture of unspecified part of radius (alone)
81382	Closed fracture of unspecified part of ulna (alone)
81383	Closed fracture of unspecified part of radius with ulna
81390	Open fracture of unspecified part of forearm
81391	Open fracture of unspecified part of radius (alone)
81392	Open fracture of unspecified part of ulna (alone)
81393	Open fracture of unspecified part of radius with ulna
81406	Closed fracture of trapezoid bone (smaller multangular) of wrist
81407	Closed fracture of capitate bone (os magnum) of wrist
81408	Closed fracture of hamate (unciform) bone of wrist
81409	Closed fracture of other bone of wrist
81410	Unspecified open fracture of carpal bone
81411	Open fracture of navicular (scaphoid) bone of wrist
81412	Open fracture of lunate (semilunar) bone of wrist
81413	Open fracture of triquetral (cuneiform) bone of wrist
81414	Open fracture of pisiform bone of wrist
81415	Open fracture of trapezium bone (larger multangular) of wrist
81416	Open fracture of trapezoid bone (smaller multangular) of wrist
81417	Open fracture of capitate bone (os magnum) of wrist
81418	Open fracture of hamate (unciform) bone of wrist
81419	Open fracture of other bone of wrist
82000	Closed fracture of intracapsular section of neck of femur, unspecified
82001	Closed fracture of epiphysis (separation) (upper) of neck of femur
82002	Closed fracture of midcervical section of neck of femur
82003	Closed fracture of base of neck of femur

82009	Other closed transcervical fracture of neck of femur
82010	Open fracture of intracapsular section of neck of femur, unspecified
82011	Open fracture of epiphysis (separation) (upper) of neck of femur
82012	Open fracture of midcervical section of neck of femur
82013	Open fracture of base of neck of femur
82019	Other open transcervical fracture of neck of femur
82020	Closed fracture of trochanteric section of neck of femur
82021	Closed fracture of intertrochanteric section of neck of femur
82022	Closed fracture of subtrochanteric section of neck of femur
82030	Open fracture of trochanteric section of neck of femur, unspecified
82031	Open fracture of intertrochanteric section of neck of femur
82032	Open fracture of subtrochanteric section of neck of femur
82100	Closed fracture of unspecified part of femur
82101	Closed fracture of shaft of femur
82110	Open fracture of unspecified part of femur
82111	Open fracture of shaft of femur
82120	Closed fracture of unspecified part of lower end of femur
82121	Closed fracture of femoral condyle
82122	Closed fracture of epiphysis, lower (separation) of femur
82123	Closed supracondylar fracture of femur
82129	Other closed fracture of lower end of femur
82130	Open fracture of unspecified part of lower end of femur
82131	Open fracture of femoral condyle
82133	Open supracondylar fracture of femur
82139	Other open fracture of lower end of femur
82300	Closed fracture of upper end of tibia
82301	Closed fracture of upper end of fibula
82302	Closed fracture of upper end of fibula with tibia
82310	Open fracture of upper end of tibia
82311	Open fracture of upper end of fibula
82312	Open fracture of upper end of fibula with tibia
82320	Closed fracture of shaft of tibia
82321	Closed fracture of shaft of fibula
82322	Closed fracture of shaft of fibula with tibia
82330	Open fracture of shaft of tibia
82331	Open fracture of shaft of fibula
82332	Open fracture of shaft of fibula with tibia
82340	Torus fracture of tibia alone
82341	Torus fracture of fibula alone
82342	Torus fracture of fibula with tibia
82380	Closed fracture of unspecified part of tibia
82381	Closed fracture of unspecified part of fibula
82382	Closed fracture of unspecified part of fibula with tibia

82390	Open fracture of unspecified part of tibia
82391	Open fracture of unspecified part of fibula
82392	Open fracture of unspecified part of fibula with tibia
82521	Closed fracture of astragalus
82531	Open fracture of astragalus
0QB80ZZ	Excision of Right Femoral Shaft, Open Approach
0QBB0ZX	Excision of Right Lower Femur, Open Approach, Diagnostic
0QBB0ZZ	Excision of Right Lower Femur, Open Approach
0QBB3ZX	Excision of Right Lower Femur, Percutaneous Approach, Diagnostic
0QBB3ZZ	Excision of Right Lower Femur, Percutaneous Approach
0QBB4ZX	Excision of Right Lower Femur, Percutaneous Endoscopic Approach, Diagnostic
0QBB4ZZ	Excision of Right Lower Femur, Percutaneous Endoscopic Approach
0QBC0ZX	Excision of Left Lower Femur, Open Approach, Diagnostic
0QBC0ZZ	Excision of Left Lower Femur, Open Approach
0QBC3ZX	Excision of Left Lower Femur, Percutaneous Approach, Diagnostic
0QBC3ZZ	Excision of Left Lower Femur, Percutaneous Approach
0QBC4ZX	Excision of Left Lower Femur, Percutaneous Endoscopic Approach, Diagnostic
0QBC4ZZ	Excision of Left Lower Femur, Percutaneous Endoscopic Approach
0QH604Z	Insertion of Internal Fixation Device into Right Upper Femur, Open Approach
0QH605Z	Insertion of External Fixation Device into Right Upper Femur, Open Approach
0QH606Z	Insertion of Intramedullary Internal Fixation Device into Right Upper Femur, Open Approach
0QH60B Z	Insertion of Monoplanar External Fixation Device into Right Upper Femur, Open Approach
0QH60CZ	Insertion of Ring External Fixation Device into Right Upper Femur, Open Approach
0QH60D Z	Insertion of Hybrid External Fixation Device into Right Upper Femur, Open Approach
0QH634Z	Insertion of Internal Fixation Device into Right Upper Femur, Percutaneous Approach
0QH635Z	Insertion of External Fixation Device into Right Upper Femur, Percutaneous Approach
0QH636Z	Insertion of Intramedullary Internal Fixation Device into Right Upper Femur, Percutaneous Approach
0QH63B Z	Insertion of Monoplanar External Fixation Device into Right Upper Femur, Percutaneous Approach
0QH63CZ	Insertion of Ring External Fixation Device into Right Upper Femur, Percutaneous Approach
0QH63D Z	Insertion of Hybrid External Fixation Device into Right Upper Femur, Percutaneous Approach
0QH644Z	Insertion of Internal Fixation Device into Right Upper Femur, Percutaneous Endoscopic Approach
0QH645Z	Insertion of External Fixation Device into Right Upper Femur, Percutaneous Endoscopic Approach
0QH646Z	Insertion of Intramedullary Internal Fixation Device into Right Upper Femur, Percutaneous Endoscopic Approach
0QH64B Z	Insertion of Monoplanar External Fixation Device into Right Upper Femur, Percutaneous Endoscopic Approach
0QH64CZ	Insertion of Ring External Fixation Device into Right Upper Femur, Percutaneous Endoscopic Approach
0QH64D Z	Insertion of Hybrid External Fixation Device into Right Upper Femur, Percutaneous Endoscopic Approach
0QH704Z	Insertion of Internal Fixation Device into Left Upper Femur, Open Approach
0QH705Z	Insertion of External Fixation Device into Left Upper Femur, Open Approach

0QH734Z	Insertion of Internal Fixation Device into Left Upper Femur, Percutaneous Approach
0QH744Z	Insertion of Internal Fixation Device into Left Upper Femur, Percutaneous Endoscopic Approach
0QH745Z	Insertion of External Fixation Device into Left Upper Femur, Percutaneous Endoscopic Approach
0QH746Z	Insertion of Intramedullary Internal Fixation Device into Left Upper Femur, Percutaneous Endoscopic Approach
0QH74BZ	Insertion of Monoplanar External Fixation Device into Left Upper Femur, Percutaneous Endoscopic Approach
0QH74CZ	Insertion of Ring External Fixation Device into Left Upper Femur, Percutaneous Endoscopic Approach
0QH804Z	Insertion of Internal Fixation Device into Right Femoral Shaft, Open Approach
0QH834Z	Insertion of Internal Fixation Device into Right Femoral Shaft, Percutaneous Approach
0QH844Z	Insertion of Internal Fixation Device into Right Femoral Shaft, Percutaneous Endoscopic Approach
0QH904Z	Insertion of Internal Fixation Device into Left Femoral Shaft, Open Approach
0QH90ZZ	Excision of Left Femoral Shaft, Open Approach
0QH934Z	Insertion of Internal Fixation Device into Left Femoral Shaft, Percutaneous Approach
0QH944Z	Insertion of Internal Fixation Device into Left Femoral Shaft, Percutaneous Endoscopic Approach
0QHB04Z	Insertion of Internal Fixation Device into Right Lower Femur, Open Approach
0QHB05Z	Insertion of External Fixation Device into Right Lower Femur, Open Approach
0QHB06Z	Insertion of Intramedullary Internal Fixation Device into Right Lower Femur, Open Approach
0QHB0BZ	Insertion of Monoplanar External Fixation Device into Right Lower Femur, Open Approach
0QHB0CZ	Insertion of Ring External Fixation Device into Right Lower Femur, Open Approach
0QHB0DZ	Insertion of Hybrid External Fixation Device into Right Lower Femur, Open Approach
0QHB34Z	Insertion of Internal Fixation Device into Right Lower Femur, Percutaneous Approach
0QHB35Z	Insertion of External Fixation Device into Right Lower Femur, Percutaneous Approach
0QHB36Z	Insertion of Intramedullary Internal Fixation Device into Right Lower Femur, Percutaneous Approach
0QHB3BZ	Insertion of Monoplanar External Fixation Device into Right Lower Femur, Percutaneous Approach
0QHB3CZ	Insertion of Ring External Fixation Device into Right Lower Femur, Percutaneous Approach
0QHB3DZ	Insertion of Hybrid External Fixation Device into Right Lower Femur, Percutaneous Approach
0QHB44Z	Insertion of Internal Fixation Device into Right Lower Femur, Percutaneous Endoscopic Approach
0QHB45Z	Insertion of External Fixation Device into Right Lower Femur, Percutaneous Endoscopic Approach
0QHB46Z	Insertion of Intramedullary Internal Fixation Device into Right Lower Femur, Percutaneous Endoscopic Approach
0QHB4BZ	Insertion of Monoplanar External Fixation Device into Right Lower Femur, Percutaneous Endoscopic Approach
0QHB4CZ	Insertion of Ring External Fixation Device into Right Lower Femur, Percutaneous Endoscopic Approach

0QHB4D Z	Insertion of Hybrid External Fixation Device into Right Lower Femur, Percutaneous Endoscopic Approach
0QHC04Z	Insertion of Internal Fixation Device into Left Lower Femur, Open Approach
0QHC05Z	Insertion of External Fixation Device into Left Lower Femur, Open Approach
0QHC06Z	Insertion of Intramedullary Internal Fixation Device into Left Lower Femur, Open Approach
0QHC0B Z	Insertion of Monoplanar External Fixation Device into Left Lower Femur, Open Approach
0QHC0C Z	Insertion of Ring External Fixation Device into Left Lower Femur, Open Approach
0QHC0D Z	Insertion of Hybrid External Fixation Device into Left Lower Femur, Open Approach
0QHC34Z	Insertion of Internal Fixation Device into Left Lower Femur, Percutaneous Approach
0QHC35Z	Insertion of External Fixation Device into Left Lower Femur, Percutaneous Approach
0QHC36Z	Insertion of Intramedullary Internal Fixation Device into Left Lower Femur, Percutaneous Approach
0QHC3B Z	Insertion of Monoplanar External Fixation Device into Left Lower Femur, Percutaneous Approach
0QHC3C Z	Insertion of Ring External Fixation Device into Left Lower Femur, Percutaneous Approach
0QHC3D Z	Insertion of Hybrid External Fixation Device into Left Lower Femur, Percutaneous Approach
0QHC44Z	Insertion of Internal Fixation Device into Left Lower Femur, Percutaneous Endoscopic Approach
0QHC45Z	Insertion of External Fixation Device into Left Lower Femur, Percutaneous Endoscopic Approach
0QHC46Z	Insertion of Intramedullary Internal Fixation Device into Left Lower Femur, Percutaneous Endoscopic Approach
0QHC4B Z	Insertion of Monoplanar External Fixation Device into Left Lower Femur, Percutaneous Endoscopic Approach
0QHC4C Z	Insertion of Ring External Fixation Device into Left Lower Femur, Percutaneous Endoscopic Approach
0QHC4D Z	Insertion of Hybrid External Fixation Device into Left Lower Femur, Percutaneous Endoscopic Approach
0QS604Z	Reposition Right Upper Femur with Internal Fixation Device, Open Approach
0QS60ZZ	Reposition Right Upper Femur, Open Approach
0QS634Z	Reposition Right Upper Femur with Internal Fixation Device, Percutaneous Approach
0QS63ZZ	Reposition Right Upper Femur, Percutaneous Approach
0QS644Z	Reposition Right Upper Femur with Internal Fixation Device, Percutaneous Endoscopic Approach
0QS64ZZ	Reposition Right Upper Femur, Percutaneous Endoscopic Approach
0QS6XZZ	Reposition Right Upper Femur, External Approach
0QS704Z	Reposition Left Upper Femur with Internal Fixation Device, Open Approach
0QS70ZZ	Reposition Left Upper Femur, Open Approach
0QS734Z	Reposition Left Upper Femur with Internal Fixation Device, Percutaneous Approach
0QS73ZZ	Reposition Left Upper Femur, Percutaneous Approach
0QS744Z	Reposition Left Upper Femur with Internal Fixation Device, Percutaneous Endoscopic Approach
0QS74ZZ	Reposition Left Upper Femur, Percutaneous Endoscopic Approach
0QS7XZZ	Reposition Left Upper Femur, External Approach
0QS804Z	Reposition Right Femoral Shaft with Internal Fixation Device, Open Approach
0QS80ZZ	Reposition Right Femoral Shaft, Open Approach
0QS834Z	Reposition Right Femoral Shaft with Internal Fixation Device, Percutaneous Approach

0QS83ZZ	Reposition Right Femoral Shaft, Percutaneous Approach
0QS844Z	Reposition Right Femoral Shaft with Internal Fixation Device, Percutaneous Endoscopic Approach
0QS84ZZ	Reposition Right Femoral Shaft, Percutaneous Endoscopic Approach
0QS8XZZ	Reposition Right Femoral Shaft, External Approach
0QS904Z	Reposition Left Femoral Shaft with Internal Fixation Device, Open Approach
0QS90ZZ	Reposition Left Femoral Shaft, Open Approach
0QS934Z	Reposition Left Femoral Shaft with Internal Fixation Device, Percutaneous Approach
0QS93ZZ	Reposition Left Femoral Shaft, Percutaneous Approach
0QS944Z	Reposition Left Femoral Shaft with Internal Fixation Device, Percutaneous Endoscopic Approach
0QS94ZZ	Reposition Left Femoral Shaft, Percutaneous Endoscopic Approach
0QS9XZZ	Reposition Left Femoral Shaft, External Approach
0QSB04Z	Reposition Right Lower Femur with Internal Fixation Device, Open Approach
0QSB0ZZ	Reposition Right Lower Femur, Open Approach
0QSB34Z	Reposition Right Lower Femur with Internal Fixation Device, Percutaneous Approach
0QSB3ZZ	Reposition Right Lower Femur, Percutaneous Approach
0QSB44Z	Reposition Right Lower Femur with Internal Fixation Device, Percutaneous Endoscopic Approach
0QSB4ZZ	Reposition Right Lower Femur, Percutaneous Endoscopic Approach
0QSBXZZ	Reposition Right Lower Femur, External Approach
0QSC04Z	Reposition Left Lower Femur with Internal Fixation Device, Open Approach
0QSC0ZZ	Reposition Left Lower Femur, Open Approach
0QSC34Z	Reposition Left Lower Femur with Internal Fixation Device, Percutaneous Approach
0QSC3ZZ	Reposition Left Lower Femur, Percutaneous Approach
0QSC44Z	Reposition Left Lower Femur with Internal Fixation Device, Percutaneous Endoscopic Approach
0QSC4ZZ	Reposition Left Lower Femur, Percutaneous Endoscopic Approach
0QSCXZZ	Reposition Left Lower Femur, External Approach
M4844XA	Fatigue fracture of vertebra, thoracic region, initial encounter for fracture
M4845XA	Fatigue fracture of vertebra, thoracolumbar region, initial encounter for fracture
M4846XA	Fatigue fracture of vertebra, lumbar region, initial encounter for fracture
M4847XA	Fatigue fracture of vertebra, lumbosacral region, initial encounter for fracture
M4848XA	Fatigue fracture of vertebra, sacral and sacrococcygeal region, initial encounter for fracture
M4850XA	Collapsed vertebra, not elsewhere classified, site unspecified, initial encounter for fracture
M4851XA	Collapsed vertebra, not elsewhere classified, occipito-atlanto-axial region, initial encounter for fracture
M4852XA	Collapsed vertebra, not elsewhere classified, cervical region, initial encounter for fracture
M4853XA	Collapsed vertebra, not elsewhere classified, cervicothoracic region, initial encounter for fracture
M4854XA	Collapsed vertebra, not elsewhere classified, thoracic region, initial encounter for fracture
M4855XA	Collapsed vertebra, not elsewhere classified, thoracolumbar region, initial encounter for fracture

M4856X A	Collapsed vertebra, not elsewhere classified, lumbar region, initial encounter for fracture
M4857X A	Collapsed vertebra, not elsewhere classified, lumbosacral region, initial encounter for fracture
M4858X A	Collapsed vertebra, not elsewhere classified, sacral and sacrococcygeal region, initial encounter for fracture
M80011 A	Age-related osteoporosis with current pathological fracture, right shoulder, initial encounter for fracture
M80012 A	Age-related osteoporosis with current pathological fracture, left shoulder, initial encounter for fracture
M80019 A	Age-related osteoporosis with current pathological fracture, unspecified shoulder, initial encounter for fracture
M80021 A	Age-related osteoporosis with current pathological fracture, right humerus, initial encounter for fracture
M80022 A	Age-related osteoporosis with current pathological fracture, left humerus, initial encounter for fracture
M80029 A	Age-related osteoporosis with current pathological fracture, unspecified humerus, initial encounter for fracture
M80031 A	Age-related osteoporosis with current pathological fracture, right forearm, initial encounter for fracture
M80032 A	Age-related osteoporosis with current pathological fracture, left forearm, initial encounter for fracture
M80039 A	Age-related osteoporosis with current pathological fracture, unspecified forearm, initial encounter for fracture
M80051 A	Age-related osteoporosis with current pathological fracture, right femur, initial encounter for fracture
M80052 A	Age-related osteoporosis with current pathological fracture, left femur, initial encounter for fracture
M80059 A	Age-related osteoporosis with current pathological fracture, unspecified femur, initial encounter for fracture
M80061 A	Age-related osteoporosis with current pathological fracture, right lower leg, initial encounter for fracture
M80062 A	Age-related osteoporosis with current pathological fracture, left lower leg, initial encounter for fracture
M80069 A	Age-related osteoporosis with current pathological fracture, unspecified lower leg, initial encounter for fracture
M80071 A	Age-related osteoporosis with current pathological fracture, right ankle and foot, initial encounter for fracture
M80072 A	Age-related osteoporosis with current pathological fracture, left ankle and foot, initial encounter for fracture
M80079 A	Age-related osteoporosis with current pathological fracture, unspecified ankle and foot, initial encounter for fracture
M8008X A	Age-related osteoporosis with current pathological fracture, vertebra(e), initial encounter for fracture
M80811 A	Other osteoporosis with current pathological fracture, right shoulder, initial encounter for fracture
M80812 A	Other osteoporosis with current pathological fracture, left shoulder, initial encounter for fracture
M80819 A	Other osteoporosis with current pathological fracture, unspecified shoulder, initial encounter for fracture
M80821	Other osteoporosis with current pathological fracture, right humerus, initial encounter for fracture

A	
M80822A	Other osteoporosis with current pathological fracture, left humerus, initial encounter for fracture
M80829A	Other osteoporosis with current pathological fracture, unspecified humerus, initial encounter for fracture
M80831A	Other osteoporosis with current pathological fracture, right forearm, initial encounter for fracture
M80832A	Other osteoporosis with current pathological fracture, left forearm, initial encounter for fracture
M80839A	Other osteoporosis with current pathological fracture, unspecified forearm, initial encounter for fracture
M80851A	Other osteoporosis with current pathological fracture, right femur, initial encounter for fracture
M80852A	Other osteoporosis with current pathological fracture, left femur, initial encounter for fracture
M80859A	Other osteoporosis with current pathological fracture, unspecified femur, initial encounter for fracture
M80861A	Other osteoporosis with current pathological fracture, right lower leg, initial encounter for fracture
M80862A	Other osteoporosis with current pathological fracture, left lower leg, initial encounter for fracture
M80869A	Other osteoporosis with current pathological fracture, unspecified lower leg, initial encounter for fracture
M80871A	Other osteoporosis with current pathological fracture, right ankle and foot, initial encounter for fracture
M80872A	Other osteoporosis with current pathological fracture, left ankle and foot, initial encounter for fracture
M80879A	Other osteoporosis with current pathological fracture, unspecified ankle and foot, initial encounter for fracture
M8088XA	Other osteoporosis with current pathological fracture, vertebra(e), initial encounter for fracture
M84411A	Pathological fracture, right shoulder, initial encounter for fracture
M84412A	Pathological fracture, left shoulder, initial encounter for fracture
M84419A	Pathological fracture, unspecified shoulder, initial encounter for fracture
M84421A	Pathological fracture, right humerus, initial encounter for fracture
M84422A	Pathological fracture, left humerus, initial encounter for fracture
M84429A	Pathological fracture, unspecified humerus, initial encounter for fracture
M84431A	Pathological fracture, right ulna, initial encounter for fracture
M84432A	Pathological fracture, left ulna, initial encounter for fracture
M84433A	Pathological fracture, right radius, initial encounter for fracture
M84434A	Pathological fracture, left radius, initial encounter for fracture

M84439 A	Pathological fracture, unspecified ulna and radius, initial encounter for fracture
M84451 A	Pathological fracture, right femur, initial encounter for fracture
M84452 A	Pathological fracture, left femur, initial encounter for fracture
M84453 A	Pathological fracture, unspecified femur, initial encounter for fracture
M84461 A	Pathological fracture, right tibia, initial encounter for fracture
M84462 A	Pathological fracture, left tibia, initial encounter for fracture
M84463 A	Pathological fracture, right fibula, initial encounter for fracture
M84464 A	Pathological fracture, left fibula, initial encounter for fracture
M84469 A	Pathological fracture, unspecified tibia and fibula, initial encounter for fracture
M84471 A	Pathological fracture, right ankle, initial encounter for fracture
M84472 A	Pathological fracture, left ankle, initial encounter for fracture
M84473 A	Pathological fracture, unspecified ankle, initial encounter for fracture
M8448X A	Pathological fracture, other site, initial encounter for fracture
M84521 A	Pathological fracture in neoplastic disease, right humerus, initial encounter for fracture
M84522 A	Pathological fracture in neoplastic disease, left humerus, initial encounter for fracture
M84529 A	Pathological fracture in neoplastic disease, unspecified humerus, initial encounter for fracture
M84531 A	Pathological fracture in neoplastic disease, right ulna, initial encounter for fracture
M84532 A	Pathological fracture in neoplastic disease, left ulna, initial encounter for fracture
M84533 A	Pathological fracture in neoplastic disease, right radius, initial encounter for fracture
M84534 A	Pathological fracture in neoplastic disease, left radius, initial encounter for fracture
M84539 A	Pathological fracture in neoplastic disease, unspecified ulna and radius, initial encounter for fracture
M8458X A	Pathological fracture in neoplastic disease, other specified site, initial encounter for fracture
M84611 A	Pathological fracture in other disease, right shoulder, initial encounter for fracture
M84612 A	Pathological fracture in other disease, left shoulder, initial encounter for fracture
M84619 A	Pathological fracture in other disease, unspecified shoulder, initial encounter for fracture
M84621	Pathological fracture in other disease, right humerus, initial encounter for fracture

A	
M84622 A	Pathological fracture in other disease, left humerus, initial encounter for fracture
M84629 A	Pathological fracture in other disease, unspecified humerus, initial encounter for fracture
M84631 A	Pathological fracture in other disease, right ulna, initial encounter for fracture
M84632 A	Pathological fracture in other disease, left ulna, initial encounter for fracture
M84633 A	Pathological fracture in other disease, right radius, initial encounter for fracture
M84634 A	Pathological fracture in other disease, left radius, initial encounter for fracture
M84639 A	Pathological fracture in other disease, unspecified ulna and radius, initial encounter for fracture
M84651 A	Pathological fracture in other disease, right femur, initial encounter for fracture
M84652 A	Pathological fracture in other disease, left femur, initial encounter for fracture
M84653 A	Pathological fracture in other disease, unspecified femur, initial encounter for fracture
M84661 A	Pathological fracture in other disease, right tibia, initial encounter for fracture
M84662 A	Pathological fracture in other disease, left tibia, initial encounter for fracture
M84663 A	Pathological fracture in other disease, right fibula, initial encounter for fracture
M84664 A	Pathological fracture in other disease, left fibula, initial encounter for fracture
M84669 A	Pathological fracture in other disease, unspecified tibia and fibula, initial encounter for fracture
M84671 A	Pathological fracture in other disease, right ankle, initial encounter for fracture
M84672 A	Pathological fracture in other disease, left ankle, initial encounter for fracture
M84673 A	Pathological fracture in other disease, unspecified ankle, initial encounter for fracture
M8468X A	Pathological fracture in other disease, other site, initial encounter for fracture
M84750 A	Atypical femoral fracture, unspecified, initial encounter for fracture
M84751 A	Incomplete atypical femoral fracture, right leg, initial encounter for fracture
M84752 A	Incomplete atypical femoral fracture, left leg, initial encounter for fracture
M84753 A	Incomplete atypical femoral fracture, unspecified leg, initial encounter for fracture
M84754 A	Complete transverse atypical femoral fracture, right leg, initial encounter for fracture
M84755 A	Complete transverse atypical femoral fracture, left leg, initial encounter for fracture

M84756<br>A	Complete transverse atypical femoral fracture, unspecified leg, initial encounter for fracture
M84757<br>A	Complete oblique atypical femoral fracture, right leg, initial encounter for fracture
M84758<br>A	Complete oblique atypical femoral fracture, left leg, initial encounter for fracture
M84759<br>A	Complete oblique atypical femoral fracture, unspecified leg, initial encounter for fracture
M9701X<br>A	Periprosthetic fracture around internal prosthetic right hip joint, initial encounter
M9702X<br>A	Periprosthetic fracture around internal prosthetic left hip joint, initial encounter
S12000A	Unspecified displaced fracture of first cervical vertebra, initial encounter for closed fracture
S12000B	Unspecified displaced fracture of first cervical vertebra, initial encounter for open fracture
S12001A	Unspecified nondisplaced fracture of first cervical vertebra, initial encounter for closed fracture
S12001B	Unspecified nondisplaced fracture of first cervical vertebra, initial encounter for open fracture
S1201XA	Stable burst fracture of first cervical vertebra, initial encounter for closed fracture
S1201XB	Stable burst fracture of first cervical vertebra, initial encounter for open fracture
S1202XA	Unstable burst fracture of first cervical vertebra, initial encounter for closed fracture
S1202XB	Unstable burst fracture of first cervical vertebra, initial encounter for open fracture
S12030A	Displaced posterior arch fracture of first cervical vertebra, initial encounter for closed fracture
S12030B	Displaced posterior arch fracture of first cervical vertebra, initial encounter for open fracture
S12031A	Nondisplaced posterior arch fracture of first cervical vertebra, initial encounter for closed fracture
S12031B	Nondisplaced posterior arch fracture of first cervical vertebra, initial encounter for open fracture
S12040A	Displaced lateral mass fracture of first cervical vertebra, initial encounter for closed fracture
S12040B	Displaced lateral mass fracture of first cervical vertebra, initial encounter for open fracture
S12041A	Nondisplaced lateral mass fracture of first cervical vertebra, initial encounter for closed fracture
S12041B	Nondisplaced lateral mass fracture of first cervical vertebra, initial encounter for open fracture
S12090A	Other displaced fracture of first cervical vertebra, initial encounter for closed fracture
S12090B	Other displaced fracture of first cervical vertebra, initial encounter for open fracture
S12091A	Other nondisplaced fracture of first cervical vertebra, initial encounter for closed fracture
S12091B	Other nondisplaced fracture of first cervical vertebra, initial encounter for open fracture
S12100A	Unspecified displaced fracture of second cervical vertebra, initial encounter for closed fracture
S12100B	Unspecified displaced fracture of second cervical vertebra, initial encounter for open fracture
S12101A	Unspecified nondisplaced fracture of second cervical vertebra, initial encounter for closed fracture
S12101B	Unspecified nondisplaced fracture of second cervical vertebra, initial encounter for open fracture
S12110A	Anterior displaced Type II dens fracture, initial encounter for closed fracture
S12110B	Anterior displaced Type II dens fracture, initial encounter for open fracture
S12111A	Posterior displaced Type II dens fracture, initial encounter for closed fracture
S12111B	Posterior displaced Type II dens fracture, initial encounter for open fracture
S12112A	Nondisplaced Type II dens fracture, initial encounter for closed fracture
S12112B	Nondisplaced Type II dens fracture, initial encounter for open fracture
S12120A	Other displaced dens fracture, initial encounter for closed fracture
S12120B	Other displaced dens fracture, initial encounter for open fracture
S12121A	Other nondisplaced dens fracture, initial encounter for closed fracture

S12121B	Other nondisplaced dens fracture, initial encounter for open fracture
S12130A	Unspecified traumatic displaced spondylolisthesis of second cervical vertebra, initial encounter for closed fracture
S12130B	Unspecified traumatic displaced spondylolisthesis of second cervical vertebra, initial encounter for open fracture
S12131A	Unspecified traumatic nondisplaced spondylolisthesis of second cervical vertebra, initial encounter for closed fracture
S12131B	Unspecified traumatic nondisplaced spondylolisthesis of second cervical vertebra, initial encounter for open fracture
S1214XA	Type III traumatic spondylolisthesis of second cervical vertebra, initial encounter for closed fracture
S1214XB	Type III traumatic spondylolisthesis of second cervical vertebra, initial encounter for open fracture
S12150A	Other traumatic displaced spondylolisthesis of second cervical vertebra, initial encounter for closed fracture
S12150B	Other traumatic displaced spondylolisthesis of second cervical vertebra, initial encounter for open fracture
S12151A	Other traumatic nondisplaced spondylolisthesis of second cervical vertebra, initial encounter for closed fracture
S12151B	Other traumatic nondisplaced spondylolisthesis of second cervical vertebra, initial encounter for open fracture
S12190A	Other displaced fracture of second cervical vertebra, initial encounter for closed fracture
S12190B	Other displaced fracture of second cervical vertebra, initial encounter for open fracture
S12191A	Other nondisplaced fracture of second cervical vertebra, initial encounter for closed fracture
S12191B	Other nondisplaced fracture of second cervical vertebra, initial encounter for open fracture
S12200A	Unspecified displaced fracture of third cervical vertebra, initial encounter for closed fracture
S12200B	Unspecified displaced fracture of third cervical vertebra, initial encounter for open fracture
S12201A	Unspecified nondisplaced fracture of third cervical vertebra, initial encounter for closed fracture
S12201B	Unspecified nondisplaced fracture of third cervical vertebra, initial encounter for open fracture
S12230A	Unspecified traumatic displaced spondylolisthesis of third cervical vertebra, initial encounter for closed fracture
S12230B	Unspecified traumatic displaced spondylolisthesis of third cervical vertebra, initial encounter for open fracture
S12231A	Unspecified traumatic nondisplaced spondylolisthesis of third cervical vertebra, initial encounter for closed fracture
S12231B	Unspecified traumatic nondisplaced spondylolisthesis of third cervical vertebra, initial encounter for open fracture
S1224XA	Type III traumatic spondylolisthesis of third cervical vertebra, initial encounter for closed fracture
S1224XB	Type III traumatic spondylolisthesis of third cervical vertebra, initial encounter for open fracture
S12250A	Other traumatic displaced spondylolisthesis of third cervical vertebra, initial encounter for closed fracture
S12250B	Other traumatic displaced spondylolisthesis of third cervical vertebra, initial encounter for open fracture
S12251A	Other traumatic nondisplaced spondylolisthesis of third cervical vertebra, initial encounter for closed fracture
S12251B	Other traumatic nondisplaced spondylolisthesis of third cervical vertebra, initial encounter for open fracture
S12290A	Other displaced fracture of third cervical vertebra, initial encounter for closed fracture
S12290B	Other displaced fracture of third cervical vertebra, initial encounter for open fracture
S12291A	Other nondisplaced fracture of third cervical vertebra, initial encounter for closed fracture

S12291B	Other nondisplaced fracture of third cervical vertebra, initial encounter for open fracture
S12300A	Unspecified displaced fracture of fourth cervical vertebra, initial encounter for closed fracture
S12300B	Unspecified displaced fracture of fourth cervical vertebra, initial encounter for open fracture
S12301A	Unspecified nondisplaced fracture of fourth cervical vertebra, initial encounter for closed fracture
S12301B	Unspecified nondisplaced fracture of fourth cervical vertebra, initial encounter for open fracture
S12330A	Unspecified traumatic displaced spondylolisthesis of fourth cervical vertebra, initial encounter for closed fracture
S12330B	Unspecified traumatic displaced spondylolisthesis of fourth cervical vertebra, initial encounter for open fracture
S12331A	Unspecified traumatic nondisplaced spondylolisthesis of fourth cervical vertebra, initial encounter for closed fracture
S12331B	Unspecified traumatic nondisplaced spondylolisthesis of fourth cervical vertebra, initial encounter for open fracture
S1234XA	Type III traumatic spondylolisthesis of fourth cervical vertebra, initial encounter for closed fracture
S1234XB	Type III traumatic spondylolisthesis of fourth cervical vertebra, initial encounter for open fracture
S12350A	Other traumatic displaced spondylolisthesis of fourth cervical vertebra, initial encounter for closed fracture
S12350B	Other traumatic displaced spondylolisthesis of fourth cervical vertebra, initial encounter for open fracture
S12351A	Other traumatic nondisplaced spondylolisthesis of fourth cervical vertebra, initial encounter for closed fracture
S12351B	Other traumatic nondisplaced spondylolisthesis of fourth cervical vertebra, initial encounter for open fracture
S12390A	Other displaced fracture of fourth cervical vertebra, initial encounter for closed fracture
S12390B	Other displaced fracture of fourth cervical vertebra, initial encounter for open fracture
S12391A	Other nondisplaced fracture of fourth cervical vertebra, initial encounter for closed fracture
S12391B	Other nondisplaced fracture of fourth cervical vertebra, initial encounter for open fracture
S12400A	Unspecified displaced fracture of fifth cervical vertebra, initial encounter for closed fracture
S12400B	Unspecified displaced fracture of fifth cervical vertebra, initial encounter for open fracture
S12401A	Unspecified nondisplaced fracture of fifth cervical vertebra, initial encounter for closed fracture
S12401B	Unspecified nondisplaced fracture of fifth cervical vertebra, initial encounter for open fracture
S12430A	Unspecified traumatic displaced spondylolisthesis of fifth cervical vertebra, initial encounter for closed fracture
S12430B	Unspecified traumatic displaced spondylolisthesis of fifth cervical vertebra, initial encounter for open fracture
S12431A	Unspecified traumatic nondisplaced spondylolisthesis of fifth cervical vertebra, initial encounter for closed fracture
S12431B	Unspecified traumatic nondisplaced spondylolisthesis of fifth cervical vertebra, initial encounter for open fracture
S1244XA	Type III traumatic spondylolisthesis of fifth cervical vertebra, initial encounter for closed fracture
S1244XB	Type III traumatic spondylolisthesis of fifth cervical vertebra, initial encounter for open fracture
S12450A	Other traumatic displaced spondylolisthesis of fifth cervical vertebra, initial encounter for closed fracture
S12450B	Other traumatic displaced spondylolisthesis of fifth cervical vertebra, initial encounter for open fracture
S12451A	Other traumatic nondisplaced spondylolisthesis of fifth cervical vertebra, initial encounter for closed fracture
S12451B	Other traumatic nondisplaced spondylolisthesis of fifth cervical vertebra, initial encounter for open

	fracture
S12490A	Other displaced fracture of fifth cervical vertebra, initial encounter for closed fracture
S12490B	Other displaced fracture of fifth cervical vertebra, initial encounter for open fracture
S12491A	Other nondisplaced fracture of fifth cervical vertebra, initial encounter for closed fracture
S12491B	Other nondisplaced fracture of fifth cervical vertebra, initial encounter for open fracture
S12500A	Unspecified displaced fracture of sixth cervical vertebra, initial encounter for closed fracture
S12500B	Unspecified displaced fracture of sixth cervical vertebra, initial encounter for open fracture
S12501A	Unspecified nondisplaced fracture of sixth cervical vertebra, initial encounter for closed fracture
S12501B	Unspecified nondisplaced fracture of sixth cervical vertebra, initial encounter for open fracture
S12530A	Unspecified traumatic displaced spondylolisthesis of sixth cervical vertebra, initial encounter for closed fracture
S12530B	Unspecified traumatic displaced spondylolisthesis of sixth cervical vertebra, initial encounter for open fracture
S12531A	Unspecified traumatic nondisplaced spondylolisthesis of sixth cervical vertebra, initial encounter for closed fracture
S12531B	Unspecified traumatic nondisplaced spondylolisthesis of sixth cervical vertebra, initial encounter for open fracture
S1254XA	Type III traumatic spondylolisthesis of sixth cervical vertebra, initial encounter for closed fracture
S1254XB	Type III traumatic spondylolisthesis of sixth cervical vertebra, initial encounter for open fracture
S12550A	Other traumatic displaced spondylolisthesis of sixth cervical vertebra, initial encounter for closed fracture
S12550B	Other traumatic displaced spondylolisthesis of sixth cervical vertebra, initial encounter for open fracture
S12551A	Other traumatic nondisplaced spondylolisthesis of sixth cervical vertebra, initial encounter for closed fracture
S12551B	Other traumatic nondisplaced spondylolisthesis of sixth cervical vertebra, initial encounter for open fracture
S12590A	Other displaced fracture of sixth cervical vertebra, initial encounter for closed fracture
S12590B	Other displaced fracture of sixth cervical vertebra, initial encounter for open fracture
S12591A	Other nondisplaced fracture of sixth cervical vertebra, initial encounter for closed fracture
S12591B	Other nondisplaced fracture of sixth cervical vertebra, initial encounter for open fracture
S12600A	Unspecified displaced fracture of seventh cervical vertebra, initial encounter for closed fracture
S12600B	Unspecified displaced fracture of seventh cervical vertebra, initial encounter for open fracture
S12601A	Unspecified nondisplaced fracture of seventh cervical vertebra, initial encounter for closed fracture
S12601B	Unspecified nondisplaced fracture of seventh cervical vertebra, initial encounter for open fracture
S12630A	Unspecified traumatic displaced spondylolisthesis of seventh cervical vertebra, initial encounter for closed fracture
S12630B	Unspecified traumatic displaced spondylolisthesis of seventh cervical vertebra, initial encounter for open fracture
S12631A	Unspecified traumatic nondisplaced spondylolisthesis of seventh cervical vertebra, initial encounter for closed fracture
S12631B	Unspecified traumatic nondisplaced spondylolisthesis of seventh cervical vertebra, initial encounter for open fracture
S1264XA	Type III traumatic spondylolisthesis of seventh cervical vertebra, initial encounter for closed fracture
S1264XB	Type III traumatic spondylolisthesis of seventh cervical vertebra, initial encounter for open fracture
S12650A	Other traumatic displaced spondylolisthesis of seventh cervical vertebra, initial encounter for closed fracture

S12650B	Other traumatic displaced spondylolisthesis of seventh cervical vertebra, initial encounter for open fracture
S12651A	Other traumatic nondisplaced spondylolisthesis of seventh cervical vertebra, initial encounter for closed fracture
S12651B	Other traumatic nondisplaced spondylolisthesis of seventh cervical vertebra, initial encounter for open fracture
S12690A	Other displaced fracture of seventh cervical vertebra, initial encounter for closed fracture
S12690B	Other displaced fracture of seventh cervical vertebra, initial encounter for open fracture
S12691A	Other nondisplaced fracture of seventh cervical vertebra, initial encounter for closed fracture
S12691B	Other nondisplaced fracture of seventh cervical vertebra, initial encounter for open fracture
S129XXA	Fracture of neck, unspecified, initial encounter
S22000A	Wedge compression fracture of unspecified thoracic vertebra, initial encounter for closed fracture
S22000B	Wedge compression fracture of unspecified thoracic vertebra, initial encounter for open fracture
S22001A	Stable burst fracture of unspecified thoracic vertebra, initial encounter for closed fracture
S22001B	Stable burst fracture of unspecified thoracic vertebra, initial encounter for open fracture
S22002A	Unstable burst fracture of unspecified thoracic vertebra, initial encounter for closed fracture
S22002B	Unstable burst fracture of unspecified thoracic vertebra, initial encounter for open fracture
S22008A	Other fracture of unspecified thoracic vertebra, initial encounter for closed fracture
S22008B	Other fracture of unspecified thoracic vertebra, initial encounter for open fracture
S22009A	Unspecified fracture of unspecified thoracic vertebra, initial encounter for closed fracture
S22009B	Unspecified fracture of unspecified thoracic vertebra, initial encounter for open fracture
S22010A	Wedge compression fracture of first thoracic vertebra, initial encounter for closed fracture
S22010B	Wedge compression fracture of first thoracic vertebra, initial encounter for open fracture
S22011A	Stable burst fracture of first thoracic vertebra, initial encounter for closed fracture
S22011B	Stable burst fracture of first thoracic vertebra, initial encounter for open fracture
S22012A	Unstable burst fracture of first thoracic vertebra, initial encounter for closed fracture
S22012B	Unstable burst fracture of first thoracic vertebra, initial encounter for open fracture
S22018A	Other fracture of first thoracic vertebra, initial encounter for closed fracture
S22018B	Other fracture of first thoracic vertebra, initial encounter for open fracture
S22019A	Unspecified fracture of first thoracic vertebra, initial encounter for closed fracture
S22019B	Unspecified fracture of first thoracic vertebra, initial encounter for open fracture
S22020A	Wedge compression fracture of second thoracic vertebra, initial encounter for closed fracture
S22020B	Wedge compression fracture of second thoracic vertebra, initial encounter for open fracture
S22021A	Stable burst fracture of second thoracic vertebra, initial encounter for closed fracture
S22021B	Stable burst fracture of second thoracic vertebra, initial encounter for open fracture
S22022A	Unstable burst fracture of second thoracic vertebra, initial encounter for closed fracture
S22022B	Unstable burst fracture of second thoracic vertebra, initial encounter for open fracture
S22028A	Other fracture of second thoracic vertebra, initial encounter for closed fracture
S22028B	Other fracture of second thoracic vertebra, initial encounter for open fracture
S22029A	Unspecified fracture of second thoracic vertebra, initial encounter for closed fracture
S22029B	Unspecified fracture of second thoracic vertebra, initial encounter for open fracture
S22030A	Wedge compression fracture of third thoracic vertebra, initial encounter for closed fracture
S22030B	Wedge compression fracture of third thoracic vertebra, initial encounter for open fracture
S22031A	Stable burst fracture of third thoracic vertebra, initial encounter for closed fracture

S22031B	Stable burst fracture of third thoracic vertebra, initial encounter for open fracture
S22032A	Unstable burst fracture of third thoracic vertebra, initial encounter for closed fracture
S22032B	Unstable burst fracture of third thoracic vertebra, initial encounter for open fracture
S22038A	Other fracture of third thoracic vertebra, initial encounter for closed fracture
S22038B	Other fracture of third thoracic vertebra, initial encounter for open fracture
S22039A	Unspecified fracture of third thoracic vertebra, initial encounter for closed fracture
S22039B	Unspecified fracture of third thoracic vertebra, initial encounter for open fracture
S22040A	Wedge compression fracture of fourth thoracic vertebra, initial encounter for closed fracture
S22040B	Wedge compression fracture of fourth thoracic vertebra, initial encounter for open fracture
S22041A	Stable burst fracture of fourth thoracic vertebra, initial encounter for closed fracture
S22041B	Stable burst fracture of fourth thoracic vertebra, initial encounter for open fracture
S22042A	Unstable burst fracture of fourth thoracic vertebra, initial encounter for closed fracture
S22042B	Unstable burst fracture of fourth thoracic vertebra, initial encounter for open fracture
S22048A	Other fracture of fourth thoracic vertebra, initial encounter for closed fracture
S22048B	Other fracture of fourth thoracic vertebra, initial encounter for open fracture
S22049A	Unspecified fracture of fourth thoracic vertebra, initial encounter for closed fracture
S22049B	Unspecified fracture of fourth thoracic vertebra, initial encounter for open fracture
S22050A	Wedge compression fracture of T5-T6 vertebra, initial encounter for closed fracture
S22050B	Wedge compression fracture of T5-T6 vertebra, initial encounter for open fracture
S22051A	Stable burst fracture of T5-T6 vertebra, initial encounter for closed fracture
S22051B	Stable burst fracture of T5-T6 vertebra, initial encounter for open fracture
S22052A	Unstable burst fracture of T5-T6 vertebra, initial encounter for closed fracture
S22052B	Unstable burst fracture of T5-T6 vertebra, initial encounter for open fracture
S22058A	Other fracture of T5-T6 vertebra, initial encounter for closed fracture
S22058B	Other fracture of T5-T6 vertebra, initial encounter for open fracture
S22059A	Unspecified fracture of T5-T6 vertebra, initial encounter for closed fracture
S22059B	Unspecified fracture of T5-T6 vertebra, initial encounter for open fracture
S22060A	Wedge compression fracture of T7-T8 vertebra, initial encounter for closed fracture
S22060B	Wedge compression fracture of T7-T8 vertebra, initial encounter for open fracture
S22061A	Stable burst fracture of T7-T8 vertebra, initial encounter for closed fracture
S22061B	Stable burst fracture of T7-T8 vertebra, initial encounter for open fracture
S22062A	Unstable burst fracture of T7-T8 vertebra, initial encounter for closed fracture
S22062B	Unstable burst fracture of T7-T8 vertebra, initial encounter for open fracture
S22068A	Other fracture of T7-T8 thoracic vertebra, initial encounter for closed fracture
S22068B	Other fracture of T7-T8 thoracic vertebra, initial encounter for open fracture
S22069A	Unspecified fracture of T7-T8 vertebra, initial encounter for closed fracture
S22069B	Unspecified fracture of T7-T8 vertebra, initial encounter for open fracture
S22070A	Wedge compression fracture of T9-T10 vertebra, initial encounter for closed fracture
S22070B	Wedge compression fracture of T9-T10 vertebra, initial encounter for open fracture
S22071A	Stable burst fracture of T9-T10 vertebra, initial encounter for closed fracture
S22071B	Stable burst fracture of T9-T10 vertebra, initial encounter for open fracture
S22072A	Unstable burst fracture of T9-T10 vertebra, initial encounter for closed fracture
S22072B	Unstable burst fracture of T9-T10 vertebra, initial encounter for open fracture

S22078A	Other fracture of T9-T10 vertebra, initial encounter for closed fracture
S22078B	Other fracture of T9-T10 vertebra, initial encounter for open fracture
S22079A	Unspecified fracture of T9-T10 vertebra, initial encounter for closed fracture
S22079B	Unspecified fracture of T9-T10 vertebra, initial encounter for open fracture
S22080A	Wedge compression fracture of T11-T12 vertebra, initial encounter for closed fracture
S22080B	Wedge compression fracture of T11-T12 vertebra, initial encounter for open fracture
S22081A	Stable burst fracture of T11-T12 vertebra, initial encounter for closed fracture
S22081B	Stable burst fracture of T11-T12 vertebra, initial encounter for open fracture
S22082A	Unstable burst fracture of T11-T12 vertebra, initial encounter for closed fracture
S22082B	Unstable burst fracture of T11-T12 vertebra, initial encounter for open fracture
S22088A	Other fracture of T11-T12 vertebra, initial encounter for closed fracture
S22088B	Other fracture of T11-T12 vertebra, initial encounter for open fracture
S22089A	Unspecified fracture of T11-T12 vertebra, initial encounter for closed fracture
S22089B	Unspecified fracture of T11-T12 vertebra, initial encounter for open fracture
S2360	Vertebroplast cerv 1st
S2361	Vertebroplast cerv addl
S32000A	Wedge compression fracture of unspecified lumbar vertebra, initial encounter for closed fracture
S32000B	Wedge compression fracture of unspecified lumbar vertebra, initial encounter for open fracture
S32001A	Stable burst fracture of unspecified lumbar vertebra, initial encounter for closed fracture
S32001B	Stable burst fracture of unspecified lumbar vertebra, initial encounter for open fracture
S32002A	Unstable burst fracture of unspecified lumbar vertebra, initial encounter for closed fracture
S32002B	Unstable burst fracture of unspecified lumbar vertebra, initial encounter for open fracture
S32008A	Other fracture of unspecified lumbar vertebra, initial encounter for closed fracture
S32008B	Other fracture of unspecified lumbar vertebra, initial encounter for open fracture
S32009A	Unspecified fracture of unspecified lumbar vertebra, initial encounter for closed fracture
S32009B	Unspecified fracture of unspecified lumbar vertebra, initial encounter for open fracture
S32010A	Wedge compression fracture of first lumbar vertebra, initial encounter for closed fracture
S32010B	Wedge compression fracture of first lumbar vertebra, initial encounter for open fracture
S32011A	Stable burst fracture of first lumbar vertebra, initial encounter for closed fracture
S32011B	Stable burst fracture of first lumbar vertebra, initial encounter for open fracture
S32012A	Unstable burst fracture of first lumbar vertebra, initial encounter for closed fracture
S32012B	Unstable burst fracture of first lumbar vertebra, initial encounter for open fracture
S32018A	Other fracture of first lumbar vertebra, initial encounter for closed fracture
S32018B	Other fracture of first lumbar vertebra, initial encounter for open fracture
S32019A	Unspecified fracture of first lumbar vertebra, initial encounter for closed fracture
S32019B	Unspecified fracture of first lumbar vertebra, initial encounter for open fracture
S32020A	Wedge compression fracture of second lumbar vertebra, initial encounter for closed fracture
S32020B	Wedge compression fracture of second lumbar vertebra, initial encounter for open fracture
S32021A	Stable burst fracture of second lumbar vertebra, initial encounter for closed fracture
S32021B	Stable burst fracture of second lumbar vertebra, initial encounter for open fracture
S32022A	Unstable burst fracture of second lumbar vertebra, initial encounter for closed fracture
S32022B	Unstable burst fracture of second lumbar vertebra, initial encounter for open fracture
S32028A	Other fracture of second lumbar vertebra, initial encounter for closed fracture

S32028B	Other fracture of second lumbar vertebra, initial encounter for open fracture
S32029A	Unspecified fracture of second lumbar vertebra, initial encounter for closed fracture
S32029B	Unspecified fracture of second lumbar vertebra, initial encounter for open fracture
S32030A	Wedge compression fracture of third lumbar vertebra, initial encounter for closed fracture
S32030B	Wedge compression fracture of third lumbar vertebra, initial encounter for open fracture
S32031A	Stable burst fracture of third lumbar vertebra, initial encounter for closed fracture
S32031B	Stable burst fracture of third lumbar vertebra, initial encounter for open fracture
S32032A	Unstable burst fracture of third lumbar vertebra, initial encounter for closed fracture
S32032B	Unstable burst fracture of third lumbar vertebra, initial encounter for open fracture
S32038A	Other fracture of third lumbar vertebra, initial encounter for closed fracture
S32038B	Other fracture of third lumbar vertebra, initial encounter for open fracture
S32039A	Unspecified fracture of third lumbar vertebra, initial encounter for closed fracture
S32039B	Unspecified fracture of third lumbar vertebra, initial encounter for open fracture
S32040A	Wedge compression fracture of fourth lumbar vertebra, initial encounter for closed fracture
S32040B	Wedge compression fracture of fourth lumbar vertebra, initial encounter for open fracture
S32041A	Stable burst fracture of fourth lumbar vertebra, initial encounter for closed fracture
S32041B	Stable burst fracture of fourth lumbar vertebra, initial encounter for open fracture
S32042A	Unstable burst fracture of fourth lumbar vertebra, initial encounter for closed fracture
S32042B	Unstable burst fracture of fourth lumbar vertebra, initial encounter for open fracture
S32048A	Other fracture of fourth lumbar vertebra, initial encounter for closed fracture
S32048B	Other fracture of fourth lumbar vertebra, initial encounter for open fracture
S32049A	Unspecified fracture of fourth lumbar vertebra, initial encounter for closed fracture
S32049B	Unspecified fracture of fourth lumbar vertebra, initial encounter for open fracture
S32050A	Wedge compression fracture of fifth lumbar vertebra, initial encounter for closed fracture
S32050B	Wedge compression fracture of fifth lumbar vertebra, initial encounter for open fracture
S32051A	Stable burst fracture of fifth lumbar vertebra, initial encounter for closed fracture
S32051B	Stable burst fracture of fifth lumbar vertebra, initial encounter for open fracture
S32052A	Unstable burst fracture of fifth lumbar vertebra, initial encounter for closed fracture
S32052B	Unstable burst fracture of fifth lumbar vertebra, initial encounter for open fracture
S32058A	Other fracture of fifth lumbar vertebra, initial encounter for closed fracture
S32058B	Other fracture of fifth lumbar vertebra, initial encounter for open fracture
S32059A	Unspecified fracture of fifth lumbar vertebra, initial encounter for closed fracture
S32059B	Unspecified fracture of fifth lumbar vertebra, initial encounter for open fracture
S3210XA	Unspecified fracture of sacrum, initial encounter for closed fracture
S3210XB	Unspecified fracture of sacrum, initial encounter for open fracture
S32110A	Nondisplaced Zone I fracture of sacrum, initial encounter for closed fracture
S32110B	Nondisplaced Zone I fracture of sacrum, initial encounter for open fracture
S32111A	Minimally displaced Zone I fracture of sacrum, initial encounter for closed fracture
S32111B	Minimally displaced Zone I fracture of sacrum, initial encounter for open fracture
S32112A	Severely displaced Zone I fracture of sacrum, initial encounter for closed fracture
S32112B	Severely displaced Zone I fracture of sacrum, initial encounter for open fracture
S32119A	Unspecified Zone I fracture of sacrum, initial encounter for closed fracture
S32119B	Unspecified Zone I fracture of sacrum, initial encounter for open fracture

S32120A	Nondisplaced Zone II fracture of sacrum, initial encounter for closed fracture
S32120B	Nondisplaced Zone II fracture of sacrum, initial encounter for open fracture
S32121A	Minimally displaced Zone II fracture of sacrum, initial encounter for closed fracture
S32121B	Minimally displaced Zone II fracture of sacrum, initial encounter for open fracture
S32122A	Severely displaced Zone II fracture of sacrum, initial encounter for closed fracture
S32122B	Severely displaced Zone II fracture of sacrum, initial encounter for open fracture
S32129A	Unspecified Zone II fracture of sacrum, initial encounter for closed fracture
S32129B	Unspecified Zone II fracture of sacrum, initial encounter for open fracture
S32130A	Nondisplaced Zone III fracture of sacrum, initial encounter for closed fracture
S32130B	Nondisplaced Zone III fracture of sacrum, initial encounter for open fracture
S32131A	Minimally displaced Zone III fracture of sacrum, initial encounter for closed fracture
S32131B	Minimally displaced Zone III fracture of sacrum, initial encounter for open fracture
S32132A	Severely displaced Zone III fracture of sacrum, initial encounter for closed fracture
S32132B	Severely displaced Zone III fracture of sacrum, initial encounter for open fracture
S32139A	Unspecified Zone III fracture of sacrum, initial encounter for closed fracture
S32139B	Unspecified Zone III fracture of sacrum, initial encounter for open fracture
S3214XA	Type 1 fracture of sacrum, initial encounter for closed fracture
S3214XB	Type 1 fracture of sacrum, initial encounter for open fracture
S3215XA	Type 2 fracture of sacrum, initial encounter for closed fracture
S3215XB	Type 2 fracture of sacrum, initial encounter for open fracture
S3216XA	Type 3 fracture of sacrum, initial encounter for closed fracture
S3216XB	Type 3 fracture of sacrum, initial encounter for open fracture
S3217XA	Type 4 fracture of sacrum, initial encounter for closed fracture
S3217XB	Type 4 fracture of sacrum, initial encounter for open fracture
S3219XA	Other fracture of sacrum, initial encounter for closed fracture
S3219XB	Other fracture of sacrum, initial encounter for open fracture
S322XXA	Fracture of coccyx, initial encounter for closed fracture
S322XXB	Fracture of coccyx, initial encounter for open fracture
S32301A	Unspecified fracture of right ilium, initial encounter for closed fracture
S32301B	Unspecified fracture of right ilium, initial encounter for open fracture
S32302A	Unspecified fracture of left ilium, initial encounter for closed fracture
S32302B	Unspecified fracture of left ilium, initial encounter for open fracture
S32309A	Unspecified fracture of unspecified ilium, initial encounter for closed fracture
S32309B	Unspecified fracture of unspecified ilium, initial encounter for open fracture
S32311A	Displaced avulsion fracture of right ilium, initial encounter for closed fracture
S32311B	Displaced avulsion fracture of right ilium, initial encounter for open fracture
S32312A	Displaced avulsion fracture of left ilium, initial encounter for closed fracture
S32312B	Displaced avulsion fracture of left ilium, initial encounter for open fracture
S32313A	Displaced avulsion fracture of unspecified ilium, initial encounter for closed fracture
S32313B	Displaced avulsion fracture of unspecified ilium, initial encounter for open fracture
S32314A	Nondisplaced avulsion fracture of right ilium, initial encounter for closed fracture
S32314B	Nondisplaced avulsion fracture of right ilium, initial encounter for open fracture
S32315A	Nondisplaced avulsion fracture of left ilium, initial encounter for closed fracture

S32315B	Nondisplaced avulsion fracture of left ilium, initial encounter for open fracture
S32316A	Nondisplaced avulsion fracture of unspecified ilium, initial encounter for closed fracture
S32316B	Nondisplaced avulsion fracture of unspecified ilium, initial encounter for open fracture
S32391A	Other fracture of right ilium, initial encounter for closed fracture
S32391B	Other fracture of right ilium, initial encounter for open fracture
S32392A	Other fracture of left ilium, initial encounter for closed fracture
S32392B	Other fracture of left ilium, initial encounter for open fracture
S32399A	Other fracture of unspecified ilium, initial encounter for closed fracture
S32399B	Other fracture of unspecified ilium, initial encounter for open fracture
S32401A	Unspecified fracture of right acetabulum, initial encounter for closed fracture
S32401B	Unspecified fracture of right acetabulum, initial encounter for open fracture
S32402A	Unspecified fracture of left acetabulum, initial encounter for closed fracture
S32402B	Unspecified fracture of left acetabulum, initial encounter for open fracture
S32409A	Unspecified fracture of unspecified acetabulum, initial encounter for closed fracture
S32409B	Unspecified fracture of unspecified acetabulum, initial encounter for open fracture
S32411A	Displaced fracture of anterior wall of right acetabulum, initial encounter for closed fracture
S32411B	Displaced fracture of anterior wall of right acetabulum, initial encounter for open fracture
S32412A	Displaced fracture of anterior wall of left acetabulum, initial encounter for closed fracture
S32412B	Displaced fracture of anterior wall of left acetabulum, initial encounter for open fracture
S32413A	Displaced fracture of anterior wall of unspecified acetabulum, initial encounter for closed fracture
S32413B	Displaced fracture of anterior wall of unspecified acetabulum, initial encounter for open fracture
S32414A	Nondisplaced fracture of anterior wall of right acetabulum, initial encounter for closed fracture
S32414B	Nondisplaced fracture of anterior wall of right acetabulum, initial encounter for open fracture
S32415A	Nondisplaced fracture of anterior wall of left acetabulum, initial encounter for closed fracture
S32415B	Nondisplaced fracture of anterior wall of left acetabulum, initial encounter for open fracture
S32416A	Nondisplaced fracture of anterior wall of unspecified acetabulum, initial encounter for closed fracture
S32416B	Nondisplaced fracture of anterior wall of unspecified acetabulum, initial encounter for open fracture
S32421A	Displaced fracture of posterior wall of right acetabulum, initial encounter for closed fracture
S32421B	Displaced fracture of posterior wall of right acetabulum, initial encounter for open fracture
S32422A	Displaced fracture of posterior wall of left acetabulum, initial encounter for closed fracture
S32422B	Displaced fracture of posterior wall of left acetabulum, initial encounter for open fracture
S32423A	Displaced fracture of posterior wall of unspecified acetabulum, initial encounter for closed fracture
S32423B	Displaced fracture of posterior wall of unspecified acetabulum, initial encounter for open fracture
S32424A	Nondisplaced fracture of posterior wall of right acetabulum, initial encounter for closed fracture
S32424B	Nondisplaced fracture of posterior wall of right acetabulum, initial encounter for open fracture
S32425A	Nondisplaced fracture of posterior wall of left acetabulum, initial encounter for closed fracture
S32425B	Nondisplaced fracture of posterior wall of left acetabulum, initial encounter for open fracture
S32426A	Nondisplaced fracture of posterior wall of unspecified acetabulum, initial encounter for closed fracture
S32426B	Nondisplaced fracture of posterior wall of unspecified acetabulum, initial encounter for open fracture
S32431A	Displaced fracture of anterior column [iliopubic] of right acetabulum, initial encounter for closed fracture
S32431B	Displaced fracture of anterior column [iliopubic] of right acetabulum, initial encounter for open fracture
S32432A	Displaced fracture of anterior column [iliopubic] of left acetabulum, initial encounter for closed

	fracture
S32432B	Displaced fracture of anterior column [iliopubic] of left acetabulum, initial encounter for open fracture
S32433A	Displaced fracture of anterior column [iliopubic] of unspecified acetabulum, initial encounter for closed fracture
S32433B	Displaced fracture of anterior column [iliopubic] of unspecified acetabulum, initial encounter for open fracture
S32434A	Nondisplaced fracture of anterior column [iliopubic] of right acetabulum, initial encounter for closed fracture
S32434B	Nondisplaced fracture of anterior column [iliopubic] of right acetabulum, initial encounter for open fracture
S32435A	Nondisplaced fracture of anterior column [iliopubic] of left acetabulum, initial encounter for closed fracture
S32435B	Nondisplaced fracture of anterior column [iliopubic] of left acetabulum, initial encounter for open fracture
S32436A	Nondisplaced fracture of anterior column [iliopubic] of unspecified acetabulum, initial encounter for closed fracture
S32436B	Nondisplaced fracture of anterior column [iliopubic] of unspecified acetabulum, initial encounter for open fracture
S32441A	Displaced fracture of posterior column [ilioischial] of right acetabulum, initial encounter for closed fracture
S32441B	Displaced fracture of posterior column [ilioischial] of right acetabulum, initial encounter for open fracture
S32442A	Displaced fracture of posterior column [ilioischial] of left acetabulum, initial encounter for closed fracture
S32442B	Displaced fracture of posterior column [ilioischial] of left acetabulum, initial encounter for open fracture
S32443A	Displaced fracture of posterior column [ilioischial] of unspecified acetabulum, initial encounter for closed fracture
S32443B	Displaced fracture of posterior column [ilioischial] of unspecified acetabulum, initial encounter for open fracture
S32444A	Nondisplaced fracture of posterior column [ilioischial] of right acetabulum, initial encounter for closed fracture
S32444B	Nondisplaced fracture of posterior column [ilioischial] of right acetabulum, initial encounter for open fracture
S32445A	Nondisplaced fracture of posterior column [ilioischial] of left acetabulum, initial encounter for closed fracture
S32445B	Nondisplaced fracture of posterior column [ilioischial] of left acetabulum, initial encounter for open fracture
S32446A	Nondisplaced fracture of posterior column [ilioischial] of unspecified acetabulum, initial encounter for closed fracture
S32446B	Nondisplaced fracture of posterior column [ilioischial] of unspecified acetabulum, initial encounter for open fracture
S32451A	Displaced transverse fracture of right acetabulum, initial encounter for closed fracture
S32451B	Displaced transverse fracture of right acetabulum, initial encounter for open fracture
S32452A	Displaced transverse fracture of left acetabulum, initial encounter for closed fracture
S32452B	Displaced transverse fracture of left acetabulum, initial encounter for open fracture
S32453A	Displaced transverse fracture of unspecified acetabulum, initial encounter for closed fracture
S32453B	Displaced transverse fracture of unspecified acetabulum, initial encounter for open fracture
S32454A	Nondisplaced transverse fracture of right acetabulum, initial encounter for closed fracture
S32454B	Nondisplaced transverse fracture of right acetabulum, initial encounter for open fracture

S32455A	Nondisplaced transverse fracture of left acetabulum, initial encounter for closed fracture
S32455B	Nondisplaced transverse fracture of left acetabulum, initial encounter for open fracture
S32456A	Nondisplaced transverse fracture of unspecified acetabulum, initial encounter for closed fracture
S32456B	Nondisplaced transverse fracture of unspecified acetabulum, initial encounter for open fracture
S32461A	Displaced associated transverse-posterior fracture of right acetabulum, initial encounter for closed fracture
S32461B	Displaced associated transverse-posterior fracture of right acetabulum, initial encounter for open fracture
S32462A	Displaced associated transverse-posterior fracture of left acetabulum, initial encounter for closed fracture
S32462B	Displaced associated transverse-posterior fracture of left acetabulum, initial encounter for open fracture
S32463A	Displaced associated transverse-posterior fracture of unspecified acetabulum, initial encounter for closed fracture
S32463B	Displaced associated transverse-posterior fracture of unspecified acetabulum, initial encounter for open fracture
S32464A	Nondisplaced associated transverse-posterior fracture of right acetabulum, initial encounter for closed fracture
S32464B	Nondisplaced associated transverse-posterior fracture of right acetabulum, initial encounter for open fracture
S32465A	Nondisplaced associated transverse-posterior fracture of left acetabulum, initial encounter for closed fracture
S32465B	Nondisplaced associated transverse-posterior fracture of left acetabulum, initial encounter for open fracture
S32466A	Nondisplaced associated transverse-posterior fracture of unspecified acetabulum, initial encounter for closed fracture
S32466B	Nondisplaced associated transverse-posterior fracture of unspecified acetabulum, initial encounter for open fracture
S32471A	Displaced fracture of medial wall of right acetabulum, initial encounter for closed fracture
S32471B	Displaced fracture of medial wall of right acetabulum, initial encounter for open fracture
S32472A	Displaced fracture of medial wall of left acetabulum, initial encounter for closed fracture
S32472B	Displaced fracture of medial wall of left acetabulum, initial encounter for open fracture
S32473A	Displaced fracture of medial wall of unspecified acetabulum, initial encounter for closed fracture
S32473B	Displaced fracture of medial wall of unspecified acetabulum, initial encounter for open fracture
S32474A	Nondisplaced fracture of medial wall of right acetabulum, initial encounter for closed fracture
S32474B	Nondisplaced fracture of medial wall of right acetabulum, initial encounter for open fracture
S32475A	Nondisplaced fracture of medial wall of left acetabulum, initial encounter for closed fracture
S32475B	Nondisplaced fracture of medial wall of left acetabulum, initial encounter for open fracture
S32476A	Nondisplaced fracture of medial wall of unspecified acetabulum, initial encounter for closed fracture
S32476B	Nondisplaced fracture of medial wall of unspecified acetabulum, initial encounter for open fracture
S32481A	Displaced dome fracture of right acetabulum, initial encounter for closed fracture
S32481B	Displaced dome fracture of right acetabulum, initial encounter for open fracture
S32482A	Displaced dome fracture of left acetabulum, initial encounter for closed fracture
S32482B	Displaced dome fracture of left acetabulum, initial encounter for open fracture
S32483A	Displaced dome fracture of unspecified acetabulum, initial encounter for closed fracture
S32483B	Displaced dome fracture of unspecified acetabulum, initial encounter for open fracture
S32484A	Nondisplaced dome fracture of right acetabulum, initial encounter for closed fracture

S32484B	Nondisplaced dome fracture of right acetabulum, initial encounter for open fracture
S32485A	Nondisplaced dome fracture of left acetabulum, initial encounter for closed fracture
S32485B	Nondisplaced dome fracture of left acetabulum, initial encounter for open fracture
S32486A	Nondisplaced dome fracture of unspecified acetabulum, initial encounter for closed fracture
S32486B	Nondisplaced dome fracture of unspecified acetabulum, initial encounter for open fracture
S32491A	Other specified fracture of right acetabulum, initial encounter for closed fracture
S32491B	Other specified fracture of right acetabulum, initial encounter for open fracture
S32492A	Other specified fracture of left acetabulum, initial encounter for closed fracture
S32492B	Other specified fracture of left acetabulum, initial encounter for open fracture
S32499A	Other specified fracture of unspecified acetabulum, initial encounter for closed fracture
S32499B	Other specified fracture of unspecified acetabulum, initial encounter for open fracture
S32501A	Unspecified fracture of right pubis, initial encounter for closed fracture
S32501B	Unspecified fracture of right pubis, initial encounter for open fracture
S32502A	Unspecified fracture of left pubis, initial encounter for closed fracture
S32502B	Unspecified fracture of left pubis, initial encounter for open fracture
S32509A	Unspecified fracture of unspecified pubis, initial encounter for closed fracture
S32509B	Unspecified fracture of unspecified pubis, initial encounter for open fracture
S32511A	Fracture of superior rim of right pubis, initial encounter for closed fracture
S32511B	Fracture of superior rim of right pubis, initial encounter for open fracture
S32512A	Fracture of superior rim of left pubis, initial encounter for closed fracture
S32512B	Fracture of superior rim of left pubis, initial encounter for open fracture
S32519A	Fracture of superior rim of unspecified pubis, initial encounter for closed fracture
S32519B	Fracture of superior rim of unspecified pubis, initial encounter for open fracture
S32591A	Other specified fracture of right pubis, initial encounter for closed fracture
S32591B	Other specified fracture of right pubis, initial encounter for open fracture
S32592A	Other specified fracture of left pubis, initial encounter for closed fracture
S32592B	Other specified fracture of left pubis, initial encounter for open fracture
S32599A	Other specified fracture of unspecified pubis, initial encounter for closed fracture
S32599B	Other specified fracture of unspecified pubis, initial encounter for open fracture
S32601A	Unspecified fracture of right ischium, initial encounter for closed fracture
S32601B	Unspecified fracture of right ischium, initial encounter for open fracture
S32602A	Unspecified fracture of left ischium, initial encounter for closed fracture
S32602B	Unspecified fracture of left ischium, initial encounter for open fracture
S32609A	Unspecified fracture of unspecified ischium, initial encounter for closed fracture
S32609B	Unspecified fracture of unspecified ischium, initial encounter for open fracture
S32611A	Displaced avulsion fracture of right ischium, initial encounter for closed fracture
S32611B	Displaced avulsion fracture of right ischium, initial encounter for open fracture
S32612A	Displaced avulsion fracture of left ischium, initial encounter for closed fracture
S32612B	Displaced avulsion fracture of left ischium, initial encounter for open fracture
S32613A	Displaced avulsion fracture of unspecified ischium, initial encounter for closed fracture
S32613B	Displaced avulsion fracture of unspecified ischium, initial encounter for open fracture
S32614A	Nondisplaced avulsion fracture of right ischium, initial encounter for closed fracture
S32614B	Nondisplaced avulsion fracture of right ischium, initial encounter for open fracture

S32615A	Nondisplaced avulsion fracture of left ischium, initial encounter for closed fracture
S32615B	Nondisplaced avulsion fracture of left ischium, initial encounter for open fracture
S32616A	Nondisplaced avulsion fracture of unspecified ischium, initial encounter for closed fracture
S32616B	Nondisplaced avulsion fracture of unspecified ischium, initial encounter for open fracture
S32691A	Other specified fracture of right ischium, initial encounter for closed fracture
S32691B	Other specified fracture of right ischium, initial encounter for open fracture
S32692A	Other specified fracture of left ischium, initial encounter for closed fracture
S32692B	Other specified fracture of left ischium, initial encounter for open fracture
S32699A	Other specified fracture of unspecified ischium, initial encounter for closed fracture
S32699B	Other specified fracture of unspecified ischium, initial encounter for open fracture
S32810A	Multiple fractures of pelvis with stable disruption of pelvic ring, initial encounter for closed fracture
S32810B	Multiple fractures of pelvis with stable disruption of pelvic ring, initial encounter for open fracture
S32811A	Multiple fractures of pelvis with unstable disruption of pelvic ring, initial encounter for closed fracture
S32811B	Multiple fractures of pelvis with unstable disruption of pelvic ring, initial encounter for open fracture
S3282XA	Multiple fractures of pelvis without disruption of pelvic ring, initial encounter for closed fracture
S3282XB	Multiple fractures of pelvis without disruption of pelvic ring, initial encounter for open fracture
S3289XA	Fracture of other parts of pelvis, initial encounter for closed fracture
S3289XB	Fracture of other parts of pelvis, initial encounter for open fracture
S329XXA	Fracture of unspecified parts of lumbosacral spine and pelvis, initial encounter for closed fracture
S329XXB	Fracture of unspecified parts of lumbosacral spine and pelvis, initial encounter for open fracture
S42001A	Fracture of unspecified part of right clavicle, initial encounter for closed fracture
S42001B	Fracture of unspecified part of right clavicle, initial encounter for open fracture
S42002A	Fracture of unspecified part of left clavicle, initial encounter for closed fracture
S42002B	Fracture of unspecified part of left clavicle, initial encounter for open fracture
S42009A	Fracture of unspecified part of unspecified clavicle, initial encounter for closed fracture
S42009B	Fracture of unspecified part of unspecified clavicle, initial encounter for open fracture
S42011A	Anterior displaced fracture of sternal end of right clavicle, initial encounter for closed fracture
S42011B	Anterior displaced fracture of sternal end of right clavicle, initial encounter for open fracture
S42012A	Anterior displaced fracture of sternal end of left clavicle, initial encounter for closed fracture
S42012B	Anterior displaced fracture of sternal end of left clavicle, initial encounter for open fracture
S42013A	Anterior displaced fracture of sternal end of unspecified clavicle, initial encounter for closed fracture
S42013B	Anterior displaced fracture of sternal end of unspecified clavicle, initial encounter for open fracture
S42014A	Posterior displaced fracture of sternal end of right clavicle, initial encounter for closed fracture
S42014B	Posterior displaced fracture of sternal end of right clavicle, initial encounter for open fracture
S42015A	Posterior displaced fracture of sternal end of left clavicle, initial encounter for closed fracture
S42015B	Posterior displaced fracture of sternal end of left clavicle, initial encounter for open fracture
S42016A	Posterior displaced fracture of sternal end of unspecified clavicle, initial encounter for closed fracture
S42016B	Posterior displaced fracture of sternal end of unspecified clavicle, initial encounter for open fracture
S42017A	Nondisplaced fracture of sternal end of right clavicle, initial encounter for closed fracture
S42017B	Nondisplaced fracture of sternal end of right clavicle, initial encounter for open fracture
S42018A	Nondisplaced fracture of sternal end of left clavicle, initial encounter for closed fracture
S42018B	Nondisplaced fracture of sternal end of left clavicle, initial encounter for open fracture
S42019A	Nondisplaced fracture of sternal end of unspecified clavicle, initial encounter for closed fracture

S42019B	Nondisplaced fracture of sternal end of unspecified clavicle, initial encounter for open fracture
S42021A	Displaced fracture of shaft of right clavicle, initial encounter for closed fracture
S42021B	Displaced fracture of shaft of right clavicle, initial encounter for open fracture
S42022A	Displaced fracture of shaft of left clavicle, initial encounter for closed fracture
S42022B	Displaced fracture of shaft of left clavicle, initial encounter for open fracture
S42023A	Displaced fracture of shaft of unspecified clavicle, initial encounter for closed fracture
S42023B	Displaced fracture of shaft of unspecified clavicle, initial encounter for open fracture
S42024A	Nondisplaced fracture of shaft of right clavicle, initial encounter for closed fracture
S42024B	Nondisplaced fracture of shaft of right clavicle, initial encounter for open fracture
S42025A	Nondisplaced fracture of shaft of left clavicle, initial encounter for closed fracture
S42025B	Nondisplaced fracture of shaft of left clavicle, initial encounter for open fracture
S42026A	Nondisplaced fracture of shaft of unspecified clavicle, initial encounter for closed fracture
S42026B	Nondisplaced fracture of shaft of unspecified clavicle, initial encounter for open fracture
S42031A	Displaced fracture of lateral end of right clavicle, initial encounter for closed fracture
S42031B	Displaced fracture of lateral end of right clavicle, initial encounter for open fracture
S42032A	Displaced fracture of lateral end of left clavicle, initial encounter for closed fracture
S42032B	Displaced fracture of lateral end of left clavicle, initial encounter for open fracture
S42033A	Displaced fracture of lateral end of unspecified clavicle, initial encounter for closed fracture
S42033B	Displaced fracture of lateral end of unspecified clavicle, initial encounter for open fracture
S42034A	Nondisplaced fracture of lateral end of right clavicle, initial encounter for closed fracture
S42034B	Nondisplaced fracture of lateral end of right clavicle, initial encounter for open fracture
S42035A	Nondisplaced fracture of lateral end of left clavicle, initial encounter for closed fracture
S42035B	Nondisplaced fracture of lateral end of left clavicle, initial encounter for open fracture
S42036A	Nondisplaced fracture of lateral end of unspecified clavicle, initial encounter for closed fracture
S42036B	Nondisplaced fracture of lateral end of unspecified clavicle, initial encounter for open fracture
S42201A	Unspecified fracture of upper end of right humerus, initial encounter for closed fracture
S42201B	Unspecified fracture of upper end of right humerus, initial encounter for open fracture
S42202A	Unspecified fracture of upper end of left humerus, initial encounter for closed fracture
S42202B	Unspecified fracture of upper end of left humerus, initial encounter for open fracture
S42209A	Unspecified fracture of upper end of unspecified humerus, initial encounter for closed fracture
S42209B	Unspecified fracture of upper end of unspecified humerus, initial encounter for open fracture
S42211A	Unspecified displaced fracture of surgical neck of right humerus, initial encounter for closed fracture
S42211B	Unspecified displaced fracture of surgical neck of right humerus, initial encounter for open fracture
S42212A	Unspecified displaced fracture of surgical neck of left humerus, initial encounter for closed fracture
S42212B	Unspecified displaced fracture of surgical neck of left humerus, initial encounter for open fracture
S42213A	Unspecified displaced fracture of surgical neck of unspecified humerus, initial encounter for closed fracture
S42213B	Unspecified displaced fracture of surgical neck of unspecified humerus, initial encounter for open fracture
S42214A	Unspecified nondisplaced fracture of surgical neck of right humerus, initial encounter for closed fracture
S42214B	Unspecified nondisplaced fracture of surgical neck of right humerus, initial encounter for open fracture
S42215A	Unspecified nondisplaced fracture of surgical neck of left humerus, initial encounter for closed fracture
S42215B	Unspecified nondisplaced fracture of surgical neck of left humerus, initial encounter for open fracture

S42216A	Unspecified nondisplaced fracture of surgical neck of unspecified humerus, initial encounter for closed fracture
S42216B	Unspecified nondisplaced fracture of surgical neck of unspecified humerus, initial encounter for open fracture
S42221A	2-part displaced fracture of surgical neck of right humerus, initial encounter for closed fracture
S42221B	2-part displaced fracture of surgical neck of right humerus, initial encounter for open fracture
S42222A	2-part displaced fracture of surgical neck of left humerus, initial encounter for closed fracture
S42222B	2-part displaced fracture of surgical neck of left humerus, initial encounter for open fracture
S42223A	2-part displaced fracture of surgical neck of unspecified humerus, initial encounter for closed fracture
S42223B	2-part displaced fracture of surgical neck of unspecified humerus, initial encounter for open fracture
S42224A	2-part nondisplaced fracture of surgical neck of right humerus, initial encounter for closed fracture
S42224B	2-part nondisplaced fracture of surgical neck of right humerus, initial encounter for open fracture
S42225A	2-part nondisplaced fracture of surgical neck of left humerus, initial encounter for closed fracture
S42225B	2-part nondisplaced fracture of surgical neck of left humerus, initial encounter for open fracture
S42226A	2-part nondisplaced fracture of surgical neck of unspecified humerus, initial encounter for closed fracture
S42226B	2-part nondisplaced fracture of surgical neck of unspecified humerus, initial encounter for open fracture
S42231A	3-part fracture of surgical neck of right humerus, initial encounter for closed fracture
S42231B	3-part fracture of surgical neck of right humerus, initial encounter for open fracture
S42232A	3-part fracture of surgical neck of left humerus, initial encounter for closed fracture
S42232B	3-part fracture of surgical neck of left humerus, initial encounter for open fracture
S42239A	3-part fracture of surgical neck of unspecified humerus, initial encounter for closed fracture
S42239B	3-part fracture of surgical neck of unspecified humerus, initial encounter for open fracture
S42241A	4-part fracture of surgical neck of right humerus, initial encounter for closed fracture
S42241B	4-part fracture of surgical neck of right humerus, initial encounter for open fracture
S42242A	4-part fracture of surgical neck of left humerus, initial encounter for closed fracture
S42242B	4-part fracture of surgical neck of left humerus, initial encounter for open fracture
S42249A	4-part fracture of surgical neck of unspecified humerus, initial encounter for closed fracture
S42249B	4-part fracture of surgical neck of unspecified humerus, initial encounter for open fracture
S42251A	Displaced fracture of greater tuberosity of right humerus, initial encounter for closed fracture
S42251B	Displaced fracture of greater tuberosity of right humerus, initial encounter for open fracture
S42252A	Displaced fracture of greater tuberosity of left humerus, initial encounter for closed fracture
S42252B	Displaced fracture of greater tuberosity of left humerus, initial encounter for open fracture
S42253A	Displaced fracture of greater tuberosity of unspecified humerus, initial encounter for closed fracture
S42253B	Displaced fracture of greater tuberosity of unspecified humerus, initial encounter for open fracture
S42254A	Nondisplaced fracture of greater tuberosity of right humerus, initial encounter for closed fracture
S42254B	Nondisplaced fracture of greater tuberosity of right humerus, initial encounter for open fracture
S42255A	Nondisplaced fracture of greater tuberosity of left humerus, initial encounter for closed fracture
S42255B	Nondisplaced fracture of greater tuberosity of left humerus, initial encounter for open fracture
S42256A	Nondisplaced fracture of greater tuberosity of unspecified humerus, initial encounter for closed fracture
S42256B	Nondisplaced fracture of greater tuberosity of unspecified humerus, initial encounter for open fracture
S42261A	Displaced fracture of lesser tuberosity of right humerus, initial encounter for closed fracture
S42261B	Displaced fracture of lesser tuberosity of right humerus, initial encounter for open fracture

S42262A	Displaced fracture of lesser tuberosity of left humerus, initial encounter for closed fracture
S42262B	Displaced fracture of lesser tuberosity of left humerus, initial encounter for open fracture
S42263A	Displaced fracture of lesser tuberosity of unspecified humerus, initial encounter for closed fracture
S42263B	Displaced fracture of lesser tuberosity of unspecified humerus, initial encounter for open fracture
S42264A	Nondisplaced fracture of lesser tuberosity of right humerus, initial encounter for closed fracture
S42264B	Nondisplaced fracture of lesser tuberosity of right humerus, initial encounter for open fracture
S42265A	Nondisplaced fracture of lesser tuberosity of left humerus, initial encounter for closed fracture
S42265B	Nondisplaced fracture of lesser tuberosity of left humerus, initial encounter for open fracture
S42266A	Nondisplaced fracture of lesser tuberosity of unspecified humerus, initial encounter for closed fracture
S42266B	Nondisplaced fracture of lesser tuberosity of unspecified humerus, initial encounter for open fracture
S42271A	Torus fracture of upper end of right humerus, initial encounter for closed fracture
S42272A	Torus fracture of upper end of left humerus, initial encounter for closed fracture
S42279A	Torus fracture of upper end of unspecified humerus, initial encounter for closed fracture
S42291A	Other displaced fracture of upper end of right humerus, initial encounter for closed fracture
S42291B	Other displaced fracture of upper end of right humerus, initial encounter for open fracture
S42292A	Other displaced fracture of upper end of left humerus, initial encounter for closed fracture
S42292B	Other displaced fracture of upper end of left humerus, initial encounter for open fracture
S42293A	Other displaced fracture of upper end of unspecified humerus, initial encounter for closed fracture
S42293B	Other displaced fracture of upper end of unspecified humerus, initial encounter for open fracture
S42294A	Other nondisplaced fracture of upper end of right humerus, initial encounter for closed fracture
S42294B	Other nondisplaced fracture of upper end of right humerus, initial encounter for open fracture
S42295A	Other nondisplaced fracture of upper end of left humerus, initial encounter for closed fracture
S42295B	Other nondisplaced fracture of upper end of left humerus, initial encounter for open fracture
S42296A	Other nondisplaced fracture of upper end of unspecified humerus, initial encounter for closed fracture
S42296B	Other nondisplaced fracture of upper end of unspecified humerus, initial encounter for open fracture
S42301A	Unspecified fracture of shaft of humerus, right arm, initial encounter for closed fracture
S42301B	Unspecified fracture of shaft of humerus, right arm, initial encounter for open fracture
S42302A	Unspecified fracture of shaft of humerus, left arm, initial encounter for closed fracture
S42302B	Unspecified fracture of shaft of humerus, left arm, initial encounter for open fracture
S42309A	Unspecified fracture of shaft of humerus, unspecified arm, initial encounter for closed fracture
S42309B	Unspecified fracture of shaft of humerus, unspecified arm, initial encounter for open fracture
S42311A	Greenstick fracture of shaft of humerus, right arm, initial encounter for closed fracture
S42312A	Greenstick fracture of shaft of humerus, left arm, initial encounter for closed fracture
S42319A	Greenstick fracture of shaft of humerus, unspecified arm, initial encounter for closed fracture
S42321A	Displaced transverse fracture of shaft of humerus, right arm, initial encounter for closed fracture
S42321B	Displaced transverse fracture of shaft of humerus, right arm, initial encounter for open fracture
S42322A	Displaced transverse fracture of shaft of humerus, left arm, initial encounter for closed fracture
S42322B	Displaced transverse fracture of shaft of humerus, left arm, initial encounter for open fracture
S42323A	Displaced transverse fracture of shaft of humerus, unspecified arm, initial encounter for closed fracture
S42323B	Displaced transverse fracture of shaft of humerus, unspecified arm, initial encounter for open fracture
S42324A	Nondisplaced transverse fracture of shaft of humerus, right arm, initial encounter for closed fracture
S42324B	Nondisplaced transverse fracture of shaft of humerus, right arm, initial encounter for open fracture

S42325A	Nondisplaced transverse fracture of shaft of humerus, left arm, initial encounter for closed fracture
S42325B	Nondisplaced transverse fracture of shaft of humerus, left arm, initial encounter for open fracture
S42326A	Nondisplaced transverse fracture of shaft of humerus, unspecified arm, initial encounter for closed fracture
S42326B	Nondisplaced transverse fracture of shaft of humerus, unspecified arm, initial encounter for open fracture
S42331A	Displaced oblique fracture of shaft of humerus, right arm, initial encounter for closed fracture
S42331B	Displaced oblique fracture of shaft of humerus, right arm, initial encounter for open fracture
S42332A	Displaced oblique fracture of shaft of humerus, left arm, initial encounter for closed fracture
S42332B	Displaced oblique fracture of shaft of humerus, left arm, initial encounter for open fracture
S42333A	Displaced oblique fracture of shaft of humerus, unspecified arm, initial encounter for closed fracture
S42333B	Displaced oblique fracture of shaft of humerus, unspecified arm, initial encounter for open fracture
S42334A	Nondisplaced oblique fracture of shaft of humerus, right arm, initial encounter for closed fracture
S42334B	Nondisplaced oblique fracture of shaft of humerus, right arm, initial encounter for open fracture
S42335A	Nondisplaced oblique fracture of shaft of humerus, left arm, initial encounter for closed fracture
S42335B	Nondisplaced oblique fracture of shaft of humerus, left arm, initial encounter for open fracture
S42336A	Nondisplaced oblique fracture of shaft of humerus, unspecified arm, initial encounter for closed fracture
S42336B	Nondisplaced oblique fracture of shaft of humerus, unspecified arm, initial encounter for open fracture
S42341A	Displaced spiral fracture of shaft of humerus, right arm, initial encounter for closed fracture
S42341B	Displaced spiral fracture of shaft of humerus, right arm, initial encounter for open fracture
S42342A	Displaced spiral fracture of shaft of humerus, left arm, initial encounter for closed fracture
S42342B	Displaced spiral fracture of shaft of humerus, left arm, initial encounter for open fracture
S42343A	Displaced spiral fracture of shaft of humerus, unspecified arm, initial encounter for closed fracture
S42343B	Displaced spiral fracture of shaft of humerus, unspecified arm, initial encounter for open fracture
S42344A	Nondisplaced spiral fracture of shaft of humerus, right arm, initial encounter for closed fracture
S42344B	Nondisplaced spiral fracture of shaft of humerus, right arm, initial encounter for open fracture
S42345A	Nondisplaced spiral fracture of shaft of humerus, left arm, initial encounter for closed fracture
S42345B	Nondisplaced spiral fracture of shaft of humerus, left arm, initial encounter for open fracture
S42346A	Nondisplaced spiral fracture of shaft of humerus, unspecified arm, initial encounter for closed fracture
S42346B	Nondisplaced spiral fracture of shaft of humerus, unspecified arm, initial encounter for open fracture
S42351A	Displaced comminuted fracture of shaft of humerus, right arm, initial encounter for closed fracture
S42351B	Displaced comminuted fracture of shaft of humerus, right arm, initial encounter for open fracture
S42352A	Displaced comminuted fracture of shaft of humerus, left arm, initial encounter for closed fracture
S42352B	Displaced comminuted fracture of shaft of humerus, left arm, initial encounter for open fracture
S42353A	Displaced comminuted fracture of shaft of humerus, unspecified arm, initial encounter for closed fracture
S42353B	Displaced comminuted fracture of shaft of humerus, unspecified arm, initial encounter for open fracture
S42354A	Nondisplaced comminuted fracture of shaft of humerus, right arm, initial encounter for closed fracture
S42354B	Nondisplaced comminuted fracture of shaft of humerus, right arm, initial encounter for open fracture
S42355A	Nondisplaced comminuted fracture of shaft of humerus, left arm, initial encounter for closed fracture
S42355B	Nondisplaced comminuted fracture of shaft of humerus, left arm, initial encounter for open fracture
S42356A	Nondisplaced comminuted fracture of shaft of humerus, unspecified arm, initial encounter for closed fracture

S42356B	Nondisplaced comminuted fracture of shaft of humerus, unspecified arm, initial encounter for open fracture
S42361A	Displaced segmental fracture of shaft of humerus, right arm, initial encounter for closed fracture
S42361B	Displaced segmental fracture of shaft of humerus, right arm, initial encounter for open fracture
S42362A	Displaced segmental fracture of shaft of humerus, left arm, initial encounter for closed fracture
S42362B	Displaced segmental fracture of shaft of humerus, left arm, initial encounter for open fracture
S42363A	Displaced segmental fracture of shaft of humerus, unspecified arm, initial encounter for closed fracture
S42363B	Displaced segmental fracture of shaft of humerus, unspecified arm, initial encounter for open fracture
S42364A	Nondisplaced segmental fracture of shaft of humerus, right arm, initial encounter for closed fracture
S42364B	Nondisplaced segmental fracture of shaft of humerus, right arm, initial encounter for open fracture
S42365A	Nondisplaced segmental fracture of shaft of humerus, left arm, initial encounter for closed fracture
S42365B	Nondisplaced segmental fracture of shaft of humerus, left arm, initial encounter for open fracture
S42366A	Nondisplaced segmental fracture of shaft of humerus, unspecified arm, initial encounter for closed fracture
S42366B	Nondisplaced segmental fracture of shaft of humerus, unspecified arm, initial encounter for open fracture
S42391A	Other fracture of shaft of right humerus, initial encounter for closed fracture
S42391B	Other fracture of shaft of right humerus, initial encounter for open fracture
S42392A	Other fracture of shaft of left humerus, initial encounter for closed fracture
S42392B	Other fracture of shaft of left humerus, initial encounter for open fracture
S42399A	Other fracture of shaft of unspecified humerus, initial encounter for closed fracture
S42399B	Other fracture of shaft of unspecified humerus, initial encounter for open fracture
S42401A	Unspecified fracture of lower end of right humerus, initial encounter for closed fracture
S42401B	Unspecified fracture of lower end of right humerus, initial encounter for open fracture
S42402A	Unspecified fracture of lower end of left humerus, initial encounter for closed fracture
S42402B	Unspecified fracture of lower end of left humerus, initial encounter for open fracture
S42409A	Unspecified fracture of lower end of unspecified humerus, initial encounter for closed fracture
S42409B	Unspecified fracture of lower end of unspecified humerus, initial encounter for open fracture
S42411A	Displaced simple supracondylar fracture without intercondylar fracture of right humerus, initial encounter for closed fracture
S42411B	Displaced simple supracondylar fracture without intercondylar fracture of right humerus, initial encounter for open fracture
S42412A	Displaced simple supracondylar fracture without intercondylar fracture of left humerus, initial encounter for closed fracture
S42412B	Displaced simple supracondylar fracture without intercondylar fracture of left humerus, initial encounter for open fracture
S42413A	Displaced simple supracondylar fracture without intercondylar fracture of unspecified humerus, initial encounter for closed fracture
S42413B	Displaced simple supracondylar fracture without intercondylar fracture of unspecified humerus, initial encounter for open fracture
S42414A	Nondisplaced simple supracondylar fracture without intercondylar fracture of right humerus, initial encounter for closed fracture
S42414B	Nondisplaced simple supracondylar fracture without intercondylar fracture of right humerus, initial encounter for open fracture
S42415A	Nondisplaced simple supracondylar fracture without intercondylar fracture of left humerus, initial encounter for closed fracture

S42415B	Nondisplaced simple supracondylar fracture without intercondylar fracture of left humerus, initial encounter for open fracture
S42416A	Nondisplaced simple supracondylar fracture without intercondylar fracture of unspecified humerus, initial encounter for closed fracture
S42416B	Nondisplaced simple supracondylar fracture without intercondylar fracture of unspecified humerus, initial encounter for open fracture
S42421A	Displaced comminuted supracondylar fracture without intercondylar fracture of right humerus, initial encounter for closed fracture
S42421B	Displaced comminuted supracondylar fracture without intercondylar fracture of right humerus, initial encounter for open fracture
S42422A	Displaced comminuted supracondylar fracture without intercondylar fracture of left humerus, initial encounter for closed fracture
S42422B	Displaced comminuted supracondylar fracture without intercondylar fracture of left humerus, initial encounter for open fracture
S42423A	Displaced comminuted supracondylar fracture without intercondylar fracture of unspecified humerus, initial encounter for closed fracture
S42423B	Displaced comminuted supracondylar fracture without intercondylar fracture of unspecified humerus, initial encounter for open fracture
S42424A	Nondisplaced comminuted supracondylar fracture without intercondylar fracture of right humerus, initial encounter for closed fracture
S42424B	Nondisplaced comminuted supracondylar fracture without intercondylar fracture of right humerus, initial encounter for open fracture
S42425A	Nondisplaced comminuted supracondylar fracture without intercondylar fracture of left humerus, initial encounter for closed fracture
S42425B	Nondisplaced comminuted supracondylar fracture without intercondylar fracture of left humerus, initial encounter for open fracture
S42426A	Nondisplaced comminuted supracondylar fracture without intercondylar fracture of unspecified humerus, initial encounter for closed fracture
S42426B	Nondisplaced comminuted supracondylar fracture without intercondylar fracture of unspecified humerus, initial encounter for open fracture
S42431A	Displaced fracture (avulsion) of lateral epicondyle of right humerus, initial encounter for closed fracture
S42431B	Displaced fracture (avulsion) of lateral epicondyle of right humerus, initial encounter for open fracture
S42432A	Displaced fracture (avulsion) of lateral epicondyle of left humerus, initial encounter for closed fracture
S42432B	Displaced fracture (avulsion) of lateral epicondyle of left humerus, initial encounter for open fracture
S42433A	Displaced fracture (avulsion) of lateral epicondyle of unspecified humerus, initial encounter for closed fracture
S42433B	Displaced fracture (avulsion) of lateral epicondyle of unspecified humerus, initial encounter for open fracture
S42434A	Nondisplaced fracture (avulsion) of lateral epicondyle of right humerus, initial encounter for closed fracture
S42434B	Nondisplaced fracture (avulsion) of lateral epicondyle of right humerus, initial encounter for open fracture
S42435A	Nondisplaced fracture (avulsion) of lateral epicondyle of left humerus, initial encounter for closed fracture
S42435B	Nondisplaced fracture (avulsion) of lateral epicondyle of left humerus, initial encounter for open fracture
S42436A	Nondisplaced fracture (avulsion) of lateral epicondyle of unspecified humerus, initial encounter for closed fracture
S42436B	Nondisplaced fracture (avulsion) of lateral epicondyle of unspecified humerus, initial encounter for open fracture

S42441A	Displaced fracture (avulsion) of medial epicondyle of right humerus, initial encounter for closed fracture
S42441B	Displaced fracture (avulsion) of medial epicondyle of right humerus, initial encounter for open fracture
S42442A	Displaced fracture (avulsion) of medial epicondyle of left humerus, initial encounter for closed fracture
S42442B	Displaced fracture (avulsion) of medial epicondyle of left humerus, initial encounter for open fracture
S42443A	Displaced fracture (avulsion) of medial epicondyle of unspecified humerus, initial encounter for closed fracture
S42443B	Displaced fracture (avulsion) of medial epicondyle of unspecified humerus, initial encounter for open fracture
S42444A	Nondisplaced fracture (avulsion) of medial epicondyle of right humerus, initial encounter for closed fracture
S42444B	Nondisplaced fracture (avulsion) of medial epicondyle of right humerus, initial encounter for open fracture
S42445A	Nondisplaced fracture (avulsion) of medial epicondyle of left humerus, initial encounter for closed fracture
S42445B	Nondisplaced fracture (avulsion) of medial epicondyle of left humerus, initial encounter for open fracture
S42446A	Nondisplaced fracture (avulsion) of medial epicondyle of unspecified humerus, initial encounter for closed fracture
S42446B	Nondisplaced fracture (avulsion) of medial epicondyle of unspecified humerus, initial encounter for open fracture
S42447A	Incarcerated fracture (avulsion) of medial epicondyle of right humerus, initial encounter for closed fracture
S42447B	Incarcerated fracture (avulsion) of medial epicondyle of right humerus, initial encounter for open fracture
S42448A	Incarcerated fracture (avulsion) of medial epicondyle of left humerus, initial encounter for closed fracture
S42448B	Incarcerated fracture (avulsion) of medial epicondyle of left humerus, initial encounter for open fracture
S42449A	Incarcerated fracture (avulsion) of medial epicondyle of unspecified humerus, initial encounter for closed fracture
S42449B	Incarcerated fracture (avulsion) of medial epicondyle of unspecified humerus, initial encounter for open fracture
S42451A	Displaced fracture of lateral condyle of right humerus, initial encounter for closed fracture
S42451B	Displaced fracture of lateral condyle of right humerus, initial encounter for open fracture
S42452A	Displaced fracture of lateral condyle of left humerus, initial encounter for closed fracture
S42452B	Displaced fracture of lateral condyle of left humerus, initial encounter for open fracture
S42453A	Displaced fracture of lateral condyle of unspecified humerus, initial encounter for closed fracture
S42453B	Displaced fracture of lateral condyle of unspecified humerus, initial encounter for open fracture
S42454A	Nondisplaced fracture of lateral condyle of right humerus, initial encounter for closed fracture
S42454B	Nondisplaced fracture of lateral condyle of right humerus, initial encounter for open fracture
S42455A	Nondisplaced fracture of lateral condyle of left humerus, initial encounter for closed fracture
S42455B	Nondisplaced fracture of lateral condyle of left humerus, initial encounter for open fracture
S42456A	Nondisplaced fracture of lateral condyle of unspecified humerus, initial encounter for closed fracture
S42456B	Nondisplaced fracture of lateral condyle of unspecified humerus, initial encounter for open fracture
S42461A	Displaced fracture of medial condyle of right humerus, initial encounter for closed fracture
S42461B	Displaced fracture of medial condyle of right humerus, initial encounter for open fracture
S42462A	Displaced fracture of medial condyle of left humerus, initial encounter for closed fracture

S42462B	Displaced fracture of medial condyle of left humerus, initial encounter for open fracture
S42463A	Displaced fracture of medial condyle of unspecified humerus, initial encounter for closed fracture
S42463B	Displaced fracture of medial condyle of unspecified humerus, initial encounter for open fracture
S42464A	Nondisplaced fracture of medial condyle of right humerus, initial encounter for closed fracture
S42464B	Nondisplaced fracture of medial condyle of right humerus, initial encounter for open fracture
S42465A	Nondisplaced fracture of medial condyle of left humerus, initial encounter for closed fracture
S42465B	Nondisplaced fracture of medial condyle of left humerus, initial encounter for open fracture
S42466A	Nondisplaced fracture of medial condyle of unspecified humerus, initial encounter for closed fracture
S42466B	Nondisplaced fracture of medial condyle of unspecified humerus, initial encounter for open fracture
S42471A	Displaced transcondylar fracture of right humerus, initial encounter for closed fracture
S42471B	Displaced transcondylar fracture of right humerus, initial encounter for open fracture
S42472A	Displaced transcondylar fracture of left humerus, initial encounter for closed fracture
S42472B	Displaced transcondylar fracture of left humerus, initial encounter for open fracture
S42473A	Displaced transcondylar fracture of unspecified humerus, initial encounter for closed fracture
S42473B	Displaced transcondylar fracture of unspecified humerus, initial encounter for open fracture
S42474A	Nondisplaced transcondylar fracture of right humerus, initial encounter for closed fracture
S42474B	Nondisplaced transcondylar fracture of right humerus, initial encounter for open fracture
S42475A	Nondisplaced transcondylar fracture of left humerus, initial encounter for closed fracture
S42475B	Nondisplaced transcondylar fracture of left humerus, initial encounter for open fracture
S42476A	Nondisplaced transcondylar fracture of unspecified humerus, initial encounter for closed fracture
S42476B	Nondisplaced transcondylar fracture of unspecified humerus, initial encounter for open fracture
S42481A	Torus fracture of lower end of right humerus, initial encounter for closed fracture
S42482A	Torus fracture of lower end of left humerus, initial encounter for closed fracture
S42489A	Torus fracture of lower end of unspecified humerus, initial encounter for closed fracture
S42491A	Other displaced fracture of lower end of right humerus, initial encounter for closed fracture
S42491B	Other displaced fracture of lower end of right humerus, initial encounter for open fracture
S42492A	Other displaced fracture of lower end of left humerus, initial encounter for closed fracture
S42492B	Other displaced fracture of lower end of left humerus, initial encounter for open fracture
S42493A	Other displaced fracture of lower end of unspecified humerus, initial encounter for closed fracture
S42493B	Other displaced fracture of lower end of unspecified humerus, initial encounter for open fracture
S42494A	Other nondisplaced fracture of lower end of right humerus, initial encounter for closed fracture
S42494B	Other nondisplaced fracture of lower end of right humerus, initial encounter for open fracture
S42495A	Other nondisplaced fracture of lower end of left humerus, initial encounter for closed fracture
S42495B	Other nondisplaced fracture of lower end of left humerus, initial encounter for open fracture
S42496A	Other nondisplaced fracture of lower end of unspecified humerus, initial encounter for closed fracture
S42496B	Other nondisplaced fracture of lower end of unspecified humerus, initial encounter for open fracture
S4290XA	Fracture of unspecified shoulder girdle, part unspecified, initial encounter for closed fracture
S4290XB	Fracture of unspecified shoulder girdle, part unspecified, initial encounter for open fracture
S4291XA	Fracture of right shoulder girdle, part unspecified, initial encounter for closed fracture
S4291XB	Fracture of right shoulder girdle, part unspecified, initial encounter for open fracture
S4292XA	Fracture of left shoulder girdle, part unspecified, initial encounter for closed fracture
S4292XB	Fracture of left shoulder girdle, part unspecified, initial encounter for open fracture
S49001A	Unspecified physeal fracture of upper end of humerus, right arm, initial encounter for closed fracture

S49002A	Unspecified physeal fracture of upper end of humerus, left arm, initial encounter for closed fracture
S49009A	Unspecified physeal fracture of upper end of humerus, unspecified arm, initial encounter for closed fracture
S49011A	Salter-Harris Type I physeal fracture of upper end of humerus, right arm, initial encounter for closed fracture
S49012A	Salter-Harris Type I physeal fracture of upper end of humerus, left arm, initial encounter for closed fracture
S49019A	Salter-Harris Type I physeal fracture of upper end of humerus, unspecified arm, initial encounter for closed fracture
S49021A	Salter-Harris Type II physeal fracture of upper end of humerus, right arm, initial encounter for closed fracture
S49022A	Salter-Harris Type II physeal fracture of upper end of humerus, left arm, initial encounter for closed fracture
S49029A	Salter-Harris Type II physeal fracture of upper end of humerus, unspecified arm, initial encounter for closed fracture
S49031A	Salter Harris Type III physeal fracture of upper end of humerus, right arm, initial encounter for closed fracture
S49032A	Salter Harris Type III physeal fracture of upper end of humerus, left arm, initial encounter for closed fracture
S49039A	Salter Harris Type III physeal fracture of upper end of humerus, unspecified arm, initial encounter for closed fracture
S49041A	Salter-Harris Type IV physeal fracture of upper end of humerus, right arm, initial encounter for closed fracture
S49042A	Salter-Harris Type IV physeal fracture of upper end of humerus, left arm, initial encounter for closed fracture
S49049A	Salter-Harris Type IV physeal fracture of upper end of humerus, unspecified arm, initial encounter for closed fracture
S49091A	Other physeal fracture of upper end of humerus, right arm, initial encounter for closed fracture
S49092A	Other physeal fracture of upper end of humerus, left arm, initial encounter for closed fracture
S49099A	Other physeal fracture of upper end of humerus, unspecified arm, initial encounter for closed fracture
S49101A	Unspecified physeal fracture of lower end of humerus, right arm, initial encounter for closed fracture
S49102A	Unspecified physeal fracture of lower end of humerus, left arm, initial encounter for closed fracture
S49109A	Unspecified physeal fracture of lower end of humerus, unspecified arm, initial encounter for closed fracture
S49111A	Salter-Harris Type I physeal fracture of lower end of humerus, right arm, initial encounter for closed fracture
S49112A	Salter-Harris Type I physeal fracture of lower end of humerus, left arm, initial encounter for closed fracture
S49119A	Salter-Harris Type I physeal fracture of lower end of humerus, unspecified arm, initial encounter for closed fracture
S49121A	Salter-Harris Type II physeal fracture of lower end of humerus, right arm, initial encounter for closed fracture
S49122A	Salter-Harris Type II physeal fracture of lower end of humerus, left arm, initial encounter for closed fracture
S49129A	Salter-Harris Type II physeal fracture of lower end of humerus, unspecified arm, initial encounter for closed fracture
S49131A	Salter-Harris Type III physeal fracture of lower end of humerus, right arm, initial encounter for closed fracture
S49132A	Salter-Harris Type III physeal fracture of lower end of humerus, left arm, initial encounter for closed fracture

S49139A	Salter-Harris Type III physeal fracture of lower end of humerus, unspecified arm, initial encounter for closed fracture
S49141A	Salter-Harris Type IV physeal fracture of lower end of humerus, right arm, initial encounter for closed fracture
S49142A	Salter-Harris Type IV physeal fracture of lower end of humerus, left arm, initial encounter for closed fracture
S49149A	Salter-Harris Type IV physeal fracture of lower end of humerus, unspecified arm, initial encounter for closed fracture
S49191A	Other physeal fracture of lower end of humerus, right arm, initial encounter for closed fracture
S49192A	Other physeal fracture of lower end of humerus, left arm, initial encounter for closed fracture
S49199A	Other physeal fracture of lower end of humerus, unspecified arm, initial encounter for closed fracture
S52001A	Unspecified fracture of upper end of right ulna, initial encounter for closed fracture
S52001B	Unspecified fracture of upper end of right ulna, initial encounter for open fracture type I or II
S52001C	Unspecified fracture of upper end of right ulna, initial encounter for open fracture type IIIA, IIIB, or IIIC
S52002A	Unspecified fracture of upper end of left ulna, initial encounter for closed fracture
S52002B	Unspecified fracture of upper end of left ulna, initial encounter for open fracture type I or II
S52002C	Unspecified fracture of upper end of left ulna, initial encounter for open fracture type IIIA, IIIB, or IIIC
S52009A	Unspecified fracture of upper end of unspecified ulna, initial encounter for closed fracture
S52009B	Unspecified fracture of upper end of unspecified ulna, initial encounter for open fracture type I or II
S52009C	Unspecified fracture of upper end of unspecified ulna, initial encounter for open fracture type IIIA, IIIB, or IIIC
S52011A	Torus fracture of upper end of right ulna, initial encounter for closed fracture
S52012A	Torus fracture of upper end of left ulna, initial encounter for closed fracture
S52019A	Torus fracture of upper end of unspecified ulna, initial encounter for closed fracture
S52021A	Displaced fracture of olecranon process without intraarticular extension of right ulna, initial encounter for closed fracture
S52021B	Displaced fracture of olecranon process without intraarticular extension of right ulna, initial encounter for open fracture type I or II
S52021C	Displaced fracture of olecranon process without intraarticular extension of right ulna, initial encounter for open fracture type IIIA, IIIB, or IIIC
S52022A	Displaced fracture of olecranon process without intraarticular extension of left ulna, initial encounter for closed fracture
S52022B	Displaced fracture of olecranon process without intraarticular extension of left ulna, initial encounter for open fracture type I or II
S52022C	Displaced fracture of olecranon process without intraarticular extension of left ulna, initial encounter for open fracture type IIIA, IIIB, or IIIC
S52023A	Displaced fracture of olecranon process without intraarticular extension of unspecified ulna, initial encounter for closed fracture
S52023B	Displaced fracture of olecranon process without intraarticular extension of unspecified ulna, initial encounter for open fracture type I or II
S52023C	Displaced fracture of olecranon process without intraarticular extension of unspecified ulna, initial encounter for open fracture type IIIA, IIIB, or IIIC
S52024A	Nondisplaced fracture of olecranon process without intraarticular extension of right ulna, initial encounter for closed fracture
S52024B	Nondisplaced fracture of olecranon process without intraarticular extension of right ulna, initial encounter for open fracture type I or II
S52024C	Nondisplaced fracture of olecranon process without intraarticular extension of right ulna, initial encounter for open fracture type IIIA, IIIB, or IIIC
S52025A	Nondisplaced fracture of olecranon process without intraarticular extension of left ulna, initial

	encounter for closed fracture
S52025B	Nondisplaced fracture of olecranon process without intraarticular extension of left ulna, initial encounter for open fracture type I or II
S52025C	Nondisplaced fracture of olecranon process without intraarticular extension of left ulna, initial encounter for open fracture type IIIA, IIIB, or IIIC
S52026A	Nondisplaced fracture of olecranon process without intraarticular extension of unspecified ulna, initial encounter for closed fracture
S52026B	Nondisplaced fracture of olecranon process without intraarticular extension of unspecified ulna, initial encounter for open fracture type I or II
S52026C	Nondisplaced fracture of olecranon process without intraarticular extension of unspecified ulna, initial encounter for open fracture type IIIA, IIIB, or IIIC
S52031A	Displaced fracture of olecranon process with intraarticular extension of right ulna, initial encounter for closed fracture
S52031B	Displaced fracture of olecranon process with intraarticular extension of right ulna, initial encounter for open fracture type I or II
S52031C	Displaced fracture of olecranon process with intraarticular extension of right ulna, initial encounter for open fracture type IIIA, IIIB, or IIIC
S52032A	Displaced fracture of olecranon process with intraarticular extension of left ulna, initial encounter for closed fracture
S52032B	Displaced fracture of olecranon process with intraarticular extension of left ulna, initial encounter for open fracture type I or II
S52032C	Displaced fracture of olecranon process with intraarticular extension of left ulna, initial encounter for open fracture type IIIA, IIIB, or IIIC
S52033A	Displaced fracture of olecranon process with intraarticular extension of unspecified ulna, initial encounter for closed fracture
S52033B	Displaced fracture of olecranon process with intraarticular extension of unspecified ulna, initial encounter for open fracture type I or II
S52033C	Displaced fracture of olecranon process with intraarticular extension of unspecified ulna, initial encounter for open fracture type IIIA, IIIB, or IIIC
S52034A	Nondisplaced fracture of olecranon process with intraarticular extension of right ulna, initial encounter for closed fracture
S52034B	Nondisplaced fracture of olecranon process with intraarticular extension of right ulna, initial encounter for open fracture type I or II
S52034C	Nondisplaced fracture of olecranon process with intraarticular extension of right ulna, initial encounter for open fracture type IIIA, IIIB, or IIIC
S52035A	Nondisplaced fracture of olecranon process with intraarticular extension of left ulna, initial encounter for closed fracture
S52035B	Nondisplaced fracture of olecranon process with intraarticular extension of left ulna, initial encounter for open fracture type I or II
S52035C	Nondisplaced fracture of olecranon process with intraarticular extension of left ulna, initial encounter for open fracture type IIIA, IIIB, or IIIC
S52036A	Nondisplaced fracture of olecranon process with intraarticular extension of unspecified ulna, initial encounter for closed fracture
S52036B	Nondisplaced fracture of olecranon process with intraarticular extension of unspecified ulna, initial encounter for open fracture type I or II
S52036C	Nondisplaced fracture of olecranon process with intraarticular extension of unspecified ulna, initial encounter for open fracture type IIIA, IIIB, or IIIC
S52041A	Displaced fracture of coronoid process of right ulna, initial encounter for closed fracture
S52041B	Displaced fracture of coronoid process of right ulna, initial encounter for open fracture type I or II
S52041C	Displaced fracture of coronoid process of right ulna, initial encounter for open fracture type IIIA, IIIB, or IIIC

S52042A	Displaced fracture of coronoid process of left ulna, initial encounter for closed fracture
S52042B	Displaced fracture of coronoid process of left ulna, initial encounter for open fracture type I or II
S52042C	Displaced fracture of coronoid process of left ulna, initial encounter for open fracture type IIIA, IIIB, or IIIC
S52043A	Displaced fracture of coronoid process of unspecified ulna, initial encounter for closed fracture
S52043B	Displaced fracture of coronoid process of unspecified ulna, initial encounter for open fracture type I or II
S52043C	Displaced fracture of coronoid process of unspecified ulna, initial encounter for open fracture type IIIA, IIIB, or IIIC
S52044A	Nondisplaced fracture of coronoid process of right ulna, initial encounter for closed fracture
S52044B	Nondisplaced fracture of coronoid process of right ulna, initial encounter for open fracture type I or II
S52044C	Nondisplaced fracture of coronoid process of right ulna, initial encounter for open fracture type IIIA, IIIB, or IIIC
S52045A	Nondisplaced fracture of coronoid process of left ulna, initial encounter for closed fracture
S52045B	Nondisplaced fracture of coronoid process of left ulna, initial encounter for open fracture type I or II
S52045C	Nondisplaced fracture of coronoid process of left ulna, initial encounter for open fracture type IIIA, IIIB, or IIIC
S52046A	Nondisplaced fracture of coronoid process of unspecified ulna, initial encounter for closed fracture
S52046B	Nondisplaced fracture of coronoid process of unspecified ulna, initial encounter for open fracture type I or II
S52046C	Nondisplaced fracture of coronoid process of unspecified ulna, initial encounter for open fracture type IIIA, IIIB, or IIIC
S52091A	Other fracture of upper end of right ulna, initial encounter for closed fracture
S52091B	Other fracture of upper end of right ulna, initial encounter for open fracture type I or II
S52091C	Other fracture of upper end of right ulna, initial encounter for open fracture type IIIA, IIIB, or IIIC
S52092A	Other fracture of upper end of left ulna, initial encounter for closed fracture
S52092B	Other fracture of upper end of left ulna, initial encounter for open fracture type I or II
S52092C	Other fracture of upper end of left ulna, initial encounter for open fracture type IIIA, IIIB, or IIIC
S52099A	Other fracture of upper end of unspecified ulna, initial encounter for closed fracture
S52099B	Other fracture of upper end of unspecified ulna, initial encounter for open fracture type I or II
S52099C	Other fracture of upper end of unspecified ulna, initial encounter for open fracture type IIIA, IIIB, or IIIC
S52101A	Unspecified fracture of upper end of right radius, initial encounter for closed fracture
S52101B	Unspecified fracture of upper end of right radius, initial encounter for open fracture type I or II
S52101C	Unspecified fracture of upper end of right radius, initial encounter for open fracture type IIIA, IIIB, or IIIC
S52102A	Unspecified fracture of upper end of left radius, initial encounter for closed fracture
S52102B	Unspecified fracture of upper end of left radius, initial encounter for open fracture type I or II
S52102C	Unspecified fracture of upper end of left radius, initial encounter for open fracture type IIIA, IIIB, or IIIC
S52109A	Unspecified fracture of upper end of unspecified radius, initial encounter for closed fracture
S52109B	Unspecified fracture of upper end of unspecified radius, initial encounter for open fracture type I or II
S52109C	Unspecified fracture of upper end of unspecified radius, initial encounter for open fracture type IIIA, IIIB, or IIIC
S52111A	Torus fracture of upper end of right radius, initial encounter for closed fracture
S52112A	Torus fracture of upper end of left radius, initial encounter for closed fracture
S52119A	Torus fracture of upper end of unspecified radius, initial encounter for closed fracture
S52121A	Displaced fracture of head of right radius, initial encounter for closed fracture

S52121B	Displaced fracture of head of right radius, initial encounter for open fracture type I or II
S52121C	Displaced fracture of head of right radius, initial encounter for open fracture type IIIA, IIIB, or IIIC
S52122A	Displaced fracture of head of left radius, initial encounter for closed fracture
S52122B	Displaced fracture of head of left radius, initial encounter for open fracture type I or II
S52122C	Displaced fracture of head of left radius, initial encounter for open fracture type IIIA, IIIB, or IIIC
S52123A	Displaced fracture of head of unspecified radius, initial encounter for closed fracture
S52123B	Displaced fracture of head of unspecified radius, initial encounter for open fracture type I or II
S52123C	Displaced fracture of head of unspecified radius, initial encounter for open fracture type IIIA, IIIB, or IIIC
S52124A	Nondisplaced fracture of head of right radius, initial encounter for closed fracture
S52124B	Nondisplaced fracture of head of right radius, initial encounter for open fracture type I or II
S52124C	Nondisplaced fracture of head of right radius, initial encounter for open fracture type IIIA, IIIB, or IIIC
S52125A	Nondisplaced fracture of head of left radius, initial encounter for closed fracture
S52125B	Nondisplaced fracture of head of left radius, initial encounter for open fracture type I or II
S52125C	Nondisplaced fracture of head of left radius, initial encounter for open fracture type IIIA, IIIB, or IIIC
S52126A	Nondisplaced fracture of head of unspecified radius, initial encounter for closed fracture
S52126B	Nondisplaced fracture of head of unspecified radius, initial encounter for open fracture type I or II
S52126C	Nondisplaced fracture of head of unspecified radius, initial encounter for open fracture type IIIA, IIIB, or IIIC
S52131A	Displaced fracture of neck of right radius, initial encounter for closed fracture
S52131B	Displaced fracture of neck of right radius, initial encounter for open fracture type I or II
S52131C	Displaced fracture of neck of right radius, initial encounter for open fracture type IIIA, IIIB, or IIIC
S52132A	Displaced fracture of neck of left radius, initial encounter for closed fracture
S52132B	Displaced fracture of neck of left radius, initial encounter for open fracture type I or II
S52132C	Displaced fracture of neck of left radius, initial encounter for open fracture type IIIA, IIIB, or IIIC
S52133A	Displaced fracture of neck of unspecified radius, initial encounter for closed fracture
S52133B	Displaced fracture of neck of unspecified radius, initial encounter for open fracture type I or II
S52133C	Displaced fracture of neck of unspecified radius, initial encounter for open fracture type IIIA, IIIB, or IIIC
S52134A	Nondisplaced fracture of neck of right radius, initial encounter for closed fracture
S52134B	Nondisplaced fracture of neck of right radius, initial encounter for open fracture type I or II
S52134C	Nondisplaced fracture of neck of right radius, initial encounter for open fracture type IIIA, IIIB, or IIIC
S52135A	Nondisplaced fracture of neck of left radius, initial encounter for closed fracture
S52135B	Nondisplaced fracture of neck of left radius, initial encounter for open fracture type I or II
S52135C	Nondisplaced fracture of neck of left radius, initial encounter for open fracture type IIIA, IIIB, or IIIC
S52136A	Nondisplaced fracture of neck of unspecified radius, initial encounter for closed fracture
S52136B	Nondisplaced fracture of neck of unspecified radius, initial encounter for open fracture type I or II
S52136C	Nondisplaced fracture of neck of unspecified radius, initial encounter for open fracture type IIIA, IIIB, or IIIC
S52181A	Other fracture of upper end of right radius, initial encounter for closed fracture
S52181B	Other fracture of upper end of right radius, initial encounter for open fracture type I or II
S52181C	Other fracture of upper end of right radius, initial encounter for open fracture type IIIA, IIIB, or IIIC
S52182A	Other fracture of upper end of left radius, initial encounter for closed fracture
S52182B	Other fracture of upper end of left radius, initial encounter for open fracture type I or II
S52182C	Other fracture of upper end of left radius, initial encounter for open fracture type IIIA, IIIB, or IIIC

S52189A	Other fracture of upper end of unspecified radius, initial encounter for closed fracture
S52189B	Other fracture of upper end of unspecified radius, initial encounter for open fracture type I or II
S52189C	Other fracture of upper end of unspecified radius, initial encounter for open fracture type IIIA, IIIB, or IIIC
S52201A	Unspecified fracture of shaft of right ulna, initial encounter for closed fracture
S52201B	Unspecified fracture of shaft of right ulna, initial encounter for open fracture type I or II
S52201C	Unspecified fracture of shaft of right ulna, initial encounter for open fracture type IIIA, IIIB, or IIIC
S52202A	Unspecified fracture of shaft of left ulna, initial encounter for closed fracture
S52202B	Unspecified fracture of shaft of left ulna, initial encounter for open fracture type I or II
S52202C	Unspecified fracture of shaft of left ulna, initial encounter for open fracture type IIIA, IIIB, or IIIC
S52209A	Unspecified fracture of shaft of unspecified ulna, initial encounter for closed fracture
S52209B	Unspecified fracture of shaft of unspecified ulna, initial encounter for open fracture type I or II
S52209C	Unspecified fracture of shaft of unspecified ulna, initial encounter for open fracture type IIIA, IIIB, or IIIC
S52211A	Greenstick fracture of shaft of right ulna, initial encounter for closed fracture
S52212A	Greenstick fracture of shaft of left ulna, initial encounter for closed fracture
S52219A	Greenstick fracture of shaft of unspecified ulna, initial encounter for closed fracture
S52221A	Displaced transverse fracture of shaft of right ulna, initial encounter for closed fracture
S52221B	Displaced transverse fracture of shaft of right ulna, initial encounter for open fracture type I or II
S52221C	Displaced transverse fracture of shaft of right ulna, initial encounter for open fracture type IIIA, IIIB, or IIIC
S52222A	Displaced transverse fracture of shaft of left ulna, initial encounter for closed fracture
S52222B	Displaced transverse fracture of shaft of left ulna, initial encounter for open fracture type I or II
S52222C	Displaced transverse fracture of shaft of left ulna, initial encounter for open fracture type IIIA, IIIB, or IIIC
S52223A	Displaced transverse fracture of shaft of unspecified ulna, initial encounter for closed fracture
S52223B	Displaced transverse fracture of shaft of unspecified ulna, initial encounter for open fracture type I or II
S52223C	Displaced transverse fracture of shaft of unspecified ulna, initial encounter for open fracture type IIIA, IIIB, or IIIC
S52224A	Nondisplaced transverse fracture of shaft of right ulna, initial encounter for closed fracture
S52224B	Nondisplaced transverse fracture of shaft of right ulna, initial encounter for open fracture type I or II
S52224C	Nondisplaced transverse fracture of shaft of right ulna, initial encounter for open fracture type IIIA, IIIB, or IIIC
S52225A	Nondisplaced transverse fracture of shaft of left ulna, initial encounter for closed fracture
S52225B	Nondisplaced transverse fracture of shaft of left ulna, initial encounter for open fracture type I or II
S52225C	Nondisplaced transverse fracture of shaft of left ulna, initial encounter for open fracture type IIIA, IIIB, or IIIC
S52226A	Nondisplaced transverse fracture of shaft of unspecified ulna, initial encounter for closed fracture
S52226B	Nondisplaced transverse fracture of shaft of unspecified ulna, initial encounter for open fracture type I or II
S52226C	Nondisplaced transverse fracture of shaft of unspecified ulna, initial encounter for open fracture type IIIA, IIIB, or IIIC
S52231A	Displaced oblique fracture of shaft of right ulna, initial encounter for closed fracture
S52231B	Displaced oblique fracture of shaft of right ulna, initial encounter for open fracture type I or II
S52231C	Displaced oblique fracture of shaft of right ulna, initial encounter for open fracture type IIIA, IIIB, or IIIC
S52232A	Displaced oblique fracture of shaft of left ulna, initial encounter for closed fracture

S52232B	Displaced oblique fracture of shaft of left ulna, initial encounter for open fracture type I or II
S52232C	Displaced oblique fracture of shaft of left ulna, initial encounter for open fracture type IIIA, IIIB, or IIIC
S52233A	Displaced oblique fracture of shaft of unspecified ulna, initial encounter for closed fracture
S52233B	Displaced oblique fracture of shaft of unspecified ulna, initial encounter for open fracture type I or II
S52233C	Displaced oblique fracture of shaft of unspecified ulna, initial encounter for open fracture type IIIA, IIIB, or IIIC
S52234A	Nondisplaced oblique fracture of shaft of right ulna, initial encounter for closed fracture
S52234B	Nondisplaced oblique fracture of shaft of right ulna, initial encounter for open fracture type I or II
S52234C	Nondisplaced oblique fracture of shaft of right ulna, initial encounter for open fracture type IIIA, IIIB, or IIIC
S52235A	Nondisplaced oblique fracture of shaft of left ulna, initial encounter for closed fracture
S52235B	Nondisplaced oblique fracture of shaft of left ulna, initial encounter for open fracture type I or II
S52235C	Nondisplaced oblique fracture of shaft of left ulna, initial encounter for open fracture type IIIA, IIIB, or IIIC
S52236A	Nondisplaced oblique fracture of shaft of unspecified ulna, initial encounter for closed fracture
S52236B	Nondisplaced oblique fracture of shaft of unspecified ulna, initial encounter for open fracture type I or II
S52236C	Nondisplaced oblique fracture of shaft of unspecified ulna, initial encounter for open fracture type IIIA, IIIB, or IIIC
S52241A	Displaced spiral fracture of shaft of ulna, right arm, initial encounter for closed fracture
S52241B	Displaced spiral fracture of shaft of ulna, right arm, initial encounter for open fracture type I or II
S52241C	Displaced spiral fracture of shaft of ulna, right arm, initial encounter for open fracture type IIIA, IIIB, or IIIC
S52242A	Displaced spiral fracture of shaft of ulna, left arm, initial encounter for closed fracture
S52242B	Displaced spiral fracture of shaft of ulna, left arm, initial encounter for open fracture type I or II
S52242C	Displaced spiral fracture of shaft of ulna, left arm, initial encounter for open fracture type IIIA, IIIB, or IIIC
S52243A	Displaced spiral fracture of shaft of ulna, unspecified arm, initial encounter for closed fracture
S52243B	Displaced spiral fracture of shaft of ulna, unspecified arm, initial encounter for open fracture type I or II
S52243C	Displaced spiral fracture of shaft of ulna, unspecified arm, initial encounter for open fracture type IIIA, IIIB, or IIIC
S52244A	Nondisplaced spiral fracture of shaft of ulna, right arm, initial encounter for closed fracture
S52244B	Nondisplaced spiral fracture of shaft of ulna, right arm, initial encounter for open fracture type I or II
S52244C	Nondisplaced spiral fracture of shaft of ulna, right arm, initial encounter for open fracture type IIIA, IIIB, or IIIC
S52245A	Nondisplaced spiral fracture of shaft of ulna, left arm, initial encounter for closed fracture
S52245B	Nondisplaced spiral fracture of shaft of ulna, left arm, initial encounter for open fracture type I or II
S52245C	Nondisplaced spiral fracture of shaft of ulna, left arm, initial encounter for open fracture type IIIA, IIIB, or IIIC
S52246A	Nondisplaced spiral fracture of shaft of ulna, unspecified arm, initial encounter for closed fracture
S52246B	Nondisplaced spiral fracture of shaft of ulna, unspecified arm, initial encounter for open fracture type I or II
S52246C	Nondisplaced spiral fracture of shaft of ulna, unspecified arm, initial encounter for open fracture type IIIA, IIIB, or IIIC
S52251A	Displaced comminuted fracture of shaft of ulna, right arm, initial encounter for closed fracture
S52251B	Displaced comminuted fracture of shaft of ulna, right arm, initial encounter for open fracture type I or II

S52251C	Displaced comminuted fracture of shaft of ulna, right arm, initial encounter for open fracture type IIIA, IIIB, or IIIC
S52252A	Displaced comminuted fracture of shaft of ulna, left arm, initial encounter for closed fracture
S52252B	Displaced comminuted fracture of shaft of ulna, left arm, initial encounter for open fracture type I or II
S52252C	Displaced comminuted fracture of shaft of ulna, left arm, initial encounter for open fracture type IIIA, IIIB, or IIIC
S52253A	Displaced comminuted fracture of shaft of ulna, unspecified arm, initial encounter for closed fracture
S52253B	Displaced comminuted fracture of shaft of ulna, unspecified arm, initial encounter for open fracture type I or II
S52253C	Displaced comminuted fracture of shaft of ulna, unspecified arm, initial encounter for open fracture type IIIA, IIIB, or IIIC
S52254A	Nondisplaced comminuted fracture of shaft of ulna, right arm, initial encounter for closed fracture
S52254B	Nondisplaced comminuted fracture of shaft of ulna, right arm, initial encounter for open fracture type I or II
S52254C	Nondisplaced comminuted fracture of shaft of ulna, right arm, initial encounter for open fracture type IIIA, IIIB, or IIIC
S52255A	Nondisplaced comminuted fracture of shaft of ulna, left arm, initial encounter for closed fracture
S52255B	Nondisplaced comminuted fracture of shaft of ulna, left arm, initial encounter for open fracture type I or II
S52255C	Nondisplaced comminuted fracture of shaft of ulna, left arm, initial encounter for open fracture type IIIA, IIIB, or IIIC
S52256A	Nondisplaced comminuted fracture of shaft of ulna, unspecified arm, initial encounter for closed fracture
S52256B	Nondisplaced comminuted fracture of shaft of ulna, unspecified arm, initial encounter for open fracture type I or II
S52256C	Nondisplaced comminuted fracture of shaft of ulna, unspecified arm, initial encounter for open fracture type IIIA, IIIB, or IIIC
S52261A	Displaced segmental fracture of shaft of ulna, right arm, initial encounter for closed fracture
S52261B	Displaced segmental fracture of shaft of ulna, right arm, initial encounter for open fracture type I or II
S52261C	Displaced segmental fracture of shaft of ulna, right arm, initial encounter for open fracture type IIIA, IIIB, or IIIC
S52262A	Displaced segmental fracture of shaft of ulna, left arm, initial encounter for closed fracture
S52262B	Displaced segmental fracture of shaft of ulna, left arm, initial encounter for open fracture type I or II
S52262C	Displaced segmental fracture of shaft of ulna, left arm, initial encounter for open fracture type IIIA, IIIB, or IIIC
S52263A	Displaced segmental fracture of shaft of ulna, unspecified arm, initial encounter for closed fracture
S52263B	Displaced segmental fracture of shaft of ulna, unspecified arm, initial encounter for open fracture type I or II
S52263C	Displaced segmental fracture of shaft of ulna, unspecified arm, initial encounter for open fracture type IIIA, IIIB, or IIIC
S52264A	Nondisplaced segmental fracture of shaft of ulna, right arm, initial encounter for closed fracture
S52264B	Nondisplaced segmental fracture of shaft of ulna, right arm, initial encounter for open fracture type I or II
S52264C	Nondisplaced segmental fracture of shaft of ulna, right arm, initial encounter for open fracture type IIIA, IIIB, or IIIC
S52265A	Nondisplaced segmental fracture of shaft of ulna, left arm, initial encounter for closed fracture
S52265B	Nondisplaced segmental fracture of shaft of ulna, left arm, initial encounter for open fracture type I or II
S52265C	Nondisplaced segmental fracture of shaft of ulna, left arm, initial encounter for open fracture type IIIA,

	IIIB, or IIIC
S52266A	Nondisplaced segmental fracture of shaft of ulna, unspecified arm, initial encounter for closed fracture
S52266B	Nondisplaced segmental fracture of shaft of ulna, unspecified arm, initial encounter for open fracture type I or II
S52266C	Nondisplaced segmental fracture of shaft of ulna, unspecified arm, initial encounter for open fracture type IIIA, IIIB, or IIIC
S52271A	Monteggia's fracture of right ulna, initial encounter for closed fracture
S52271B	Monteggia's fracture of right ulna, initial encounter for open fracture type I or II
S52271C	Monteggia's fracture of right ulna, initial encounter for open fracture type IIIA, IIIB, or IIIC
S52272A	Monteggia's fracture of left ulna, initial encounter for closed fracture
S52272B	Monteggia's fracture of left ulna, initial encounter for open fracture type I or II
S52272C	Monteggia's fracture of left ulna, initial encounter for open fracture type IIIA, IIIB, or IIIC
S52279A	Monteggia's fracture of unspecified ulna, initial encounter for closed fracture
S52279B	Monteggia's fracture of unspecified ulna, initial encounter for open fracture type I or II
S52279C	Monteggia's fracture of unspecified ulna, initial encounter for open fracture type IIIA, IIIB, or IIIC
S52281A	Bent bone of right ulna, initial encounter for closed fracture
S52281B	Bent bone of right ulna, initial encounter for open fracture type I or II
S52281C	Bent bone of right ulna, initial encounter for open fracture type IIIA, IIIB, or IIIC
S52282A	Bent bone of left ulna, initial encounter for closed fracture
S52282B	Bent bone of left ulna, initial encounter for open fracture type I or II
S52282C	Bent bone of left ulna, initial encounter for open fracture type IIIA, IIIB, or IIIC
S52283A	Bent bone of unspecified ulna, initial encounter for closed fracture
S52283B	Bent bone of unspecified ulna, initial encounter for open fracture type I or II
S52283C	Bent bone of unspecified ulna, initial encounter for open fracture type IIIA, IIIB, or IIIC
S52291A	Other fracture of shaft of right ulna, initial encounter for closed fracture
S52291B	Other fracture of shaft of right ulna, initial encounter for open fracture type I or II
S52291C	Other fracture of shaft of right ulna, initial encounter for open fracture type IIIA, IIIB, or IIIC
S52292A	Other fracture of shaft of left ulna, initial encounter for closed fracture
S52292B	Other fracture of shaft of left ulna, initial encounter for open fracture type I or II
S52292C	Other fracture of shaft of left ulna, initial encounter for open fracture type IIIA, IIIB, or IIIC
S52299A	Other fracture of shaft of unspecified ulna, initial encounter for closed fracture
S52299B	Other fracture of shaft of unspecified ulna, initial encounter for open fracture type I or II
S52299C	Other fracture of shaft of unspecified ulna, initial encounter for open fracture type IIIA, IIIB, or IIIC
S52301A	Unspecified fracture of shaft of right radius, initial encounter for closed fracture
S52301B	Unspecified fracture of shaft of right radius, initial encounter for open fracture type I or II
S52301C	Unspecified fracture of shaft of right radius, initial encounter for open fracture type IIIA, IIIB, or IIIC
S52302A	Unspecified fracture of shaft of left radius, initial encounter for closed fracture
S52302B	Unspecified fracture of shaft of left radius, initial encounter for open fracture type I or II
S52302C	Unspecified fracture of shaft of left radius, initial encounter for open fracture type IIIA, IIIB, or IIIC
S52309A	Unspecified fracture of shaft of unspecified radius, initial encounter for closed fracture
S52309B	Unspecified fracture of shaft of unspecified radius, initial encounter for open fracture type I or II
S52309C	Unspecified fracture of shaft of unspecified radius, initial encounter for open fracture type IIIA, IIIB, or IIIC
S52311A	Greenstick fracture of shaft of radius, right arm, initial encounter for closed fracture

S52312A	Greenstick fracture of shaft of radius, left arm, initial encounter for closed fracture
S52319A	Greenstick fracture of shaft of radius, unspecified arm, initial encounter for closed fracture
S52321A	Displaced transverse fracture of shaft of right radius, initial encounter for closed fracture
S52321B	Displaced transverse fracture of shaft of right radius, initial encounter for open fracture type I or II
S52321C	Displaced transverse fracture of shaft of right radius, initial encounter for open fracture type IIIA, IIIB, or IIIC
S52322A	Displaced transverse fracture of shaft of left radius, initial encounter for closed fracture
S52322B	Displaced transverse fracture of shaft of left radius, initial encounter for open fracture type I or II
S52322C	Displaced transverse fracture of shaft of left radius, initial encounter for open fracture type IIIA, IIIB, or IIIC
S52323A	Displaced transverse fracture of shaft of unspecified radius, initial encounter for closed fracture
S52323B	Displaced transverse fracture of shaft of unspecified radius, initial encounter for open fracture type I or II
S52323C	Displaced transverse fracture of shaft of unspecified radius, initial encounter for open fracture type IIIA, IIIB, or IIIC
S52324A	Nondisplaced transverse fracture of shaft of right radius, initial encounter for closed fracture
S52324B	Nondisplaced transverse fracture of shaft of right radius, initial encounter for open fracture type I or II
S52324C	Nondisplaced transverse fracture of shaft of right radius, initial encounter for open fracture type IIIA, IIIB, or IIIC
S52325A	Nondisplaced transverse fracture of shaft of left radius, initial encounter for closed fracture
S52325B	Nondisplaced transverse fracture of shaft of left radius, initial encounter for open fracture type I or II
S52325C	Nondisplaced transverse fracture of shaft of left radius, initial encounter for open fracture type IIIA, IIIB, or IIIC
S52326A	Nondisplaced transverse fracture of shaft of unspecified radius, initial encounter for closed fracture
S52326B	Nondisplaced transverse fracture of shaft of unspecified radius, initial encounter for open fracture type I or II
S52326C	Nondisplaced transverse fracture of shaft of unspecified radius, initial encounter for open fracture type IIIA, IIIB, or IIIC
S52331A	Displaced oblique fracture of shaft of right radius, initial encounter for closed fracture
S52331B	Displaced oblique fracture of shaft of right radius, initial encounter for open fracture type I or II
S52331C	Displaced oblique fracture of shaft of right radius, initial encounter for open fracture type IIIA, IIIB, or IIIC
S52332A	Displaced oblique fracture of shaft of left radius, initial encounter for closed fracture
S52332B	Displaced oblique fracture of shaft of left radius, initial encounter for open fracture type I or II
S52332C	Displaced oblique fracture of shaft of left radius, initial encounter for open fracture type IIIA, IIIB, or IIIC
S52333A	Displaced oblique fracture of shaft of unspecified radius, initial encounter for closed fracture
S52333B	Displaced oblique fracture of shaft of unspecified radius, initial encounter for open fracture type I or II
S52333C	Displaced oblique fracture of shaft of unspecified radius, initial encounter for open fracture type IIIA, IIIB, or IIIC
S52334A	Nondisplaced oblique fracture of shaft of right radius, initial encounter for closed fracture
S52334B	Nondisplaced oblique fracture of shaft of right radius, initial encounter for open fracture type I or II
S52334C	Nondisplaced oblique fracture of shaft of right radius, initial encounter for open fracture type IIIA, IIIB, or IIIC
S52335A	Nondisplaced oblique fracture of shaft of left radius, initial encounter for closed fracture
S52335B	Nondisplaced oblique fracture of shaft of left radius, initial encounter for open fracture type I or II
S52335C	Nondisplaced oblique fracture of shaft of left radius, initial encounter for open fracture type IIIA, IIIB,

	or IIIC
S52336A	Nondisplaced oblique fracture of shaft of unspecified radius, initial encounter for closed fracture
S52336B	Nondisplaced oblique fracture of shaft of unspecified radius, initial encounter for open fracture type I or II
S52336C	Nondisplaced oblique fracture of shaft of unspecified radius, initial encounter for open fracture type IIIA, IIIB, or IIIC
S52341A	Displaced spiral fracture of shaft of radius, right arm, initial encounter for closed fracture
S52341B	Displaced spiral fracture of shaft of radius, right arm, initial encounter for open fracture type I or II
S52341C	Displaced spiral fracture of shaft of radius, right arm, initial encounter for open fracture type IIIA, IIIB, or IIIC
S52342A	Displaced spiral fracture of shaft of radius, left arm, initial encounter for closed fracture
S52342B	Displaced spiral fracture of shaft of radius, left arm, initial encounter for open fracture type I or II
S52342C	Displaced spiral fracture of shaft of radius, left arm, initial encounter for open fracture type IIIA, IIIB, or IIIC
S52343A	Displaced spiral fracture of shaft of radius, unspecified arm, initial encounter for closed fracture
S52343B	Displaced spiral fracture of shaft of radius, unspecified arm, initial encounter for open fracture type I or II
S52343C	Displaced spiral fracture of shaft of radius, unspecified arm, initial encounter for open fracture type IIIA, IIIB, or IIIC
S52344A	Nondisplaced spiral fracture of shaft of radius, right arm, initial encounter for closed fracture
S52344B	Nondisplaced spiral fracture of shaft of radius, right arm, initial encounter for open fracture type I or II
S52344C	Nondisplaced spiral fracture of shaft of radius, right arm, initial encounter for open fracture type IIIA, IIIB, or IIIC
S52345A	Nondisplaced spiral fracture of shaft of radius, left arm, initial encounter for closed fracture
S52345B	Nondisplaced spiral fracture of shaft of radius, left arm, initial encounter for open fracture type I or II
S52345C	Nondisplaced spiral fracture of shaft of radius, left arm, initial encounter for open fracture type IIIA, IIIB, or IIIC
S52346A	Nondisplaced spiral fracture of shaft of radius, unspecified arm, initial encounter for closed fracture
S52346B	Nondisplaced spiral fracture of shaft of radius, unspecified arm, initial encounter for open fracture type I or II
S52346C	Nondisplaced spiral fracture of shaft of radius, unspecified arm, initial encounter for open fracture type IIIA, IIIB, or IIIC
S52351A	Displaced comminuted fracture of shaft of radius, right arm, initial encounter for closed fracture
S52351B	Displaced comminuted fracture of shaft of radius, right arm, initial encounter for open fracture type I or II
S52351C	Displaced comminuted fracture of shaft of radius, right arm, initial encounter for open fracture type IIIA, IIIB, or IIIC
S52352A	Displaced comminuted fracture of shaft of radius, left arm, initial encounter for closed fracture
S52352B	Displaced comminuted fracture of shaft of radius, left arm, initial encounter for open fracture type I or II
S52352C	Displaced comminuted fracture of shaft of radius, left arm, initial encounter for open fracture type IIIA, IIIB, or IIIC
S52353A	Displaced comminuted fracture of shaft of radius, unspecified arm, initial encounter for closed fracture
S52353B	Displaced comminuted fracture of shaft of radius, unspecified arm, initial encounter for open fracture type I or II
S52353C	Displaced comminuted fracture of shaft of radius, unspecified arm, initial encounter for open fracture type IIIA, IIIB, or IIIC
S52354A	Nondisplaced comminuted fracture of shaft of radius, right arm, initial encounter for closed fracture

S52354B	Nondisplaced comminuted fracture of shaft of radius, right arm, initial encounter for open fracture type I or II
S52354C	Nondisplaced comminuted fracture of shaft of radius, right arm, initial encounter for open fracture type IIIA, IIIB, or IIIC
S52355A	Nondisplaced comminuted fracture of shaft of radius, left arm, initial encounter for closed fracture
S52355B	Nondisplaced comminuted fracture of shaft of radius, left arm, initial encounter for open fracture type I or II
S52355C	Nondisplaced comminuted fracture of shaft of radius, left arm, initial encounter for open fracture type IIIA, IIIB, or IIIC
S52356A	Nondisplaced comminuted fracture of shaft of radius, unspecified arm, initial encounter for closed fracture
S52356B	Nondisplaced comminuted fracture of shaft of radius, unspecified arm, initial encounter for open fracture type I or II
S52356C	Nondisplaced comminuted fracture of shaft of radius, unspecified arm, initial encounter for open fracture type IIIA, IIIB, or IIIC
S52361A	Displaced segmental fracture of shaft of radius, right arm, initial encounter for closed fracture
S52361B	Displaced segmental fracture of shaft of radius, right arm, initial encounter for open fracture type I or II
S52361C	Displaced segmental fracture of shaft of radius, right arm, initial encounter for open fracture type IIIA, IIIB, or IIIC
S52362A	Displaced segmental fracture of shaft of radius, left arm, initial encounter for closed fracture
S52362B	Displaced segmental fracture of shaft of radius, left arm, initial encounter for open fracture type I or II
S52362C	Displaced segmental fracture of shaft of radius, left arm, initial encounter for open fracture type IIIA, IIIB, or IIIC
S52363A	Displaced segmental fracture of shaft of radius, unspecified arm, initial encounter for closed fracture
S52363B	Displaced segmental fracture of shaft of radius, unspecified arm, initial encounter for open fracture type I or II
S52363C	Displaced segmental fracture of shaft of radius, unspecified arm, initial encounter for open fracture type IIIA, IIIB, or IIIC
S52364A	Nondisplaced segmental fracture of shaft of radius, right arm, initial encounter for closed fracture
S52364B	Nondisplaced segmental fracture of shaft of radius, right arm, initial encounter for open fracture type I or II
S52364C	Nondisplaced segmental fracture of shaft of radius, right arm, initial encounter for open fracture type IIIA, IIIB, or IIIC
S52365A	Nondisplaced segmental fracture of shaft of radius, left arm, initial encounter for closed fracture
S52365B	Nondisplaced segmental fracture of shaft of radius, left arm, initial encounter for open fracture type I or II
S52365C	Nondisplaced segmental fracture of shaft of radius, left arm, initial encounter for open fracture type IIIA, IIIB, or IIIC
S52366A	Nondisplaced segmental fracture of shaft of radius, unspecified arm, initial encounter for closed fracture
S52366B	Nondisplaced segmental fracture of shaft of radius, unspecified arm, initial encounter for open fracture type I or II
S52366C	Nondisplaced segmental fracture of shaft of radius, unspecified arm, initial encounter for open fracture type IIIA, IIIB, or IIIC
S52371A	Galeazzi's fracture of right radius, initial encounter for closed fracture
S52371B	Galeazzi's fracture of right radius, initial encounter for open fracture type I or II
S52371C	Galeazzi's fracture of right radius, initial encounter for open fracture type IIIA, IIIB, or IIIC
S52372A	Galeazzi's fracture of left radius, initial encounter for closed fracture

S52372B	Galeazzi's fracture of left radius, initial encounter for open fracture type I or II
S52372C	Galeazzi's fracture of left radius, initial encounter for open fracture type IIIA, IIIB, or IIIC
S52379A	Galeazzi's fracture of unspecified radius, initial encounter for closed fracture
S52379B	Galeazzi's fracture of unspecified radius, initial encounter for open fracture type I or II
S52379C	Galeazzi's fracture of unspecified radius, initial encounter for open fracture type IIIA, IIIB, or IIIC
S52381A	Bent bone of right radius, initial encounter for closed fracture
S52381B	Bent bone of right radius, initial encounter for open fracture type I or II
S52381C	Bent bone of right radius, initial encounter for open fracture type IIIA, IIIB, or IIIC
S52382A	Bent bone of left radius, initial encounter for closed fracture
S52382B	Bent bone of left radius, initial encounter for open fracture type I or II
S52382C	Bent bone of left radius, initial encounter for open fracture type IIIA, IIIB, or IIIC
S52389A	Bent bone of unspecified radius, initial encounter for closed fracture
S52389B	Bent bone of unspecified radius, initial encounter for open fracture type I or II
S52389C	Bent bone of unspecified radius, initial encounter for open fracture type IIIA, IIIB, or IIIC
S52391A	Other fracture of shaft of radius, right arm, initial encounter for closed fracture
S52391B	Other fracture of shaft of radius, right arm, initial encounter for open fracture type I or II
S52391C	Other fracture of shaft of radius, right arm, initial encounter for open fracture type IIIA, IIIB, or IIIC
S52392A	Other fracture of shaft of radius, left arm, initial encounter for closed fracture
S52392B	Other fracture of shaft of radius, left arm, initial encounter for open fracture type I or II
S52392C	Other fracture of shaft of radius, left arm, initial encounter for open fracture type IIIA, IIIB, or IIIC
S52399A	Other fracture of shaft of radius, unspecified arm, initial encounter for closed fracture
S52399B	Other fracture of shaft of radius, unspecified arm, initial encounter for open fracture type I or II
S52399C	Other fracture of shaft of radius, unspecified arm, initial encounter for open fracture type IIIA, IIIB, or IIIC
S52501A	Unspecified fracture of the lower end of right radius, initial encounter for closed fracture
S52501B	Unspecified fracture of the lower end of right radius, initial encounter for open fracture type I or II
S52501C	Unspecified fracture of the lower end of right radius, initial encounter for open fracture type IIIA, IIIB, or IIIC
S52502A	Unspecified fracture of the lower end of left radius, initial encounter for closed fracture
S52502B	Unspecified fracture of the lower end of left radius, initial encounter for open fracture type I or II
S52502C	Unspecified fracture of the lower end of left radius, initial encounter for open fracture type IIIA, IIIB, or IIIC
S52509A	Unspecified fracture of the lower end of unspecified radius, initial encounter for closed fracture
S52509B	Unspecified fracture of the lower end of unspecified radius, initial encounter for open fracture type I or II
S52509C	Unspecified fracture of the lower end of unspecified radius, initial encounter for open fracture type IIIA, IIIB, or IIIC
S52511A	Displaced fracture of right radial styloid process, initial encounter for closed fracture
S52511B	Displaced fracture of right radial styloid process, initial encounter for open fracture type I or II
S52511C	Displaced fracture of right radial styloid process, initial encounter for open fracture type IIIA, IIIB, or IIIC
S52512A	Displaced fracture of left radial styloid process, initial encounter for closed fracture
S52512B	Displaced fracture of left radial styloid process, initial encounter for open fracture type I or II
S52512C	Displaced fracture of left radial styloid process, initial encounter for open fracture type IIIA, IIIB, or IIIC
S52513A	Displaced fracture of unspecified radial styloid process, initial encounter for closed fracture

S52513B	Displaced fracture of unspecified radial styloid process, initial encounter for open fracture type I or II
S52513C	Displaced fracture of unspecified radial styloid process, initial encounter for open fracture type IIIA, IIIB, or IIIC
S52514A	Nondisplaced fracture of right radial styloid process, initial encounter for closed fracture
S52514B	Nondisplaced fracture of right radial styloid process, initial encounter for open fracture type I or II
S52514C	Nondisplaced fracture of right radial styloid process, initial encounter for open fracture type IIIA, IIIB, or IIIC
S52515A	Nondisplaced fracture of left radial styloid process, initial encounter for closed fracture
S52515B	Nondisplaced fracture of left radial styloid process, initial encounter for open fracture type I or II
S52515C	Nondisplaced fracture of left radial styloid process, initial encounter for open fracture type IIIA, IIIB, or IIIC
S52516A	Nondisplaced fracture of unspecified radial styloid process, initial encounter for closed fracture
S52516B	Nondisplaced fracture of unspecified radial styloid process, initial encounter for open fracture type I or II
S52516C	Nondisplaced fracture of unspecified radial styloid process, initial encounter for open fracture type IIIA, IIIB, or IIIC
S52521A	Torus fracture of lower end of right radius, initial encounter for closed fracture
S52522A	Torus fracture of lower end of left radius, initial encounter for closed fracture
S52529A	Torus fracture of lower end of unspecified radius, initial encounter for closed fracture
S52531A	Colles' fracture of right radius, initial encounter for closed fracture
S52531B	Colles' fracture of right radius, initial encounter for open fracture type I or II
S52531C	Colles' fracture of right radius, initial encounter for open fracture type IIIA, IIIB, or IIIC
S52532A	Colles' fracture of left radius, initial encounter for closed fracture
S52532B	Colles' fracture of left radius, initial encounter for open fracture type I or II
S52532C	Colles' fracture of left radius, initial encounter for open fracture type IIIA, IIIB, or IIIC
S52539A	Colles' fracture of unspecified radius, initial encounter for closed fracture
S52539B	Colles' fracture of unspecified radius, initial encounter for open fracture type I or II
S52539C	Colles' fracture of unspecified radius, initial encounter for open fracture type IIIA, IIIB, or IIIC
S52541A	Smith's fracture of right radius, initial encounter for closed fracture
S52541B	Smith's fracture of right radius, initial encounter for open fracture type I or II
S52541C	Smith's fracture of right radius, initial encounter for open fracture type IIIA, IIIB, or IIIC
S52542A	Smith's fracture of left radius, initial encounter for closed fracture
S52542B	Smith's fracture of left radius, initial encounter for open fracture type I or II
S52542C	Smith's fracture of left radius, initial encounter for open fracture type IIIA, IIIB, or IIIC
S52549A	Smith's fracture of unspecified radius, initial encounter for closed fracture
S52549B	Smith's fracture of unspecified radius, initial encounter for open fracture type I or II
S52549C	Smith's fracture of unspecified radius, initial encounter for open fracture type IIIA, IIIB, or IIIC
S52551A	Other extraarticular fracture of lower end of right radius, initial encounter for closed fracture
S52551B	Other extraarticular fracture of lower end of right radius, initial encounter for open fracture type I or II
S52551C	Other extraarticular fracture of lower end of right radius, initial encounter for open fracture type IIIA, IIIB, or IIIC
S52552A	Other extraarticular fracture of lower end of left radius, initial encounter for closed fracture
S52552B	Other extraarticular fracture of lower end of left radius, initial encounter for open fracture type I or II
S52552C	Other extraarticular fracture of lower end of left radius, initial encounter for open fracture type IIIA, IIIB, or IIIC

S52559A	Other extraarticular fracture of lower end of unspecified radius, initial encounter for closed fracture
S52559B	Other extraarticular fracture of lower end of unspecified radius, initial encounter for open fracture type I or II
S52559C	Other extraarticular fracture of lower end of unspecified radius, initial encounter for open fracture type IIIA, IIIB, or IIIC
S52561A	Barton's fracture of right radius, initial encounter for closed fracture
S52561B	Barton's fracture of right radius, initial encounter for open fracture type I or II
S52561C	Barton's fracture of right radius, initial encounter for open fracture type IIIA, IIIB, or IIIC
S52562A	Barton's fracture of left radius, initial encounter for closed fracture
S52562B	Barton's fracture of left radius, initial encounter for open fracture type I or II
S52562C	Barton's fracture of left radius, initial encounter for open fracture type IIIA, IIIB, or IIIC
S52569A	Barton's fracture of unspecified radius, initial encounter for closed fracture
S52569B	Barton's fracture of unspecified radius, initial encounter for open fracture type I or II
S52569C	Barton's fracture of unspecified radius, initial encounter for open fracture type IIIA, IIIB, or IIIC
S52571A	Other intraarticular fracture of lower end of right radius, initial encounter for closed fracture
S52571B	Other intraarticular fracture of lower end of right radius, initial encounter for open fracture type I or II
S52571C	Other intraarticular fracture of lower end of right radius, initial encounter for open fracture type IIIA, IIIB, or IIIC
S52572A	Other intraarticular fracture of lower end of left radius, initial encounter for closed fracture
S52572B	Other intraarticular fracture of lower end of left radius, initial encounter for open fracture type I or II
S52572C	Other intraarticular fracture of lower end of left radius, initial encounter for open fracture type IIIA, IIIB, or IIIC
S52579A	Other intraarticular fracture of lower end of unspecified radius, initial encounter for closed fracture
S52579B	Other intraarticular fracture of lower end of unspecified radius, initial encounter for open fracture type I or II
S52579C	Other intraarticular fracture of lower end of unspecified radius, initial encounter for open fracture type IIIA, IIIB, or IIIC
S52591A	Other fractures of lower end of right radius, initial encounter for closed fracture
S52591B	Other fractures of lower end of right radius, initial encounter for open fracture type I or II
S52591C	Other fractures of lower end of right radius, initial encounter for open fracture type IIIA, IIIB, or IIIC
S52592A	Other fractures of lower end of left radius, initial encounter for closed fracture
S52592B	Other fractures of lower end of left radius, initial encounter for open fracture type I or II
S52592C	Other fractures of lower end of left radius, initial encounter for open fracture type IIIA, IIIB, or IIIC
S52599A	Other fractures of lower end of unspecified radius, initial encounter for closed fracture
S52599B	Other fractures of lower end of unspecified radius, initial encounter for open fracture type I or II
S52599C	Other fractures of lower end of unspecified radius, initial encounter for open fracture type IIIA, IIIB, or IIIC
S52601A	Unspecified fracture of lower end of right ulna, initial encounter for closed fracture
S52601B	Unspecified fracture of lower end of right ulna, initial encounter for open fracture type I or II
S52601C	Unspecified fracture of lower end of right ulna, initial encounter for open fracture type IIIA, IIIB, or IIIC
S52602A	Unspecified fracture of lower end of left ulna, initial encounter for closed fracture
S52602B	Unspecified fracture of lower end of left ulna, initial encounter for open fracture type I or II
S52602C	Unspecified fracture of lower end of left ulna, initial encounter for open fracture type IIIA, IIIB, or IIIC
S52609A	Unspecified fracture of lower end of unspecified ulna, initial encounter for closed fracture
S52609B	Unspecified fracture of lower end of unspecified ulna, initial encounter for open fracture type I or II

S52609C	Unspecified fracture of lower end of unspecified ulna, initial encounter for open fracture type IIIA, IIIB, or IIIC
S52611A	Displaced fracture of right ulna styloid process, initial encounter for closed fracture
S52611B	Displaced fracture of right ulna styloid process, initial encounter for open fracture type I or II
S52611C	Displaced fracture of right ulna styloid process, initial encounter for open fracture type IIIA, IIIB, or IIIC
S52612A	Displaced fracture of left ulna styloid process, initial encounter for closed fracture
S52612B	Displaced fracture of left ulna styloid process, initial encounter for open fracture type I or II
S52612C	Displaced fracture of left ulna styloid process, initial encounter for open fracture type IIIA, IIIB, or IIIC
S52613A	Displaced fracture of unspecified ulna styloid process, initial encounter for closed fracture
S52613B	Displaced fracture of unspecified ulna styloid process, initial encounter for open fracture type I or II
S52613C	Displaced fracture of unspecified ulna styloid process, initial encounter for open fracture type IIIA, IIIB, or IIIC
S52614A	Nondisplaced fracture of right ulna styloid process, initial encounter for closed fracture
S52614B	Nondisplaced fracture of right ulna styloid process, initial encounter for open fracture type I or II
S52614C	Nondisplaced fracture of right ulna styloid process, initial encounter for open fracture type IIIA, IIIB, or IIIC
S52615A	Nondisplaced fracture of left ulna styloid process, initial encounter for closed fracture
S52615B	Nondisplaced fracture of left ulna styloid process, initial encounter for open fracture type I or II
S52615C	Nondisplaced fracture of left ulna styloid process, initial encounter for open fracture type IIIA, IIIB, or IIIC
S52616A	Nondisplaced fracture of unspecified ulna styloid process, initial encounter for closed fracture
S52616B	Nondisplaced fracture of unspecified ulna styloid process, initial encounter for open fracture type I or II
S52616C	Nondisplaced fracture of unspecified ulna styloid process, initial encounter for open fracture type IIIA, IIIB, or IIIC
S52621A	Torus fracture of lower end of right ulna, initial encounter for closed fracture
S52622A	Torus fracture of lower end of left ulna, initial encounter for closed fracture
S52629A	Torus fracture of lower end of unspecified ulna, initial encounter for closed fracture
S52691A	Other fracture of lower end of right ulna, initial encounter for closed fracture
S52691B	Other fracture of lower end of right ulna, initial encounter for open fracture type I or II
S52691C	Other fracture of lower end of right ulna, initial encounter for open fracture type IIIA, IIIB, or IIIC
S52692A	Other fracture of lower end of left ulna, initial encounter for closed fracture
S52692B	Other fracture of lower end of left ulna, initial encounter for open fracture type I or II
S52692C	Other fracture of lower end of left ulna, initial encounter for open fracture type IIIA, IIIB, or IIIC
S52699A	Other fracture of lower end of unspecified ulna, initial encounter for closed fracture
S52699B	Other fracture of lower end of unspecified ulna, initial encounter for open fracture type I or II
S52699C	Other fracture of lower end of unspecified ulna, initial encounter for open fracture type IIIA, IIIB, or IIIC
S5290XA	Unspecified fracture of unspecified forearm, initial encounter for closed fracture
S5290XB	Unspecified fracture of unspecified forearm, initial encounter for open fracture type I or II
S5290XC	Unspecified fracture of unspecified forearm, initial encounter for open fracture type IIIA, IIIB, or IIIC
S5291XA	Unspecified fracture of right forearm, initial encounter for closed fracture
S5291XB	Unspecified fracture of right forearm, initial encounter for open fracture type I or II
S5291XC	Unspecified fracture of right forearm, initial encounter for open fracture type IIIA, IIIB, or IIIC
S5292XA	Unspecified fracture of left forearm, initial encounter for closed fracture
S5292XB	Unspecified fracture of left forearm, initial encounter for open fracture type I or II

S5292XC	Unspecified fracture of left forearm, initial encounter for open fracture type IIIA, IIIB, or IIIC
S59001A	Unspecified physeal fracture of lower end of ulna, right arm, initial encounter for closed fracture
S59002A	Unspecified physeal fracture of lower end of ulna, left arm, initial encounter for closed fracture
S59009A	Unspecified physeal fracture of lower end of ulna, unspecified arm, initial encounter for closed fracture
S59011A	Salter-Harris Type I physeal fracture of lower end of ulna, right arm, initial encounter for closed fracture
S59012A	Salter-Harris Type I physeal fracture of lower end of ulna, left arm, initial encounter for closed fracture
S59019A	Salter-Harris Type I physeal fracture of lower end of ulna, unspecified arm, initial encounter for closed fracture
S59021A	Salter-Harris Type II physeal fracture of lower end of ulna, right arm, initial encounter for closed fracture
S59022A	Salter-Harris Type II physeal fracture of lower end of ulna, left arm, initial encounter for closed fracture
S59029A	Salter-Harris Type II physeal fracture of lower end of ulna, unspecified arm, initial encounter for closed fracture
S59031A	Salter-Harris Type III physeal fracture of lower end of ulna, right arm, initial encounter for closed fracture
S59032A	Salter-Harris Type III physeal fracture of lower end of ulna, left arm, initial encounter for closed fracture
S59039A	Salter-Harris Type III physeal fracture of lower end of ulna, unspecified arm, initial encounter for closed fracture
S59041A	Salter-Harris Type IV physeal fracture of lower end of ulna, right arm, initial encounter for closed fracture
S59042A	Salter-Harris Type IV physeal fracture of lower end of ulna, left arm, initial encounter for closed fracture
S59049A	Salter-Harris Type IV physeal fracture of lower end of ulna, unspecified arm, initial encounter for closed fracture
S59091A	Other physeal fracture of lower end of ulna, right arm, initial encounter for closed fracture
S59092A	Other physeal fracture of lower end of ulna, left arm, initial encounter for closed fracture
S59099A	Other physeal fracture of lower end of ulna, unspecified arm, initial encounter for closed fracture
S59101A	Unspecified physeal fracture of upper end of radius, right arm, initial encounter for closed fracture
S59102A	Unspecified physeal fracture of upper end of radius, left arm, initial encounter for closed fracture
S59109A	Unspecified physeal fracture of upper end of radius, unspecified arm, initial encounter for closed fracture
S59111A	Salter-Harris Type I physeal fracture of upper end of radius, right arm, initial encounter for closed fracture
S59112A	Salter-Harris Type I physeal fracture of upper end of radius, left arm, initial encounter for closed fracture
S59119A	Salter-Harris Type I physeal fracture of upper end of radius, unspecified arm, initial encounter for closed fracture
S59121A	Salter-Harris Type II physeal fracture of upper end of radius, right arm, initial encounter for closed fracture
S59122A	Salter-Harris Type II physeal fracture of upper end of radius, left arm, initial encounter for closed fracture
S59129A	Salter-Harris Type II physeal fracture of upper end of radius, unspecified arm, initial encounter for closed fracture
S59131A	Salter-Harris Type III physeal fracture of upper end of radius, right arm, initial encounter for closed fracture
S59132A	Salter-Harris Type III physeal fracture of upper end of radius, left arm, initial encounter for closed fracture

S59139A	Salter-Harris Type III physeal fracture of upper end of radius, unspecified arm, initial encounter for closed fracture
S59141A	Salter-Harris Type IV physeal fracture of upper end of radius, right arm, initial encounter for closed fracture
S59142A	Salter-Harris Type IV physeal fracture of upper end of radius, left arm, initial encounter for closed fracture
S59149A	Salter-Harris Type IV physeal fracture of upper end of radius, unspecified arm, initial encounter for closed fracture
S59191A	Other physeal fracture of upper end of radius, right arm, initial encounter for closed fracture
S59192A	Other physeal fracture of upper end of radius, left arm, initial encounter for closed fracture
S59199A	Other physeal fracture of upper end of radius, unspecified arm, initial encounter for closed fracture
S59201A	Unspecified physeal fracture of lower end of radius, right arm, initial encounter for closed fracture
S59202A	Unspecified physeal fracture of lower end of radius, left arm, initial encounter for closed fracture
S59209A	Unspecified physeal fracture of lower end of radius, unspecified arm, initial encounter for closed fracture
S59211A	Salter-Harris Type I physeal fracture of lower end of radius, right arm, initial encounter for closed fracture
S59212A	Salter-Harris Type I physeal fracture of lower end of radius, left arm, initial encounter for closed fracture
S59219A	Salter-Harris Type I physeal fracture of lower end of radius, unspecified arm, initial encounter for closed fracture
S59221A	Salter-Harris Type II physeal fracture of lower end of radius, right arm, initial encounter for closed fracture
S59222A	Salter-Harris Type II physeal fracture of lower end of radius, left arm, initial encounter for closed fracture
S59229A	Salter-Harris Type II physeal fracture of lower end of radius, unspecified arm, initial encounter for closed fracture
S59231A	Salter-Harris Type III physeal fracture of lower end of radius, right arm, initial encounter for closed fracture
S59232A	Salter-Harris Type III physeal fracture of lower end of radius, left arm, initial encounter for closed fracture
S59239A	Salter-Harris Type III physeal fracture of lower end of radius, unspecified arm, initial encounter for closed fracture
S59241A	Salter-Harris Type IV physeal fracture of lower end of radius, right arm, initial encounter for closed fracture
S59242A	Salter-Harris Type IV physeal fracture of lower end of radius, left arm, initial encounter for closed fracture
S59249A	Salter-Harris Type IV physeal fracture of lower end of radius, unspecified arm, initial encounter for closed fracture
S59291A	Other physeal fracture of lower end of radius, right arm, initial encounter for closed fracture
S59292A	Other physeal fracture of lower end of radius, left arm, initial encounter for closed fracture
S59299A	Other physeal fracture of lower end of radius, unspecified arm, initial encounter for closed fracture
S62001A	Unspecified fracture of navicular [scaphoid] bone of right wrist, initial encounter for closed fracture
S62002A	Unspecified fracture of navicular [scaphoid] bone of left wrist, initial encounter for closed fracture
S62009A	Unspecified fracture of navicular [scaphoid] bone of unspecified wrist, initial encounter for closed fracture
S62011A	Displaced fracture of distal pole of navicular [scaphoid] bone of right wrist, initial encounter for closed fracture
S62012A	Displaced fracture of distal pole of navicular [scaphoid] bone of left wrist, initial encounter for closed fracture

S62013A	Displaced fracture of distal pole of navicular [scaphoid] bone of unspecified wrist, initial encounter for closed fracture
S62014A	Nondisplaced fracture of distal pole of navicular [scaphoid] bone of right wrist, initial encounter for closed fracture
S62015A	Nondisplaced fracture of distal pole of navicular [scaphoid] bone of left wrist, initial encounter for closed fracture
S62016A	Nondisplaced fracture of distal pole of navicular [scaphoid] bone of unspecified wrist, initial encounter for closed fracture
S62021A	Displaced fracture of middle third of navicular [scaphoid] bone of right wrist, initial encounter for closed fracture
S62022A	Displaced fracture of middle third of navicular [scaphoid] bone of left wrist, initial encounter for closed fracture
S62023A	Displaced fracture of middle third of navicular [scaphoid] bone of unspecified wrist, initial encounter for closed fracture
S62024A	Nondisplaced fracture of middle third of navicular [scaphoid] bone of right wrist, initial encounter for closed fracture
S62025A	Nondisplaced fracture of middle third of navicular [scaphoid] bone of left wrist, initial encounter for closed fracture
S62026A	Nondisplaced fracture of middle third of navicular [scaphoid] bone of unspecified wrist, initial encounter for closed fracture
S62031A	Displaced fracture of proximal third of navicular [scaphoid] bone of right wrist, initial encounter for closed fracture
S62032A	Displaced fracture of proximal third of navicular [scaphoid] bone of left wrist, initial encounter for closed fracture
S62033A	Displaced fracture of proximal third of navicular [scaphoid] bone of unspecified wrist, initial encounter for closed fracture
S62034A	Nondisplaced fracture of proximal third of navicular [scaphoid] bone of right wrist, initial encounter for closed fracture
S62035A	Nondisplaced fracture of proximal third of navicular [scaphoid] bone of left wrist, initial encounter for closed fracture
S62036A	Nondisplaced fracture of proximal third of navicular [scaphoid] bone of unspecified wrist, initial encounter for closed fracture
S62101A	Fracture of unspecified carpal bone, right wrist, initial encounter for closed fracture
S62101B	Fracture of unspecified carpal bone, right wrist, initial encounter for open fracture
S62102A	Fracture of unspecified carpal bone, left wrist, initial encounter for closed fracture
S62102B	Fracture of unspecified carpal bone, left wrist, initial encounter for open fracture
S62109A	Fracture of unspecified carpal bone, unspecified wrist, initial encounter for closed fracture
S62109B	Fracture of unspecified carpal bone, unspecified wrist, initial encounter for open fracture
S62111A	Displaced fracture of triquetrum [cuneiform] bone, right wrist, initial encounter for closed fracture
S62111B	Displaced fracture of triquetrum [cuneiform] bone, right wrist, initial encounter for open fracture
S62112A	Displaced fracture of triquetrum [cuneiform] bone, left wrist, initial encounter for closed fracture
S62112B	Displaced fracture of triquetrum [cuneiform] bone, left wrist, initial encounter for open fracture
S62113A	Displaced fracture of triquetrum [cuneiform] bone, unspecified wrist, initial encounter for closed fracture
S62113B	Displaced fracture of triquetrum [cuneiform] bone, unspecified wrist, initial encounter for open fracture
S62114A	Nondisplaced fracture of triquetrum [cuneiform] bone, right wrist, initial encounter for closed fracture
S62114B	Nondisplaced fracture of triquetrum [cuneiform] bone, right wrist, initial encounter for open fracture
S62115A	Nondisplaced fracture of triquetrum [cuneiform] bone, left wrist, initial encounter for closed fracture

S62115B	Nondisplaced fracture of triquetrum [cuneiform] bone, left wrist, initial encounter for open fracture
S62116A	Nondisplaced fracture of triquetrum [cuneiform] bone, unspecified wrist, initial encounter for closed fracture
S62116B	Nondisplaced fracture of triquetrum [cuneiform] bone, unspecified wrist, initial encounter for open fracture
S62121A	Displaced fracture of lunate [semilunar], right wrist, initial encounter for closed fracture
S62121B	Displaced fracture of lunate [semilunar], right wrist, initial encounter for open fracture
S62122A	Displaced fracture of lunate [semilunar], left wrist, initial encounter for closed fracture
S62122B	Displaced fracture of lunate [semilunar], left wrist, initial encounter for open fracture
S62123A	Displaced fracture of lunate [semilunar], unspecified wrist, initial encounter for closed fracture
S62123B	Displaced fracture of lunate [semilunar], unspecified wrist, initial encounter for open fracture
S62124A	Nondisplaced fracture of lunate [semilunar], right wrist, initial encounter for closed fracture
S62124B	Nondisplaced fracture of lunate [semilunar], right wrist, initial encounter for open fracture
S62125A	Nondisplaced fracture of lunate [semilunar], left wrist, initial encounter for closed fracture
S62125B	Nondisplaced fracture of lunate [semilunar], left wrist, initial encounter for open fracture
S62126A	Nondisplaced fracture of lunate [semilunar], unspecified wrist, initial encounter for closed fracture
S62126B	Nondisplaced fracture of lunate [semilunar], unspecified wrist, initial encounter for open fracture
S62131A	Displaced fracture of capitate [os magnum] bone, right wrist, initial encounter for closed fracture
S62131B	Displaced fracture of capitate [os magnum] bone, right wrist, initial encounter for open fracture
S62132A	Displaced fracture of capitate [os magnum] bone, left wrist, initial encounter for closed fracture
S62132B	Displaced fracture of capitate [os magnum] bone, left wrist, initial encounter for open fracture
S62133A	Displaced fracture of capitate [os magnum] bone, unspecified wrist, initial encounter for closed fracture
S62133B	Displaced fracture of capitate [os magnum] bone, unspecified wrist, initial encounter for open fracture
S62134A	Nondisplaced fracture of capitate [os magnum] bone, right wrist, initial encounter for closed fracture
S62134B	Nondisplaced fracture of capitate [os magnum] bone, right wrist, initial encounter for open fracture
S62135A	Nondisplaced fracture of capitate [os magnum] bone, left wrist, initial encounter for closed fracture
S62135B	Nondisplaced fracture of capitate [os magnum] bone, left wrist, initial encounter for open fracture
S62136A	Nondisplaced fracture of capitate [os magnum] bone, unspecified wrist, initial encounter for closed fracture
S62136B	Nondisplaced fracture of capitate [os magnum] bone, unspecified wrist, initial encounter for open fracture
S62141A	Displaced fracture of body of hamate [unciform] bone, right wrist, initial encounter for closed fracture
S62141B	Displaced fracture of body of hamate [unciform] bone, right wrist, initial encounter for open fracture
S62142A	Displaced fracture of body of hamate [unciform] bone, left wrist, initial encounter for closed fracture
S62142B	Displaced fracture of body of hamate [unciform] bone, left wrist, initial encounter for open fracture
S62143A	Displaced fracture of body of hamate [unciform] bone, unspecified wrist, initial encounter for closed fracture
S62143B	Displaced fracture of body of hamate [unciform] bone, unspecified wrist, initial encounter for open fracture
S62144A	Nondisplaced fracture of body of hamate [unciform] bone, right wrist, initial encounter for closed fracture
S62144B	Nondisplaced fracture of body of hamate [unciform] bone, right wrist, initial encounter for open fracture
S62145A	Nondisplaced fracture of body of hamate [unciform] bone, left wrist, initial encounter for closed fracture

S62145B	Nondisplaced fracture of body of hamate [unciform] bone, left wrist, initial encounter for open fracture
S62146A	Nondisplaced fracture of body of hamate [unciform] bone, unspecified wrist, initial encounter for closed fracture
S62146B	Nondisplaced fracture of body of hamate [unciform] bone, unspecified wrist, initial encounter for open fracture
S62151A	Displaced fracture of hook process of hamate [unciform] bone, right wrist, initial encounter for closed fracture
S62151B	Displaced fracture of hook process of hamate [unciform] bone, right wrist, initial encounter for open fracture
S62152A	Displaced fracture of hook process of hamate [unciform] bone, left wrist, initial encounter for closed fracture
S62152B	Displaced fracture of hook process of hamate [unciform] bone, left wrist, initial encounter for open fracture
S62153A	Displaced fracture of hook process of hamate [unciform] bone, unspecified wrist, initial encounter for closed fracture
S62153B	Displaced fracture of hook process of hamate [unciform] bone, unspecified wrist, initial encounter for open fracture
S62154A	Nondisplaced fracture of hook process of hamate [unciform] bone, right wrist, initial encounter for closed fracture
S62154B	Nondisplaced fracture of hook process of hamate [unciform] bone, right wrist, initial encounter for open fracture
S62155A	Nondisplaced fracture of hook process of hamate [unciform] bone, left wrist, initial encounter for closed fracture
S62155B	Nondisplaced fracture of hook process of hamate [unciform] bone, left wrist, initial encounter for open fracture
S62156A	Nondisplaced fracture of hook process of hamate [unciform] bone, unspecified wrist, initial encounter for closed fracture
S62156B	Nondisplaced fracture of hook process of hamate [unciform] bone, unspecified wrist, initial encounter for open fracture
S62161A	Displaced fracture of pisiform, right wrist, initial encounter for closed fracture
S62161B	Displaced fracture of pisiform, right wrist, initial encounter for open fracture
S62162A	Displaced fracture of pisiform, left wrist, initial encounter for closed fracture
S62162B	Displaced fracture of pisiform, left wrist, initial encounter for open fracture
S62163A	Displaced fracture of pisiform, unspecified wrist, initial encounter for closed fracture
S62163B	Displaced fracture of pisiform, unspecified wrist, initial encounter for open fracture
S62164A	Nondisplaced fracture of pisiform, right wrist, initial encounter for closed fracture
S62164B	Nondisplaced fracture of pisiform, right wrist, initial encounter for open fracture
S62165A	Nondisplaced fracture of pisiform, left wrist, initial encounter for closed fracture
S62165B	Nondisplaced fracture of pisiform, left wrist, initial encounter for open fracture
S62166A	Nondisplaced fracture of pisiform, unspecified wrist, initial encounter for closed fracture
S62166B	Nondisplaced fracture of pisiform, unspecified wrist, initial encounter for open fracture
S62171A	Displaced fracture of trapezium [larger multangular], right wrist, initial encounter for closed fracture
S62171B	Displaced fracture of trapezium [larger multangular], right wrist, initial encounter for open fracture
S62172A	Displaced fracture of trapezium [larger multangular], left wrist, initial encounter for closed fracture
S62172B	Displaced fracture of trapezium [larger multangular], left wrist, initial encounter for open fracture
S62173A	Displaced fracture of trapezium [larger multangular], unspecified wrist, initial encounter for closed fracture

S62173B	Displaced fracture of trapezium [larger multangular], unspecified wrist, initial encounter for open fracture
S62174A	Nondisplaced fracture of trapezium [larger multangular], right wrist, initial encounter for closed fracture
S62174B	Nondisplaced fracture of trapezium [larger multangular], right wrist, initial encounter for open fracture
S62175A	Nondisplaced fracture of trapezium [larger multangular], left wrist, initial encounter for closed fracture
S62175B	Nondisplaced fracture of trapezium [larger multangular], left wrist, initial encounter for open fracture
S62176A	Nondisplaced fracture of trapezium [larger multangular], unspecified wrist, initial encounter for closed fracture
S62176B	Nondisplaced fracture of trapezium [larger multangular], unspecified wrist, initial encounter for open fracture
S62181A	Displaced fracture of trapezoid [smaller multangular], right wrist, initial encounter for closed fracture
S62181B	Displaced fracture of trapezoid [smaller multangular], right wrist, initial encounter for open fracture
S62182A	Displaced fracture of trapezoid [smaller multangular], left wrist, initial encounter for closed fracture
S62182B	Displaced fracture of trapezoid [smaller multangular], left wrist, initial encounter for open fracture
S62183A	Displaced fracture of trapezoid [smaller multangular], unspecified wrist, initial encounter for closed fracture
S62183B	Displaced fracture of trapezoid [smaller multangular], unspecified wrist, initial encounter for open fracture
S62184A	Nondisplaced fracture of trapezoid [smaller multangular], right wrist, initial encounter for closed fracture
S62184B	Nondisplaced fracture of trapezoid [smaller multangular], right wrist, initial encounter for open fracture
S62185A	Nondisplaced fracture of trapezoid [smaller multangular], left wrist, initial encounter for closed fracture
S62185B	Nondisplaced fracture of trapezoid [smaller multangular], left wrist, initial encounter for open fracture
S62186A	Nondisplaced fracture of trapezoid [smaller multangular], unspecified wrist, initial encounter for closed fracture
S62186B	Nondisplaced fracture of trapezoid [smaller multangular], unspecified wrist, initial encounter for open fracture
S6290XA	Unspecified fracture of unspecified wrist and hand, initial encounter for closed fracture
S6291XA	Unspecified fracture of right wrist and hand, initial encounter for closed fracture
S6292XA	Unspecified fracture of left wrist and hand, initial encounter for closed fracture
S72001A	Fracture of unspecified part of neck of right femur, initial encounter for closed fracture
S72001B	Fracture of unspecified part of neck of right femur, initial encounter for open fracture type I or II
S72001C	Fracture of unspecified part of neck of right femur, initial encounter for open fracture type IIIA, IIIB, or IIIC
S72002A	Fracture of unspecified part of neck of left femur, initial encounter for closed fracture
S72002B	Fracture of unspecified part of neck of left femur, initial encounter for open fracture type I or II
S72002C	Fracture of unspecified part of neck of left femur, initial encounter for open fracture type IIIA, IIIB, or IIIC
S72009A	Fracture of unspecified part of neck of unspecified femur, initial encounter for closed fracture
S72009B	Fracture of unspecified part of neck of unspecified femur, initial encounter for open fracture type I or II
S72009C	Fracture of unspecified part of neck of unspecified femur, initial encounter for open fracture type IIIA, IIIB, or IIIC
S72011A	Unspecified intracapsular fracture of right femur, initial encounter for closed fracture
S72011B	Unspecified intracapsular fracture of right femur, initial encounter for open fracture type I or II
S72011C	Unspecified intracapsular fracture of right femur, initial encounter for open fracture type IIIA, IIIB, or

	IIIC
S72012A	Unspecified intracapsular fracture of left femur, initial encounter for closed fracture
S72012B	Unspecified intracapsular fracture of left femur, initial encounter for open fracture type I or II
S72012C	Unspecified intracapsular fracture of left femur, initial encounter for open fracture type IIIA, IIIB, or IIIC
S72019A	Unspecified intracapsular fracture of unspecified femur, initial encounter for closed fracture
S72019B	Unspecified intracapsular fracture of unspecified femur, initial encounter for open fracture type I or II
S72019C	Unspecified intracapsular fracture of unspecified femur, initial encounter for open fracture type IIIA, IIIB, or IIIC
S72021A	Displaced fracture of epiphysis (separation) (upper) of right femur, initial encounter for closed fracture
S72021B	Displaced fracture of epiphysis (separation) (upper) of right femur, initial encounter for open fracture type I or II
S72021C	Displaced fracture of epiphysis (separation) (upper) of right femur, initial encounter for open fracture type IIIA, IIIB, or IIIC
S72022A	Displaced fracture of epiphysis (separation) (upper) of left femur, initial encounter for closed fracture
S72022B	Displaced fracture of epiphysis (separation) (upper) of left femur, initial encounter for open fracture type I or II
S72022C	Displaced fracture of epiphysis (separation) (upper) of left femur, initial encounter for open fracture type IIIA, IIIB, or IIIC
S72023A	Displaced fracture of epiphysis (separation) (upper) of unspecified femur, initial encounter for closed fracture
S72023B	Displaced fracture of epiphysis (separation) (upper) of unspecified femur, initial encounter for open fracture type I or II
S72023C	Displaced fracture of epiphysis (separation) (upper) of unspecified femur, initial encounter for open fracture type IIIA, IIIB, or IIIC
S72024A	Nondisplaced fracture of epiphysis (separation) (upper) of right femur, initial encounter for closed fracture
S72024B	Nondisplaced fracture of epiphysis (separation) (upper) of right femur, initial encounter for open fracture type I or II
S72024C	Nondisplaced fracture of epiphysis (separation) (upper) of right femur, initial encounter for open fracture type IIIA, IIIB, or IIIC
S72025A	Nondisplaced fracture of epiphysis (separation) (upper) of left femur, initial encounter for closed fracture
S72025B	Nondisplaced fracture of epiphysis (separation) (upper) of left femur, initial encounter for open fracture type I or II
S72025C	Nondisplaced fracture of epiphysis (separation) (upper) of left femur, initial encounter for open fracture type IIIA, IIIB, or IIIC
S72026A	Nondisplaced fracture of epiphysis (separation) (upper) of unspecified femur, initial encounter for closed fracture
S72026B	Nondisplaced fracture of epiphysis (separation) (upper) of unspecified femur, initial encounter for open fracture type I or II
S72026C	Nondisplaced fracture of epiphysis (separation) (upper) of unspecified femur, initial encounter for open fracture type IIIA, IIIB, or IIIC
S72031A	Displaced midcervical fracture of right femur, initial encounter for closed fracture
S72031B	Displaced midcervical fracture of right femur, initial encounter for open fracture type I or II
S72031C	Displaced midcervical fracture of right femur, initial encounter for open fracture type IIIA, IIIB, or IIIC
S72032A	Displaced midcervical fracture of left femur, initial encounter for closed fracture
S72032B	Displaced midcervical fracture of left femur, initial encounter for open fracture type I or II
S72032C	Displaced midcervical fracture of left femur, initial encounter for open fracture type IIIA, IIIB, or IIIC
S72033A	Displaced midcervical fracture of unspecified femur, initial encounter for closed fracture

S72033B	Displaced midcervical fracture of unspecified femur, initial encounter for open fracture type I or II
S72033C	Displaced midcervical fracture of unspecified femur, initial encounter for open fracture type IIIA, IIIB, or IIIC
S72034A	Nondisplaced midcervical fracture of right femur, initial encounter for closed fracture
S72034B	Nondisplaced midcervical fracture of right femur, initial encounter for open fracture type I or II
S72034C	Nondisplaced midcervical fracture of right femur, initial encounter for open fracture type IIIA, IIIB, or IIIC
S72035A	Nondisplaced midcervical fracture of left femur, initial encounter for closed fracture
S72035B	Nondisplaced midcervical fracture of left femur, initial encounter for open fracture type I or II
S72035C	Nondisplaced midcervical fracture of left femur, initial encounter for open fracture type IIIA, IIIB, or IIIC
S72036A	Nondisplaced midcervical fracture of unspecified femur, initial encounter for closed fracture
S72036B	Nondisplaced midcervical fracture of unspecified femur, initial encounter for open fracture type I or II
S72036C	Nondisplaced midcervical fracture of unspecified femur, initial encounter for open fracture type IIIA, IIIB, or IIIC
S72041A	Displaced fracture of base of neck of right femur, initial encounter for closed fracture
S72041B	Displaced fracture of base of neck of right femur, initial encounter for open fracture type I or II
S72041C	Displaced fracture of base of neck of right femur, initial encounter for open fracture type IIIA, IIIB, or IIIC
S72042A	Displaced fracture of base of neck of left femur, initial encounter for closed fracture
S72042B	Displaced fracture of base of neck of left femur, initial encounter for open fracture type I or II
S72042C	Displaced fracture of base of neck of left femur, initial encounter for open fracture type IIIA, IIIB, or IIIC
S72043A	Displaced fracture of base of neck of unspecified femur, initial encounter for closed fracture
S72043B	Displaced fracture of base of neck of unspecified femur, initial encounter for open fracture type I or II
S72043C	Displaced fracture of base of neck of unspecified femur, initial encounter for open fracture type IIIA, IIIB, or IIIC
S72044A	Nondisplaced fracture of base of neck of right femur, initial encounter for closed fracture
S72044B	Nondisplaced fracture of base of neck of right femur, initial encounter for open fracture type I or II
S72044C	Nondisplaced fracture of base of neck of right femur, initial encounter for open fracture type IIIA, IIIB, or IIIC
S72045A	Nondisplaced fracture of base of neck of left femur, initial encounter for closed fracture
S72045B	Nondisplaced fracture of base of neck of left femur, initial encounter for open fracture type I or II
S72045C	Nondisplaced fracture of base of neck of left femur, initial encounter for open fracture type IIIA, IIIB, or IIIC
S72046A	Nondisplaced fracture of base of neck of unspecified femur, initial encounter for closed fracture
S72046B	Nondisplaced fracture of base of neck of unspecified femur, initial encounter for open fracture type I or II
S72046C	Nondisplaced fracture of base of neck of unspecified femur, initial encounter for open fracture type IIIA, IIIB, or IIIC
S72051A	Unspecified fracture of head of right femur, initial encounter for closed fracture
S72051B	Unspecified fracture of head of right femur, initial encounter for open fracture type I or II
S72051C	Unspecified fracture of head of right femur, initial encounter for open fracture type IIIA, IIIB, or IIIC
S72052A	Unspecified fracture of head of left femur, initial encounter for closed fracture
S72052B	Unspecified fracture of head of left femur, initial encounter for open fracture type I or II
S72052C	Unspecified fracture of head of left femur, initial encounter for open fracture type IIIA, IIIB, or IIIC
S72059A	Unspecified fracture of head of unspecified femur, initial encounter for closed fracture
S72059B	Unspecified fracture of head of unspecified femur, initial encounter for open fracture type I or II

S72059C	Unspecified fracture of head of unspecified femur, initial encounter for open fracture type IIIA, IIIB, or IIIC
S72061A	Displaced articular fracture of head of right femur, initial encounter for closed fracture
S72061B	Displaced articular fracture of head of right femur, initial encounter for open fracture type I or II
S72061C	Displaced articular fracture of head of right femur, initial encounter for open fracture type IIIA, IIIB, or IIIC
S72062A	Displaced articular fracture of head of left femur, initial encounter for closed fracture
S72062B	Displaced articular fracture of head of left femur, initial encounter for open fracture type I or II
S72062C	Displaced articular fracture of head of left femur, initial encounter for open fracture type IIIA, IIIB, or IIIC
S72063A	Displaced articular fracture of head of unspecified femur, initial encounter for closed fracture
S72063B	Displaced articular fracture of head of unspecified femur, initial encounter for open fracture type I or II
S72063C	Displaced articular fracture of head of unspecified femur, initial encounter for open fracture type IIIA, IIIB, or IIIC
S72064A	Nondisplaced articular fracture of head of right femur, initial encounter for closed fracture
S72064B	Nondisplaced articular fracture of head of right femur, initial encounter for open fracture type I or II
S72064C	Nondisplaced articular fracture of head of right femur, initial encounter for open fracture type IIIA, IIIB, or IIIC
S72065A	Nondisplaced articular fracture of head of left femur, initial encounter for closed fracture
S72065B	Nondisplaced articular fracture of head of left femur, initial encounter for open fracture type I or II
S72065C	Nondisplaced articular fracture of head of left femur, initial encounter for open fracture type IIIA, IIIB, or IIIC
S72066A	Nondisplaced articular fracture of head of unspecified femur, initial encounter for closed fracture
S72066B	Nondisplaced articular fracture of head of unspecified femur, initial encounter for open fracture type I or II
S72066C	Nondisplaced articular fracture of head of unspecified femur, initial encounter for open fracture type IIIA, IIIB, or IIIC
S72091A	Other fracture of head and neck of right femur, initial encounter for closed fracture
S72091B	Other fracture of head and neck of right femur, initial encounter for open fracture type I or II
S72091C	Other fracture of head and neck of right femur, initial encounter for open fracture type IIIA, IIIB, or IIIC
S72092A	Other fracture of head and neck of left femur, initial encounter for closed fracture
S72092B	Other fracture of head and neck of left femur, initial encounter for open fracture type I or II
S72092C	Other fracture of head and neck of left femur, initial encounter for open fracture type IIIA, IIIB, or IIIC
S72099A	Other fracture of head and neck of unspecified femur, initial encounter for closed fracture
S72099B	Other fracture of head and neck of unspecified femur, initial encounter for open fracture type I or II
S72099C	Other fracture of head and neck of unspecified femur, initial encounter for open fracture type IIIA, IIIB, or IIIC
S72101A	Unspecified trochanteric fracture of right femur, initial encounter for closed fracture
S72101B	Unspecified trochanteric fracture of right femur, initial encounter for open fracture type I or II
S72101C	Unspecified trochanteric fracture of right femur, initial encounter for open fracture type IIIA, IIIB, or IIIC
S72102A	Unspecified trochanteric fracture of left femur, initial encounter for closed fracture
S72102B	Unspecified trochanteric fracture of left femur, initial encounter for open fracture type I or II
S72102C	Unspecified trochanteric fracture of left femur, initial encounter for open fracture type IIIA, IIIB, or IIIC
S72109A	Unspecified trochanteric fracture of unspecified femur, initial encounter for closed fracture
S72109B	Unspecified trochanteric fracture of unspecified femur, initial encounter for open fracture type I or II

S72109C	Unspecified trochanteric fracture of unspecified femur, initial encounter for open fracture type IIIA, IIIB, or IIIC
S72111A	Displaced fracture of greater trochanter of right femur, initial encounter for closed fracture
S72111B	Displaced fracture of greater trochanter of right femur, initial encounter for open fracture type I or II
S72111C	Displaced fracture of greater trochanter of right femur, initial encounter for open fracture type IIIA, IIIB, or IIIC
S72112A	Displaced fracture of greater trochanter of left femur, initial encounter for closed fracture
S72112B	Displaced fracture of greater trochanter of left femur, initial encounter for open fracture type I or II
S72112C	Displaced fracture of greater trochanter of left femur, initial encounter for open fracture type IIIA, IIIB, or IIIC
S72113A	Displaced fracture of greater trochanter of unspecified femur, initial encounter for closed fracture
S72113B	Displaced fracture of greater trochanter of unspecified femur, initial encounter for open fracture type I or II
S72113C	Displaced fracture of greater trochanter of unspecified femur, initial encounter for open fracture type IIIA, IIIB, or IIIC
S72114A	Nondisplaced fracture of greater trochanter of right femur, initial encounter for closed fracture
S72114B	Nondisplaced fracture of greater trochanter of right femur, initial encounter for open fracture type I or II
S72114C	Nondisplaced fracture of greater trochanter of right femur, initial encounter for open fracture type IIIA, IIIB, or IIIC
S72115A	Nondisplaced fracture of greater trochanter of left femur, initial encounter for closed fracture
S72115B	Nondisplaced fracture of greater trochanter of left femur, initial encounter for open fracture type I or II
S72115C	Nondisplaced fracture of greater trochanter of left femur, initial encounter for open fracture type IIIA, IIIB, or IIIC
S72116A	Nondisplaced fracture of greater trochanter of unspecified femur, initial encounter for closed fracture
S72116B	Nondisplaced fracture of greater trochanter of unspecified femur, initial encounter for open fracture type I or II
S72116C	Nondisplaced fracture of greater trochanter of unspecified femur, initial encounter for open fracture type IIIA, IIIB, or IIIC
S72121A	Displaced fracture of lesser trochanter of right femur, initial encounter for closed fracture
S72121B	Displaced fracture of lesser trochanter of right femur, initial encounter for open fracture type I or II
S72121C	Displaced fracture of lesser trochanter of right femur, initial encounter for open fracture type IIIA, IIIB, or IIIC
S72122A	Displaced fracture of lesser trochanter of left femur, initial encounter for closed fracture
S72122B	Displaced fracture of lesser trochanter of left femur, initial encounter for open fracture type I or II
S72122C	Displaced fracture of lesser trochanter of left femur, initial encounter for open fracture type IIIA, IIIB, or IIIC
S72123A	Displaced fracture of lesser trochanter of unspecified femur, initial encounter for closed fracture
S72123B	Displaced fracture of lesser trochanter of unspecified femur, initial encounter for open fracture type I or II
S72123C	Displaced fracture of lesser trochanter of unspecified femur, initial encounter for open fracture type IIIA, IIIB, or IIIC
S72124A	Nondisplaced fracture of lesser trochanter of right femur, initial encounter for closed fracture
S72124B	Nondisplaced fracture of lesser trochanter of right femur, initial encounter for open fracture type I or II
S72124C	Nondisplaced fracture of lesser trochanter of right femur, initial encounter for open fracture type IIIA, IIIB, or IIIC
S72125A	Nondisplaced fracture of lesser trochanter of left femur, initial encounter for closed fracture

S72125B	Nondisplaced fracture of lesser trochanter of left femur, initial encounter for open fracture type I or II
S72125C	Nondisplaced fracture of lesser trochanter of left femur, initial encounter for open fracture type IIIA, IIIB, or IIIC
S72126A	Nondisplaced fracture of lesser trochanter of unspecified femur, initial encounter for closed fracture
S72126B	Nondisplaced fracture of lesser trochanter of unspecified femur, initial encounter for open fracture type I or II
S72126C	Nondisplaced fracture of lesser trochanter of unspecified femur, initial encounter for open fracture type IIIA, IIIB, or IIIC
S72131A	Displaced apophyseal fracture of right femur, initial encounter for closed fracture
S72131B	Displaced apophyseal fracture of right femur, initial encounter for open fracture type I or II
S72131C	Displaced apophyseal fracture of right femur, initial encounter for open fracture type IIIA, IIIB, or IIIC
S72132A	Displaced apophyseal fracture of left femur, initial encounter for closed fracture
S72132B	Displaced apophyseal fracture of left femur, initial encounter for open fracture type I or II
S72132C	Displaced apophyseal fracture of left femur, initial encounter for open fracture type IIIA, IIIB, or IIIC
S72133A	Displaced apophyseal fracture of unspecified femur, initial encounter for closed fracture
S72133B	Displaced apophyseal fracture of unspecified femur, initial encounter for open fracture type I or II
S72133C	Displaced apophyseal fracture of unspecified femur, initial encounter for open fracture type IIIA, IIIB, or IIIC
S72134A	Nondisplaced apophyseal fracture of right femur, initial encounter for closed fracture
S72134B	Nondisplaced apophyseal fracture of right femur, initial encounter for open fracture type I or II
S72134C	Nondisplaced apophyseal fracture of right femur, initial encounter for open fracture type IIIA, IIIB, or IIIC
S72135A	Nondisplaced apophyseal fracture of left femur, initial encounter for closed fracture
S72135B	Nondisplaced apophyseal fracture of left femur, initial encounter for open fracture type I or II
S72135C	Nondisplaced apophyseal fracture of left femur, initial encounter for open fracture type IIIA, IIIB, or IIIC
S72136A	Nondisplaced apophyseal fracture of unspecified femur, initial encounter for closed fracture
S72136B	Nondisplaced apophyseal fracture of unspecified femur, initial encounter for open fracture type I or II
S72136C	Nondisplaced apophyseal fracture of unspecified femur, initial encounter for open fracture type IIIA, IIIB, or IIIC
S72141A	Displaced intertrochanteric fracture of right femur, initial encounter for closed fracture
S72141B	Displaced intertrochanteric fracture of right femur, initial encounter for open fracture type I or II
S72141C	Displaced intertrochanteric fracture of right femur, initial encounter for open fracture type IIIA, IIIB, or IIIC
S72142A	Displaced intertrochanteric fracture of left femur, initial encounter for closed fracture
S72142B	Displaced intertrochanteric fracture of left femur, initial encounter for open fracture type I or II
S72142C	Displaced intertrochanteric fracture of left femur, initial encounter for open fracture type IIIA, IIIB, or IIIC
S72143A	Displaced intertrochanteric fracture of unspecified femur, initial encounter for closed fracture
S72143B	Displaced intertrochanteric fracture of unspecified femur, initial encounter for open fracture type I or II
S72143C	Displaced intertrochanteric fracture of unspecified femur, initial encounter for open fracture type IIIA, IIIB, or IIIC
S72144A	Nondisplaced intertrochanteric fracture of right femur, initial encounter for closed fracture
S72144B	Nondisplaced intertrochanteric fracture of right femur, initial encounter for open fracture type I or II
S72144C	Nondisplaced intertrochanteric fracture of right femur, initial encounter for open fracture type IIIA, IIIB, or IIIC
S72145A	Nondisplaced intertrochanteric fracture of left femur, initial encounter for closed fracture

S72145B	Nondisplaced intertrochanteric fracture of left femur, initial encounter for open fracture type I or II
S72145C	Nondisplaced intertrochanteric fracture of left femur, initial encounter for open fracture type IIIA, IIIB, or IIIC
S72146A	Nondisplaced intertrochanteric fracture of unspecified femur, initial encounter for closed fracture
S72146B	Nondisplaced intertrochanteric fracture of unspecified femur, initial encounter for open fracture type I or II
S72146C	Nondisplaced intertrochanteric fracture of unspecified femur, initial encounter for open fracture type IIIA, IIIB, or IIIC
S7221XA	Displaced subtrochanteric fracture of right femur, initial encounter for closed fracture
S7221XB	Displaced subtrochanteric fracture of right femur, initial encounter for open fracture type I or II
S7221XC	Displaced subtrochanteric fracture of right femur, initial encounter for open fracture type IIIA, IIIB, or IIIC
S7222XA	Displaced subtrochanteric fracture of left femur, initial encounter for closed fracture
S7222XB	Displaced subtrochanteric fracture of left femur, initial encounter for open fracture type I or II
S7222XC	Displaced subtrochanteric fracture of left femur, initial encounter for open fracture type IIIA, IIIB, or IIIC
S7223XA	Displaced subtrochanteric fracture of unspecified femur, initial encounter for closed fracture
S7223XB	Displaced subtrochanteric fracture of unspecified femur, initial encounter for open fracture type I or II
S7223XC	Displaced subtrochanteric fracture of unspecified femur, initial encounter for open fracture type IIIA, IIIB, or IIIC
S7224XA	Nondisplaced subtrochanteric fracture of right femur, initial encounter for closed fracture
S7224XB	Nondisplaced subtrochanteric fracture of right femur, initial encounter for open fracture type I or II
S7224XC	Nondisplaced subtrochanteric fracture of right femur, initial encounter for open fracture type IIIA, IIIB, or IIIC
S7225XA	Nondisplaced subtrochanteric fracture of left femur, initial encounter for closed fracture
S7225XB	Nondisplaced subtrochanteric fracture of left femur, initial encounter for open fracture type I or II
S7225XC	Nondisplaced subtrochanteric fracture of left femur, initial encounter for open fracture type IIIA, IIIB, or IIIC
S7226XA	Nondisplaced subtrochanteric fracture of unspecified femur, initial encounter for closed fracture
S7226XB	Nondisplaced subtrochanteric fracture of unspecified femur, initial encounter for open fracture type I or II
S7226XC	Nondisplaced subtrochanteric fracture of unspecified femur, initial encounter for open fracture type IIIA, IIIB, or IIIC
S72301A	Unspecified fracture of shaft of right femur, initial encounter for closed fracture
S72301B	Unspecified fracture of shaft of right femur, initial encounter for open fracture type I or II
S72301C	Unspecified fracture of shaft of right femur, initial encounter for open fracture type IIIA, IIIB, or IIIC
S72302A	Unspecified fracture of shaft of left femur, initial encounter for closed fracture
S72302B	Unspecified fracture of shaft of left femur, initial encounter for open fracture type I or II
S72302C	Unspecified fracture of shaft of left femur, initial encounter for open fracture type IIIA, IIIB, or IIIC
S72309A	Unspecified fracture of shaft of unspecified femur, initial encounter for closed fracture
S72309B	Unspecified fracture of shaft of unspecified femur, initial encounter for open fracture type I or II
S72309C	Unspecified fracture of shaft of unspecified femur, initial encounter for open fracture type IIIA, IIIB, or IIIC
S72321A	Displaced transverse fracture of shaft of right femur, initial encounter for closed fracture
S72321B	Displaced transverse fracture of shaft of right femur, initial encounter for open fracture type I or II
S72321C	Displaced transverse fracture of shaft of right femur, initial encounter for open fracture type IIIA, IIIB, or IIIC

S72322A	Displaced transverse fracture of shaft of left femur, initial encounter for closed fracture
S72322B	Displaced transverse fracture of shaft of left femur, initial encounter for open fracture type I or II
S72322C	Displaced transverse fracture of shaft of left femur, initial encounter for open fracture type IIIA, IIIB, or IIIC
S72323A	Displaced transverse fracture of shaft of unspecified femur, initial encounter for closed fracture
S72323B	Displaced transverse fracture of shaft of unspecified femur, initial encounter for open fracture type I or II
S72323C	Displaced transverse fracture of shaft of unspecified femur, initial encounter for open fracture type IIIA, IIIB, or IIIC
S72324A	Nondisplaced transverse fracture of shaft of right femur, initial encounter for closed fracture
S72324B	Nondisplaced transverse fracture of shaft of right femur, initial encounter for open fracture type I or II
S72324C	Nondisplaced transverse fracture of shaft of right femur, initial encounter for open fracture type IIIA, IIIB, or IIIC
S72325A	Nondisplaced transverse fracture of shaft of left femur, initial encounter for closed fracture
S72325B	Nondisplaced transverse fracture of shaft of left femur, initial encounter for open fracture type I or II
S72325C	Nondisplaced transverse fracture of shaft of left femur, initial encounter for open fracture type IIIA, IIIB, or IIIC
S72326A	Nondisplaced transverse fracture of shaft of unspecified femur, initial encounter for closed fracture
S72326B	Nondisplaced transverse fracture of shaft of unspecified femur, initial encounter for open fracture type I or II
S72326C	Nondisplaced transverse fracture of shaft of unspecified femur, initial encounter for open fracture type IIIA, IIIB, or IIIC
S72331A	Displaced oblique fracture of shaft of right femur, initial encounter for closed fracture
S72331B	Displaced oblique fracture of shaft of right femur, initial encounter for open fracture type I or II
S72331C	Displaced oblique fracture of shaft of right femur, initial encounter for open fracture type IIIA, IIIB, or IIIC
S72332A	Displaced oblique fracture of shaft of left femur, initial encounter for closed fracture
S72332B	Displaced oblique fracture of shaft of left femur, initial encounter for open fracture type I or II
S72332C	Displaced oblique fracture of shaft of left femur, initial encounter for open fracture type IIIA, IIIB, or IIIC
S72333A	Displaced oblique fracture of shaft of unspecified femur, initial encounter for closed fracture
S72333B	Displaced oblique fracture of shaft of unspecified femur, initial encounter for open fracture type I or II
S72333C	Displaced oblique fracture of shaft of unspecified femur, initial encounter for open fracture type IIIA, IIIB, or IIIC
S72334A	Nondisplaced oblique fracture of shaft of right femur, initial encounter for closed fracture
S72334B	Nondisplaced oblique fracture of shaft of right femur, initial encounter for open fracture type I or II
S72334C	Nondisplaced oblique fracture of shaft of right femur, initial encounter for open fracture type IIIA, IIIB, or IIIC
S72335A	Nondisplaced oblique fracture of shaft of left femur, initial encounter for closed fracture
S72335B	Nondisplaced oblique fracture of shaft of left femur, initial encounter for open fracture type I or II
S72335C	Nondisplaced oblique fracture of shaft of left femur, initial encounter for open fracture type IIIA, IIIB, or IIIC
S72336A	Nondisplaced oblique fracture of shaft of unspecified femur, initial encounter for closed fracture
S72336B	Nondisplaced oblique fracture of shaft of unspecified femur, initial encounter for open fracture type I or II
S72336C	Nondisplaced oblique fracture of shaft of unspecified femur, initial encounter for open fracture type IIIA, IIIB, or IIIC
S72341A	Displaced spiral fracture of shaft of right femur, initial encounter for closed fracture

S72341B	Displaced spiral fracture of shaft of right femur, initial encounter for open fracture type I or II
S72341C	Displaced spiral fracture of shaft of right femur, initial encounter for open fracture type IIIA, IIIB, or IIIC
S72342A	Displaced spiral fracture of shaft of left femur, initial encounter for closed fracture
S72342B	Displaced spiral fracture of shaft of left femur, initial encounter for open fracture type I or II
S72342C	Displaced spiral fracture of shaft of left femur, initial encounter for open fracture type IIIA, IIIB, or IIIC
S72343A	Displaced spiral fracture of shaft of unspecified femur, initial encounter for closed fracture
S72343B	Displaced spiral fracture of shaft of unspecified femur, initial encounter for open fracture type I or II
S72343C	Displaced spiral fracture of shaft of unspecified femur, initial encounter for open fracture type IIIA, IIIB, or IIIC
S72344A	Nondisplaced spiral fracture of shaft of right femur, initial encounter for closed fracture
S72344B	Nondisplaced spiral fracture of shaft of right femur, initial encounter for open fracture type I or II
S72344C	Nondisplaced spiral fracture of shaft of right femur, initial encounter for open fracture type IIIA, IIIB, or IIIC
S72345A	Nondisplaced spiral fracture of shaft of left femur, initial encounter for closed fracture
S72345B	Nondisplaced spiral fracture of shaft of left femur, initial encounter for open fracture type I or II
S72345C	Nondisplaced spiral fracture of shaft of left femur, initial encounter for open fracture type IIIA, IIIB, or IIIC
S72346A	Nondisplaced spiral fracture of shaft of unspecified femur, initial encounter for closed fracture
S72346B	Nondisplaced spiral fracture of shaft of unspecified femur, initial encounter for open fracture type I or II
S72346C	Nondisplaced spiral fracture of shaft of unspecified femur, initial encounter for open fracture type IIIA, IIIB, or IIIC
S72351A	Displaced comminuted fracture of shaft of right femur, initial encounter for closed fracture
S72351B	Displaced comminuted fracture of shaft of right femur, initial encounter for open fracture type I or II
S72351C	Displaced comminuted fracture of shaft of right femur, initial encounter for open fracture type IIIA, IIIB, or IIIC
S72352A	Displaced comminuted fracture of shaft of left femur, initial encounter for closed fracture
S72352B	Displaced comminuted fracture of shaft of left femur, initial encounter for open fracture type I or II
S72352C	Displaced comminuted fracture of shaft of left femur, initial encounter for open fracture type IIIA, IIIB, or IIIC
S72353A	Displaced comminuted fracture of shaft of unspecified femur, initial encounter for closed fracture
S72353B	Displaced comminuted fracture of shaft of unspecified femur, initial encounter for open fracture type I or II
S72353C	Displaced comminuted fracture of shaft of unspecified femur, initial encounter for open fracture type IIIA, IIIB, or IIIC
S72354A	Nondisplaced comminuted fracture of shaft of right femur, initial encounter for closed fracture
S72354B	Nondisplaced comminuted fracture of shaft of right femur, initial encounter for open fracture type I or II
S72354C	Nondisplaced comminuted fracture of shaft of right femur, initial encounter for open fracture type IIIA, IIIB, or IIIC
S72355A	Nondisplaced comminuted fracture of shaft of left femur, initial encounter for closed fracture
S72355B	Nondisplaced comminuted fracture of shaft of left femur, initial encounter for open fracture type I or II
S72355C	Nondisplaced comminuted fracture of shaft of left femur, initial encounter for open fracture type IIIA, IIIB, or IIIC
S72356A	Nondisplaced comminuted fracture of shaft of unspecified femur, initial encounter for closed fracture
S72356B	Nondisplaced comminuted fracture of shaft of unspecified femur, initial encounter for open fracture type I or II

S72356C	Nondisplaced comminuted fracture of shaft of unspecified femur, initial encounter for open fracture type IIIA, IIIB, or IIIC
S72361A	Displaced segmental fracture of shaft of right femur, initial encounter for closed fracture
S72361B	Displaced segmental fracture of shaft of right femur, initial encounter for open fracture type I or II
S72361C	Displaced segmental fracture of shaft of right femur, initial encounter for open fracture type IIIA, IIIB, or IIIC
S72362A	Displaced segmental fracture of shaft of left femur, initial encounter for closed fracture
S72362B	Displaced segmental fracture of shaft of left femur, initial encounter for open fracture type I or II
S72362C	Displaced segmental fracture of shaft of left femur, initial encounter for open fracture type IIIA, IIIB, or IIIC
S72363A	Displaced segmental fracture of shaft of unspecified femur, initial encounter for closed fracture
S72363B	Displaced segmental fracture of shaft of unspecified femur, initial encounter for open fracture type I or II
S72363C	Displaced segmental fracture of shaft of unspecified femur, initial encounter for open fracture type IIIA, IIIB, or IIIC
S72364A	Nondisplaced segmental fracture of shaft of right femur, initial encounter for closed fracture
S72364B	Nondisplaced segmental fracture of shaft of right femur, initial encounter for open fracture type I or II
S72364C	Nondisplaced segmental fracture of shaft of right femur, initial encounter for open fracture type IIIA, IIIB, or IIIC
S72365A	Nondisplaced segmental fracture of shaft of left femur, initial encounter for closed fracture
S72365B	Nondisplaced segmental fracture of shaft of left femur, initial encounter for open fracture type I or II
S72365C	Nondisplaced segmental fracture of shaft of left femur, initial encounter for open fracture type IIIA, IIIB, or IIIC
S72366A	Nondisplaced segmental fracture of shaft of unspecified femur, initial encounter for closed fracture
S72366B	Nondisplaced segmental fracture of shaft of unspecified femur, initial encounter for open fracture type I or II
S72366C	Nondisplaced segmental fracture of shaft of unspecified femur, initial encounter for open fracture type IIIA, IIIB, or IIIC
S72391A	Other fracture of shaft of right femur, initial encounter for closed fracture
S72391B	Other fracture of shaft of right femur, initial encounter for open fracture type I or II
S72391C	Other fracture of shaft of right femur, initial encounter for open fracture type IIIA, IIIB, or IIIC
S72392A	Other fracture of shaft of left femur, initial encounter for closed fracture
S72392B	Other fracture of shaft of left femur, initial encounter for open fracture type I or II
S72392C	Other fracture of shaft of left femur, initial encounter for open fracture type IIIA, IIIB, or IIIC
S72399A	Other fracture of shaft of unspecified femur, initial encounter for closed fracture
S72399B	Other fracture of shaft of unspecified femur, initial encounter for open fracture type I or II
S72399C	Other fracture of shaft of unspecified femur, initial encounter for open fracture type IIIA, IIIB, or IIIC
S72401A	Unspecified fracture of lower end of right femur, initial encounter for closed fracture
S72401B	Unspecified fracture of lower end of right femur, initial encounter for open fracture type I or II
S72401C	Unspecified fracture of lower end of right femur, initial encounter for open fracture type IIIA, IIIB, or IIIC
S72402A	Unspecified fracture of lower end of left femur, initial encounter for closed fracture
S72402B	Unspecified fracture of lower end of left femur, initial encounter for open fracture type I or II
S72402C	Unspecified fracture of lower end of left femur, initial encounter for open fracture type IIIA, IIIB, or IIIC
S72409A	Unspecified fracture of lower end of unspecified femur, initial encounter for closed fracture
S72409B	Unspecified fracture of lower end of unspecified femur, initial encounter for open fracture type I or II

S72409C	Unspecified fracture of lower end of unspecified femur, initial encounter for open fracture type IIIA, IIIB, or IIIC
S72411A	Displaced unspecified condyle fracture of lower end of right femur, initial encounter for closed fracture
S72411B	Displaced unspecified condyle fracture of lower end of right femur, initial encounter for open fracture type I or II
S72411C	Displaced unspecified condyle fracture of lower end of right femur, initial encounter for open fracture type IIIA, IIIB, or IIIC
S72412A	Displaced unspecified condyle fracture of lower end of left femur, initial encounter for closed fracture
S72412B	Displaced unspecified condyle fracture of lower end of left femur, initial encounter for open fracture type I or II
S72412C	Displaced unspecified condyle fracture of lower end of left femur, initial encounter for open fracture type IIIA, IIIB, or IIIC
S72413A	Displaced unspecified condyle fracture of lower end of unspecified femur, initial encounter for closed fracture
S72413B	Displaced unspecified condyle fracture of lower end of unspecified femur, initial encounter for open fracture type I or II
S72413C	Displaced unspecified condyle fracture of lower end of unspecified femur, initial encounter for open fracture type IIIA, IIIB, or IIIC
S72414A	Nondisplaced unspecified condyle fracture of lower end of right femur, initial encounter for closed fracture
S72414B	Nondisplaced unspecified condyle fracture of lower end of right femur, initial encounter for open fracture type I or II
S72414C	Nondisplaced unspecified condyle fracture of lower end of right femur, initial encounter for open fracture type IIIA, IIIB, or IIIC
S72415A	Nondisplaced unspecified condyle fracture of lower end of left femur, initial encounter for closed fracture
S72415B	Nondisplaced unspecified condyle fracture of lower end of left femur, initial encounter for open fracture type I or II
S72415C	Nondisplaced unspecified condyle fracture of lower end of left femur, initial encounter for open fracture type IIIA, IIIB, or IIIC
S72416A	Nondisplaced unspecified condyle fracture of lower end of unspecified femur, initial encounter for closed fracture
S72416B	Nondisplaced unspecified condyle fracture of lower end of unspecified femur, initial encounter for open fracture type I or II
S72416C	Nondisplaced unspecified condyle fracture of lower end of unspecified femur, initial encounter for open fracture type IIIA, IIIB, or IIIC
S72421A	Displaced fracture of lateral condyle of right femur, initial encounter for closed fracture
S72421B	Displaced fracture of lateral condyle of right femur, initial encounter for open fracture type I or II
S72421C	Displaced fracture of lateral condyle of right femur, initial encounter for open fracture type IIIA, IIIB, or IIIC
S72422A	Displaced fracture of lateral condyle of left femur, initial encounter for closed fracture
S72422B	Displaced fracture of lateral condyle of left femur, initial encounter for open fracture type I or II
S72422C	Displaced fracture of lateral condyle of left femur, initial encounter for open fracture type IIIA, IIIB, or IIIC
S72423A	Displaced fracture of lateral condyle of unspecified femur, initial encounter for closed fracture
S72423B	Displaced fracture of lateral condyle of unspecified femur, initial encounter for open fracture type I or II
S72423C	Displaced fracture of lateral condyle of unspecified femur, initial encounter for open fracture type IIIA, IIIB, or IIIC
S72424A	Nondisplaced fracture of lateral condyle of right femur, initial encounter for closed fracture

S72424B	Nondisplaced fracture of lateral condyle of right femur, initial encounter for open fracture type I or II
S72424C	Nondisplaced fracture of lateral condyle of right femur, initial encounter for open fracture type IIIA, IIIB, or IIIC
S72425A	Nondisplaced fracture of lateral condyle of left femur, initial encounter for closed fracture
S72425B	Nondisplaced fracture of lateral condyle of left femur, initial encounter for open fracture type I or II
S72425C	Nondisplaced fracture of lateral condyle of left femur, initial encounter for open fracture type IIIA, IIIB, or IIIC
S72426A	Nondisplaced fracture of lateral condyle of unspecified femur, initial encounter for closed fracture
S72426B	Nondisplaced fracture of lateral condyle of unspecified femur, initial encounter for open fracture type I or II
S72426C	Nondisplaced fracture of lateral condyle of unspecified femur, initial encounter for open fracture type IIIA, IIIB, or IIIC
S72431A	Displaced fracture of medial condyle of right femur, initial encounter for closed fracture
S72431B	Displaced fracture of medial condyle of right femur, initial encounter for open fracture type I or II
S72431C	Displaced fracture of medial condyle of right femur, initial encounter for open fracture type IIIA, IIIB, or IIIC
S72432A	Displaced fracture of medial condyle of left femur, initial encounter for closed fracture
S72432B	Displaced fracture of medial condyle of left femur, initial encounter for open fracture type I or II
S72432C	Displaced fracture of medial condyle of left femur, initial encounter for open fracture type IIIA, IIIB, or IIIC
S72433A	Displaced fracture of medial condyle of unspecified femur, initial encounter for closed fracture
S72433B	Displaced fracture of medial condyle of unspecified femur, initial encounter for open fracture type I or II
S72433C	Displaced fracture of medial condyle of unspecified femur, initial encounter for open fracture type IIIA, IIIB, or IIIC
S72434A	Nondisplaced fracture of medial condyle of right femur, initial encounter for closed fracture
S72434B	Nondisplaced fracture of medial condyle of right femur, initial encounter for open fracture type I or II
S72434C	Nondisplaced fracture of medial condyle of right femur, initial encounter for open fracture type IIIA, IIIB, or IIIC
S72435A	Nondisplaced fracture of medial condyle of left femur, initial encounter for closed fracture
S72435B	Nondisplaced fracture of medial condyle of left femur, initial encounter for open fracture type I or II
S72435C	Nondisplaced fracture of medial condyle of left femur, initial encounter for open fracture type IIIA, IIIB, or IIIC
S72436A	Nondisplaced fracture of medial condyle of unspecified femur, initial encounter for closed fracture
S72436B	Nondisplaced fracture of medial condyle of unspecified femur, initial encounter for open fracture type I or II
S72436C	Nondisplaced fracture of medial condyle of unspecified femur, initial encounter for open fracture type IIIA, IIIB, or IIIC
S72441A	Displaced fracture of lower epiphysis (separation) of right femur, initial encounter for closed fracture
S72441B	Displaced fracture of lower epiphysis (separation) of right femur, initial encounter for open fracture type I or II
S72441C	Displaced fracture of lower epiphysis (separation) of right femur, initial encounter for open fracture type IIIA, IIIB, or IIIC
S72442A	Displaced fracture of lower epiphysis (separation) of left femur, initial encounter for closed fracture
S72442B	Displaced fracture of lower epiphysis (separation) of left femur, initial encounter for open fracture type I or II
S72442C	Displaced fracture of lower epiphysis (separation) of left femur, initial encounter for open fracture type IIIA, IIIB, or IIIC

S72443A	Displaced fracture of lower epiphysis (separation) of unspecified femur, initial encounter for closed fracture
S72443B	Displaced fracture of lower epiphysis (separation) of unspecified femur, initial encounter for open fracture type I or II
S72443C	Displaced fracture of lower epiphysis (separation) of unspecified femur, initial encounter for open fracture type IIIA, IIIB, or IIIC
S72444A	Nondisplaced fracture of lower epiphysis (separation) of right femur, initial encounter for closed fracture
S72444B	Nondisplaced fracture of lower epiphysis (separation) of right femur, initial encounter for open fracture type I or II
S72444C	Nondisplaced fracture of lower epiphysis (separation) of right femur, initial encounter for open fracture type IIIA, IIIB, or IIIC
S72445A	Nondisplaced fracture of lower epiphysis (separation) of left femur, initial encounter for closed fracture
S72445B	Nondisplaced fracture of lower epiphysis (separation) of left femur, initial encounter for open fracture type I or II
S72445C	Nondisplaced fracture of lower epiphysis (separation) of left femur, initial encounter for open fracture type IIIA, IIIB, or IIIC
S72446A	Nondisplaced fracture of lower epiphysis (separation) of unspecified femur, initial encounter for closed fracture
S72446B	Nondisplaced fracture of lower epiphysis (separation) of unspecified femur, initial encounter for open fracture type I or II
S72446C	Nondisplaced fracture of lower epiphysis (separation) of unspecified femur, initial encounter for open fracture type IIIA, IIIB, or IIIC
S72451A	Displaced supracondylar fracture without intracondylar extension of lower end of right femur, initial encounter for closed fracture
S72451B	Displaced supracondylar fracture without intracondylar extension of lower end of right femur, initial encounter for open fracture type I or II
S72451C	Displaced supracondylar fracture without intracondylar extension of lower end of right femur, initial encounter for open fracture type IIIA, IIIB, or IIIC
S72452A	Displaced supracondylar fracture without intracondylar extension of lower end of left femur, initial encounter for closed fracture
S72452B	Displaced supracondylar fracture without intracondylar extension of lower end of left femur, initial encounter for open fracture type I or II
S72452C	Displaced supracondylar fracture without intracondylar extension of lower end of left femur, initial encounter for open fracture type IIIA, IIIB, or IIIC
S72453A	Displaced supracondylar fracture without intracondylar extension of lower end of unspecified femur, initial encounter for closed fracture
S72453B	Displaced supracondylar fracture without intracondylar extension of lower end of unspecified femur, initial encounter for open fracture type I or II
S72453C	Displaced supracondylar fracture without intracondylar extension of lower end of unspecified femur, initial encounter for open fracture type IIIA, IIIB, or IIIC
S72454A	Nondisplaced supracondylar fracture without intracondylar extension of lower end of right femur, initial encounter for closed fracture
S72454B	Nondisplaced supracondylar fracture without intracondylar extension of lower end of right femur, initial encounter for open fracture type I or II
S72454C	Nondisplaced supracondylar fracture without intracondylar extension of lower end of right femur, initial encounter for open fracture type IIIA, IIIB, or IIIC
S72455A	Nondisplaced supracondylar fracture without intracondylar extension of lower end of left femur, initial encounter for closed fracture
S72455B	Nondisplaced supracondylar fracture without intracondylar extension of lower end of left femur, initial

	encounter for open fracture type I or II
S72455C	Nondisplaced supracondylar fracture without intracondylar extension of lower end of left femur, initial encounter for open fracture type IIIA, IIIB, or IIIC
S72456A	Nondisplaced supracondylar fracture without intracondylar extension of lower end of unspecified femur, initial encounter for closed fracture
S72456B	Nondisplaced supracondylar fracture without intracondylar extension of lower end of unspecified femur, initial encounter for open fracture type I or II
S72456C	Nondisplaced supracondylar fracture without intracondylar extension of lower end of unspecified femur, initial encounter for open fracture type IIIA, IIIB, or IIIC
S72461A	Displaced supracondylar fracture with intracondylar extension of lower end of right femur, initial encounter for closed fracture
S72461B	Displaced supracondylar fracture with intracondylar extension of lower end of right femur, initial encounter for open fracture type I or II
S72461C	Displaced supracondylar fracture with intracondylar extension of lower end of right femur, initial encounter for open fracture type IIIA, IIIB, or IIIC
S72462A	Displaced supracondylar fracture with intracondylar extension of lower end of left femur, initial encounter for closed fracture
S72462B	Displaced supracondylar fracture with intracondylar extension of lower end of left femur, initial encounter for open fracture type I or II
S72462C	Displaced supracondylar fracture with intracondylar extension of lower end of left femur, initial encounter for open fracture type IIIA, IIIB, or IIIC
S72463A	Displaced supracondylar fracture with intracondylar extension of lower end of unspecified femur, initial encounter for closed fracture
S72463B	Displaced supracondylar fracture with intracondylar extension of lower end of unspecified femur, initial encounter for open fracture type I or II
S72463C	Displaced supracondylar fracture with intracondylar extension of lower end of unspecified femur, initial encounter for open fracture type IIIA, IIIB, or IIIC
S72464A	Nondisplaced supracondylar fracture with intracondylar extension of lower end of right femur, initial encounter for closed fracture
S72464B	Nondisplaced supracondylar fracture with intracondylar extension of lower end of right femur, initial encounter for open fracture type I or II
S72464C	Nondisplaced supracondylar fracture with intracondylar extension of lower end of right femur, initial encounter for open fracture type IIIA, IIIB, or IIIC
S72465A	Nondisplaced supracondylar fracture with intracondylar extension of lower end of left femur, initial encounter for closed fracture
S72465B	Nondisplaced supracondylar fracture with intracondylar extension of lower end of left femur, initial encounter for open fracture type I or II
S72465C	Nondisplaced supracondylar fracture with intracondylar extension of lower end of left femur, initial encounter for open fracture type IIIA, IIIB, or IIIC
S72466A	Nondisplaced supracondylar fracture with intracondylar extension of lower end of unspecified femur, initial encounter for closed fracture
S72466B	Nondisplaced supracondylar fracture with intracondylar extension of lower end of unspecified femur, initial encounter for open fracture type I or II
S72466C	Nondisplaced supracondylar fracture with intracondylar extension of lower end of unspecified femur, initial encounter for open fracture type IIIA, IIIB, or IIIC
S72471A	Torus fracture of lower end of right femur, initial encounter for closed fracture
S72472A	Torus fracture of lower end of left femur, initial encounter for closed fracture
S72479A	Torus fracture of lower end of unspecified femur, initial encounter for closed fracture
S72491A	Other fracture of lower end of right femur, initial encounter for closed fracture
S72491B	Other fracture of lower end of right femur, initial encounter for open fracture type I or II

S72491C	Other fracture of lower end of right femur, initial encounter for open fracture type IIIA, IIIB, or IIIC
S72492A	Other fracture of lower end of left femur, initial encounter for closed fracture
S72492B	Other fracture of lower end of left femur, initial encounter for open fracture type I or II
S72492C	Other fracture of lower end of left femur, initial encounter for open fracture type IIIA, IIIB, or IIIC
S72499A	Other fracture of lower end of unspecified femur, initial encounter for closed fracture
S72499B	Other fracture of lower end of unspecified femur, initial encounter for open fracture type I or II
S72499C	Other fracture of lower end of unspecified femur, initial encounter for open fracture type IIIA, IIIB, or IIIC
S728X1A	Other fracture of right femur, initial encounter for closed fracture
S728X1B	Other fracture of right femur, initial encounter for open fracture type I or II
S728X1C	Other fracture of right femur, initial encounter for open fracture type IIIA, IIIB, or IIIC
S728X2A	Other fracture of left femur, initial encounter for closed fracture
S728X2B	Other fracture of left femur, initial encounter for open fracture type I or II
S728X2C	Other fracture of left femur, initial encounter for open fracture type IIIA, IIIB, or IIIC
S728X9A	Other fracture of unspecified femur, initial encounter for closed fracture
S728X9B	Other fracture of unspecified femur, initial encounter for open fracture type I or II
S728X9C	Other fracture of unspecified femur, initial encounter for open fracture type IIIA, IIIB, or IIIC
S7290XA	Unspecified fracture of unspecified femur, initial encounter for closed fracture
S7290XB	Unspecified fracture of unspecified femur, initial encounter for open fracture type I or II
S7290XC	Unspecified fracture of unspecified femur, initial encounter for open fracture type IIIA, IIIB, or IIIC
S7291XA	Unspecified fracture of right femur, initial encounter for closed fracture
S7291XB	Unspecified fracture of right femur, initial encounter for open fracture type I or II
S7291XC	Unspecified fracture of right femur, initial encounter for open fracture type IIIA, IIIB, or IIIC
S7292XA	Unspecified fracture of left femur, initial encounter for closed fracture
S7292XB	Unspecified fracture of left femur, initial encounter for open fracture type I or II
S7292XC	Unspecified fracture of left femur, initial encounter for open fracture type IIIA, IIIB, or IIIC
S79001A	Unspecified physeal fracture of upper end of right femur, initial encounter for closed fracture
S79002A	Unspecified physeal fracture of upper end of left femur, initial encounter for closed fracture
S79009A	Unspecified physeal fracture of upper end of unspecified femur, initial encounter for closed fracture
S79011A	Salter-Harris Type I physeal fracture of upper end of right femur, initial encounter for closed fracture
S79012A	Salter-Harris Type I physeal fracture of upper end of left femur, initial encounter for closed fracture
S79019A	Salter-Harris Type I physeal fracture of upper end of unspecified femur, initial encounter for closed fracture
S79091A	Other physeal fracture of upper end of right femur, initial encounter for closed fracture
S79092A	Other physeal fracture of upper end of left femur, initial encounter for closed fracture
S79099A	Other physeal fracture of upper end of unspecified femur, initial encounter for closed fracture
S79101A	Unspecified physeal fracture of lower end of right femur, initial encounter for closed fracture
S79102A	Unspecified physeal fracture of lower end of left femur, initial encounter for closed fracture
S79109A	Unspecified physeal fracture of lower end of unspecified femur, initial encounter for closed fracture
S79111A	Salter-Harris Type I physeal fracture of lower end of right femur, initial encounter for closed fracture
S79112A	Salter-Harris Type I physeal fracture of lower end of left femur, initial encounter for closed fracture
S79119A	Salter-Harris Type I physeal fracture of lower end of unspecified femur, initial encounter for closed fracture
S79121A	Salter-Harris Type II physeal fracture of lower end of right femur, initial encounter for closed fracture

S79122A	Salter-Harris Type II physeal fracture of lower end of left femur, initial encounter for closed fracture
S79129A	Salter-Harris Type II physeal fracture of lower end of unspecified femur, initial encounter for closed fracture
S79131A	Salter-Harris Type III physeal fracture of lower end of right femur, initial encounter for closed fracture
S79132A	Salter-Harris Type III physeal fracture of lower end of left femur, initial encounter for closed fracture
S79139A	Salter-Harris Type III physeal fracture of lower end of unspecified femur, initial encounter for closed fracture
S79141A	Salter-Harris Type IV physeal fracture of lower end of right femur, initial encounter for closed fracture
S79142A	Salter-Harris Type IV physeal fracture of lower end of left femur, initial encounter for closed fracture
S79149A	Salter-Harris Type IV physeal fracture of lower end of unspecified femur, initial encounter for closed fracture
S79191A	Other physeal fracture of lower end of right femur, initial encounter for closed fracture
S79192A	Other physeal fracture of lower end of left femur, initial encounter for closed fracture
S79199A	Other physeal fracture of lower end of unspecified femur, initial encounter for closed fracture
S82101A	Unspecified fracture of upper end of right tibia, initial encounter for closed fracture
S82101B	Unspecified fracture of upper end of right tibia, initial encounter for open fracture type I or II
S82101C	Unspecified fracture of upper end of right tibia, initial encounter for open fracture type IIIA, IIIB, or IIIC
S82102A	Unspecified fracture of upper end of left tibia, initial encounter for closed fracture
S82102B	Unspecified fracture of upper end of left tibia, initial encounter for open fracture type I or II
S82102C	Unspecified fracture of upper end of left tibia, initial encounter for open fracture type IIIA, IIIB, or IIIC
S82109A	Unspecified fracture of upper end of unspecified tibia, initial encounter for closed fracture
S82109B	Unspecified fracture of upper end of unspecified tibia, initial encounter for open fracture type I or II
S82109C	Unspecified fracture of upper end of unspecified tibia, initial encounter for open fracture type IIIA, IIIB, or IIIC
S82111A	Displaced fracture of right tibial spine, initial encounter for closed fracture
S82111B	Displaced fracture of right tibial spine, initial encounter for open fracture type I or II
S82111C	Displaced fracture of right tibial spine, initial encounter for open fracture type IIIA, IIIB, or IIIC
S82112A	Displaced fracture of left tibial spine, initial encounter for closed fracture
S82112B	Displaced fracture of left tibial spine, initial encounter for open fracture type I or II
S82112C	Displaced fracture of left tibial spine, initial encounter for open fracture type IIIA, IIIB, or IIIC
S82113A	Displaced fracture of unspecified tibial spine, initial encounter for closed fracture
S82113B	Displaced fracture of unspecified tibial spine, initial encounter for open fracture type I or II
S82113C	Displaced fracture of unspecified tibial spine, initial encounter for open fracture type IIIA, IIIB, or IIIC
S82114A	Nondisplaced fracture of right tibial spine, initial encounter for closed fracture
S82114B	Nondisplaced fracture of right tibial spine, initial encounter for open fracture type I or II
S82114C	Nondisplaced fracture of right tibial spine, initial encounter for open fracture type IIIA, IIIB, or IIIC
S82115A	Nondisplaced fracture of left tibial spine, initial encounter for closed fracture
S82115B	Nondisplaced fracture of left tibial spine, initial encounter for open fracture type I or II
S82115C	Nondisplaced fracture of left tibial spine, initial encounter for open fracture type IIIA, IIIB, or IIIC
S82116A	Nondisplaced fracture of unspecified tibial spine, initial encounter for closed fracture
S82116B	Nondisplaced fracture of unspecified tibial spine, initial encounter for open fracture type I or II
S82116C	Nondisplaced fracture of unspecified tibial spine, initial encounter for open fracture type IIIA, IIIB, or IIIC
S82121A	Displaced fracture of lateral condyle of right tibia, initial encounter for closed fracture
S82121B	Displaced fracture of lateral condyle of right tibia, initial encounter for open fracture type I or II

S82121C	Displaced fracture of lateral condyle of right tibia, initial encounter for open fracture type IIIA, IIIB, or IIIC
S82122A	Displaced fracture of lateral condyle of left tibia, initial encounter for closed fracture
S82122B	Displaced fracture of lateral condyle of left tibia, initial encounter for open fracture type I or II
S82122C	Displaced fracture of lateral condyle of left tibia, initial encounter for open fracture type IIIA, IIIB, or IIIC
S82123A	Displaced fracture of lateral condyle of unspecified tibia, initial encounter for closed fracture
S82123B	Displaced fracture of lateral condyle of unspecified tibia, initial encounter for open fracture type I or II
S82123C	Displaced fracture of lateral condyle of unspecified tibia, initial encounter for open fracture type IIIA, IIIB, or IIIC
S82124A	Nondisplaced fracture of lateral condyle of right tibia, initial encounter for closed fracture
S82124B	Nondisplaced fracture of lateral condyle of right tibia, initial encounter for open fracture type I or II
S82124C	Nondisplaced fracture of lateral condyle of right tibia, initial encounter for open fracture type IIIA, IIIB, or IIIC
S82125A	Nondisplaced fracture of lateral condyle of left tibia, initial encounter for closed fracture
S82125B	Nondisplaced fracture of lateral condyle of left tibia, initial encounter for open fracture type I or II
S82125C	Nondisplaced fracture of lateral condyle of left tibia, initial encounter for open fracture type IIIA, IIIB, or IIIC
S82126A	Nondisplaced fracture of lateral condyle of unspecified tibia, initial encounter for closed fracture
S82126B	Nondisplaced fracture of lateral condyle of unspecified tibia, initial encounter for open fracture type I or II
S82126C	Nondisplaced fracture of lateral condyle of unspecified tibia, initial encounter for open fracture type IIIA, IIIB, or IIIC
S82131A	Displaced fracture of medial condyle of right tibia, initial encounter for closed fracture
S82131B	Displaced fracture of medial condyle of right tibia, initial encounter for open fracture type I or II
S82131C	Displaced fracture of medial condyle of right tibia, initial encounter for open fracture type IIIA, IIIB, or IIIC
S82132A	Displaced fracture of medial condyle of left tibia, initial encounter for closed fracture
S82132B	Displaced fracture of medial condyle of left tibia, initial encounter for open fracture type I or II
S82132C	Displaced fracture of medial condyle of left tibia, initial encounter for open fracture type IIIA, IIIB, or IIIC
S82133A	Displaced fracture of medial condyle of unspecified tibia, initial encounter for closed fracture
S82133B	Displaced fracture of medial condyle of unspecified tibia, initial encounter for open fracture type I or II
S82133C	Displaced fracture of medial condyle of unspecified tibia, initial encounter for open fracture type IIIA, IIIB, or IIIC
S82134A	Nondisplaced fracture of medial condyle of right tibia, initial encounter for closed fracture
S82134B	Nondisplaced fracture of medial condyle of right tibia, initial encounter for open fracture type I or II
S82134C	Nondisplaced fracture of medial condyle of right tibia, initial encounter for open fracture type IIIA, IIIB, or IIIC
S82135A	Nondisplaced fracture of medial condyle of left tibia, initial encounter for closed fracture
S82135B	Nondisplaced fracture of medial condyle of left tibia, initial encounter for open fracture type I or II
S82135C	Nondisplaced fracture of medial condyle of left tibia, initial encounter for open fracture type IIIA, IIIB, or IIIC
S82136A	Nondisplaced fracture of medial condyle of unspecified tibia, initial encounter for closed fracture
S82136B	Nondisplaced fracture of medial condyle of unspecified tibia, initial encounter for open fracture type I or II
S82136C	Nondisplaced fracture of medial condyle of unspecified tibia, initial encounter for open fracture type IIIA, IIIB, or IIIC

S82141A	Displaced bicondylar fracture of right tibia, initial encounter for closed fracture
S82141B	Displaced bicondylar fracture of right tibia, initial encounter for open fracture type I or II
S82141C	Displaced bicondylar fracture of right tibia, initial encounter for open fracture type IIIA, IIIB, or IIIC
S82142A	Displaced bicondylar fracture of left tibia, initial encounter for closed fracture
S82142B	Displaced bicondylar fracture of left tibia, initial encounter for open fracture type I or II
S82142C	Displaced bicondylar fracture of left tibia, initial encounter for open fracture type IIIA, IIIB, or IIIC
S82143A	Displaced bicondylar fracture of unspecified tibia, initial encounter for closed fracture
S82143B	Displaced bicondylar fracture of unspecified tibia, initial encounter for open fracture type I or II
S82143C	Displaced bicondylar fracture of unspecified tibia, initial encounter for open fracture type IIIA, IIIB, or IIIC
S82144A	Nondisplaced bicondylar fracture of right tibia, initial encounter for closed fracture
S82144B	Nondisplaced bicondylar fracture of right tibia, initial encounter for open fracture type I or II
S82144C	Nondisplaced bicondylar fracture of right tibia, initial encounter for open fracture type IIIA, IIIB, or IIIC
S82145A	Nondisplaced bicondylar fracture of left tibia, initial encounter for closed fracture
S82145B	Nondisplaced bicondylar fracture of left tibia, initial encounter for open fracture type I or II
S82145C	Nondisplaced bicondylar fracture of left tibia, initial encounter for open fracture type IIIA, IIIB, or IIIC
S82146A	Nondisplaced bicondylar fracture of unspecified tibia, initial encounter for closed fracture
S82146B	Nondisplaced bicondylar fracture of unspecified tibia, initial encounter for open fracture type I or II
S82146C	Nondisplaced bicondylar fracture of unspecified tibia, initial encounter for open fracture type IIIA, IIIB, or IIIC
S82151A	Displaced fracture of right tibial tuberosity, initial encounter for closed fracture
S82151B	Displaced fracture of right tibial tuberosity, initial encounter for open fracture type I or II
S82151C	Displaced fracture of right tibial tuberosity, initial encounter for open fracture type IIIA, IIIB, or IIIC
S82152A	Displaced fracture of left tibial tuberosity, initial encounter for closed fracture
S82152B	Displaced fracture of left tibial tuberosity, initial encounter for open fracture type I or II
S82152C	Displaced fracture of left tibial tuberosity, initial encounter for open fracture type IIIA, IIIB, or IIIC
S82153A	Displaced fracture of unspecified tibial tuberosity, initial encounter for closed fracture
S82153B	Displaced fracture of unspecified tibial tuberosity, initial encounter for open fracture type I or II
S82153C	Displaced fracture of unspecified tibial tuberosity, initial encounter for open fracture type IIIA, IIIB, or IIIC
S82154A	Nondisplaced fracture of right tibial tuberosity, initial encounter for closed fracture
S82154B	Nondisplaced fracture of right tibial tuberosity, initial encounter for open fracture type I or II
S82154C	Nondisplaced fracture of right tibial tuberosity, initial encounter for open fracture type IIIA, IIIB, or IIIC
S82155A	Nondisplaced fracture of left tibial tuberosity, initial encounter for closed fracture
S82155B	Nondisplaced fracture of left tibial tuberosity, initial encounter for open fracture type I or II
S82155C	Nondisplaced fracture of left tibial tuberosity, initial encounter for open fracture type IIIA, IIIB, or IIIC
S82156A	Nondisplaced fracture of unspecified tibial tuberosity, initial encounter for closed fracture
S82156B	Nondisplaced fracture of unspecified tibial tuberosity, initial encounter for open fracture type I or II
S82156C	Nondisplaced fracture of unspecified tibial tuberosity, initial encounter for open fracture type IIIA, IIIB, or IIIC
S82161A	Torus fracture of upper end of right tibia, initial encounter for closed fracture
S82162A	Torus fracture of upper end of left tibia, initial encounter for closed fracture
S82169A	Torus fracture of upper end of unspecified tibia, initial encounter for closed fracture
S82191A	Other fracture of upper end of right tibia, initial encounter for closed fracture

S82191B	Other fracture of upper end of right tibia, initial encounter for open fracture type I or II
S82191C	Other fracture of upper end of right tibia, initial encounter for open fracture type IIIA, IIIB, or IIIC
S82192A	Other fracture of upper end of left tibia, initial encounter for closed fracture
S82192B	Other fracture of upper end of left tibia, initial encounter for open fracture type I or II
S82192C	Other fracture of upper end of left tibia, initial encounter for open fracture type IIIA, IIIB, or IIIC
S82199A	Other fracture of upper end of unspecified tibia, initial encounter for closed fracture
S82199B	Other fracture of upper end of unspecified tibia, initial encounter for open fracture type I or II
S82199C	Other fracture of upper end of unspecified tibia, initial encounter for open fracture type IIIA, IIIB, or IIIC
S82201A	Unspecified fracture of shaft of right tibia, initial encounter for closed fracture
S82201B	Unspecified fracture of shaft of right tibia, initial encounter for open fracture type I or II
S82201C	Unspecified fracture of shaft of right tibia, initial encounter for open fracture type IIIA, IIIB, or IIIC
S82202A	Unspecified fracture of shaft of left tibia, initial encounter for closed fracture
S82202B	Unspecified fracture of shaft of left tibia, initial encounter for open fracture type I or II
S82202C	Unspecified fracture of shaft of left tibia, initial encounter for open fracture type IIIA, IIIB, or IIIC
S82209A	Unspecified fracture of shaft of unspecified tibia, initial encounter for closed fracture
S82209B	Unspecified fracture of shaft of unspecified tibia, initial encounter for open fracture type I or II
S82209C	Unspecified fracture of shaft of unspecified tibia, initial encounter for open fracture type IIIA, IIIB, or IIIC
S82221A	Displaced transverse fracture of shaft of right tibia, initial encounter for closed fracture
S82221B	Displaced transverse fracture of shaft of right tibia, initial encounter for open fracture type I or II
S82221C	Displaced transverse fracture of shaft of right tibia, initial encounter for open fracture type IIIA, IIIB, or IIIC
S82222A	Displaced transverse fracture of shaft of left tibia, initial encounter for closed fracture
S82222B	Displaced transverse fracture of shaft of left tibia, initial encounter for open fracture type I or II
S82222C	Displaced transverse fracture of shaft of left tibia, initial encounter for open fracture type IIIA, IIIB, or IIIC
S82223A	Displaced transverse fracture of shaft of unspecified tibia, initial encounter for closed fracture
S82223B	Displaced transverse fracture of shaft of unspecified tibia, initial encounter for open fracture type I or II
S82223C	Displaced transverse fracture of shaft of unspecified tibia, initial encounter for open fracture type IIIA, IIIB, or IIIC
S82224A	Nondisplaced transverse fracture of shaft of right tibia, initial encounter for closed fracture
S82224B	Nondisplaced transverse fracture of shaft of right tibia, initial encounter for open fracture type I or II
S82224C	Nondisplaced transverse fracture of shaft of right tibia, initial encounter for open fracture type IIIA, IIIB, or IIIC
S82225A	Nondisplaced transverse fracture of shaft of left tibia, initial encounter for closed fracture
S82225B	Nondisplaced transverse fracture of shaft of left tibia, initial encounter for open fracture type I or II
S82225C	Nondisplaced transverse fracture of shaft of left tibia, initial encounter for open fracture type IIIA, IIIB, or IIIC
S82226A	Nondisplaced transverse fracture of shaft of unspecified tibia, initial encounter for closed fracture
S82226B	Nondisplaced transverse fracture of shaft of unspecified tibia, initial encounter for open fracture type I or II
S82226C	Nondisplaced transverse fracture of shaft of unspecified tibia, initial encounter for open fracture type IIIA, IIIB, or IIIC
S82231A	Displaced oblique fracture of shaft of right tibia, initial encounter for closed fracture

S82231B	Displaced oblique fracture of shaft of right tibia, initial encounter for open fracture type I or II
S82231C	Displaced oblique fracture of shaft of right tibia, initial encounter for open fracture type IIIA, IIIB, or IIIC
S82232A	Displaced oblique fracture of shaft of left tibia, initial encounter for closed fracture
S82232B	Displaced oblique fracture of shaft of left tibia, initial encounter for open fracture type I or II
S82232C	Displaced oblique fracture of shaft of left tibia, initial encounter for open fracture type IIIA, IIIB, or IIIC
S82233A	Displaced oblique fracture of shaft of unspecified tibia, initial encounter for closed fracture
S82233B	Displaced oblique fracture of shaft of unspecified tibia, initial encounter for open fracture type I or II
S82233C	Displaced oblique fracture of shaft of unspecified tibia, initial encounter for open fracture type IIIA, IIIB, or IIIC
S82234A	Nondisplaced oblique fracture of shaft of right tibia, initial encounter for closed fracture
S82234B	Nondisplaced oblique fracture of shaft of right tibia, initial encounter for open fracture type I or II
S82234C	Nondisplaced oblique fracture of shaft of right tibia, initial encounter for open fracture type IIIA, IIIB, or IIIC
S82235A	Nondisplaced oblique fracture of shaft of left tibia, initial encounter for closed fracture
S82235B	Nondisplaced oblique fracture of shaft of left tibia, initial encounter for open fracture type I or II
S82235C	Nondisplaced oblique fracture of shaft of left tibia, initial encounter for open fracture type IIIA, IIIB, or IIIC
S82236A	Nondisplaced oblique fracture of shaft of unspecified tibia, initial encounter for closed fracture
S82236B	Nondisplaced oblique fracture of shaft of unspecified tibia, initial encounter for open fracture type I or II
S82236C	Nondisplaced oblique fracture of shaft of unspecified tibia, initial encounter for open fracture type IIIA, IIIB, or IIIC
S82241A	Displaced spiral fracture of shaft of right tibia, initial encounter for closed fracture
S82241B	Displaced spiral fracture of shaft of right tibia, initial encounter for open fracture type I or II
S82241C	Displaced spiral fracture of shaft of right tibia, initial encounter for open fracture type IIIA, IIIB, or IIIC
S82242A	Displaced spiral fracture of shaft of left tibia, initial encounter for closed fracture
S82242B	Displaced spiral fracture of shaft of left tibia, initial encounter for open fracture type I or II
S82242C	Displaced spiral fracture of shaft of left tibia, initial encounter for open fracture type IIIA, IIIB, or IIIC
S82243A	Displaced spiral fracture of shaft of unspecified tibia, initial encounter for closed fracture
S82243B	Displaced spiral fracture of shaft of unspecified tibia, initial encounter for open fracture type I or II
S82243C	Displaced spiral fracture of shaft of unspecified tibia, initial encounter for open fracture type IIIA, IIIB, or IIIC
S82244A	Nondisplaced spiral fracture of shaft of right tibia, initial encounter for closed fracture
S82244B	Nondisplaced spiral fracture of shaft of right tibia, initial encounter for open fracture type I or II
S82244C	Nondisplaced spiral fracture of shaft of right tibia, initial encounter for open fracture type IIIA, IIIB, or IIIC
S82245A	Nondisplaced spiral fracture of shaft of left tibia, initial encounter for closed fracture
S82245B	Nondisplaced spiral fracture of shaft of left tibia, initial encounter for open fracture type I or II
S82245C	Nondisplaced spiral fracture of shaft of left tibia, initial encounter for open fracture type IIIA, IIIB, or IIIC
S82246A	Nondisplaced spiral fracture of shaft of unspecified tibia, initial encounter for closed fracture
S82246B	Nondisplaced spiral fracture of shaft of unspecified tibia, initial encounter for open fracture type I or II
S82246C	Nondisplaced spiral fracture of shaft of unspecified tibia, initial encounter for open fracture type IIIA, IIIB, or IIIC
S82251A	Displaced comminuted fracture of shaft of right tibia, initial encounter for closed fracture
S82251B	Displaced comminuted fracture of shaft of right tibia, initial encounter for open fracture type I or II

S82251C	Displaced comminuted fracture of shaft of right tibia, initial encounter for open fracture type IIIA, IIIB, or IIIC
S82252A	Displaced comminuted fracture of shaft of left tibia, initial encounter for closed fracture
S82252B	Displaced comminuted fracture of shaft of left tibia, initial encounter for open fracture type I or II
S82252C	Displaced comminuted fracture of shaft of left tibia, initial encounter for open fracture type IIIA, IIIB, or IIIC
S82253A	Displaced comminuted fracture of shaft of unspecified tibia, initial encounter for closed fracture
S82253B	Displaced comminuted fracture of shaft of unspecified tibia, initial encounter for open fracture type I or II
S82253C	Displaced comminuted fracture of shaft of unspecified tibia, initial encounter for open fracture type IIIA, IIIB, or IIIC
S82254A	Nondisplaced comminuted fracture of shaft of right tibia, initial encounter for closed fracture
S82254B	Nondisplaced comminuted fracture of shaft of right tibia, initial encounter for open fracture type I or II
S82254C	Nondisplaced comminuted fracture of shaft of right tibia, initial encounter for open fracture type IIIA, IIIB, or IIIC
S82255A	Nondisplaced comminuted fracture of shaft of left tibia, initial encounter for closed fracture
S82255B	Nondisplaced comminuted fracture of shaft of left tibia, initial encounter for open fracture type I or II
S82255C	Nondisplaced comminuted fracture of shaft of left tibia, initial encounter for open fracture type IIIA, IIIB, or IIIC
S82256A	Nondisplaced comminuted fracture of shaft of unspecified tibia, initial encounter for closed fracture
S82256B	Nondisplaced comminuted fracture of shaft of unspecified tibia, initial encounter for open fracture type I or II
S82256C	Nondisplaced comminuted fracture of shaft of unspecified tibia, initial encounter for open fracture type IIIA, IIIB, or IIIC
S82261A	Displaced segmental fracture of shaft of right tibia, initial encounter for closed fracture
S82261B	Displaced segmental fracture of shaft of right tibia, initial encounter for open fracture type I or II
S82261C	Displaced segmental fracture of shaft of right tibia, initial encounter for open fracture type IIIA, IIIB, or IIIC
S82262A	Displaced segmental fracture of shaft of left tibia, initial encounter for closed fracture
S82262B	Displaced segmental fracture of shaft of left tibia, initial encounter for open fracture type I or II
S82262C	Displaced segmental fracture of shaft of left tibia, initial encounter for open fracture type IIIA, IIIB, or IIIC
S82263A	Displaced segmental fracture of shaft of unspecified tibia, initial encounter for closed fracture
S82263B	Displaced segmental fracture of shaft of unspecified tibia, initial encounter for open fracture type I or II
S82263C	Displaced segmental fracture of shaft of unspecified tibia, initial encounter for open fracture type IIIA, IIIB, or IIIC
S82264A	Nondisplaced segmental fracture of shaft of right tibia, initial encounter for closed fracture
S82264B	Nondisplaced segmental fracture of shaft of right tibia, initial encounter for open fracture type I or II
S82264C	Nondisplaced segmental fracture of shaft of right tibia, initial encounter for open fracture type IIIA, IIIB, or IIIC
S82265A	Nondisplaced segmental fracture of shaft of left tibia, initial encounter for closed fracture
S82265B	Nondisplaced segmental fracture of shaft of left tibia, initial encounter for open fracture type I or II
S82265C	Nondisplaced segmental fracture of shaft of left tibia, initial encounter for open fracture type IIIA, IIIB, or IIIC
S82266A	Nondisplaced segmental fracture of shaft of unspecified tibia, initial encounter for closed fracture
S82266B	Nondisplaced segmental fracture of shaft of unspecified tibia, initial encounter for open fracture type I or II
S82266C	Nondisplaced segmental fracture of shaft of unspecified tibia, initial encounter for open fracture type

	IIIA, IIIB, or IIIC
S82291A	Other fracture of shaft of right tibia, initial encounter for closed fracture
S82291B	Other fracture of shaft of right tibia, initial encounter for open fracture type I or II
S82291C	Other fracture of shaft of right tibia, initial encounter for open fracture type IIIA, IIIB, or IIIC
S82292A	Other fracture of shaft of left tibia, initial encounter for closed fracture
S82292B	Other fracture of shaft of left tibia, initial encounter for open fracture type I or II
S82292C	Other fracture of shaft of left tibia, initial encounter for open fracture type IIIA, IIIB, or IIIC
S82299A	Other fracture of shaft of unspecified tibia, initial encounter for closed fracture
S82299B	Other fracture of shaft of unspecified tibia, initial encounter for open fracture type I or II
S82299C	Other fracture of shaft of unspecified tibia, initial encounter for open fracture type IIIA, IIIB, or IIIC
S82301A	Unspecified fracture of lower end of right tibia, initial encounter for closed fracture
S82301B	Unspecified fracture of lower end of right tibia, initial encounter for open fracture type I or II
S82301C	Unspecified fracture of lower end of right tibia, initial encounter for open fracture type IIIA, IIIB, or IIIC
S82302A	Unspecified fracture of lower end of left tibia, initial encounter for closed fracture
S82302B	Unspecified fracture of lower end of left tibia, initial encounter for open fracture type I or II
S82302C	Unspecified fracture of lower end of left tibia, initial encounter for open fracture type IIIA, IIIB, or IIIC
S82309A	Unspecified fracture of lower end of unspecified tibia, initial encounter for closed fracture
S82309B	Unspecified fracture of lower end of unspecified tibia, initial encounter for open fracture type I or II
S82309C	Unspecified fracture of lower end of unspecified tibia, initial encounter for open fracture type IIIA, IIIB, or IIIC
S82311A	Torus fracture of lower end of right tibia, initial encounter for closed fracture
S82312A	Torus fracture of lower end of left tibia, initial encounter for closed fracture
S82319A	Torus fracture of lower end of unspecified tibia, initial encounter for closed fracture
S82391A	Other fracture of lower end of right tibia, initial encounter for closed fracture
S82391B	Other fracture of lower end of right tibia, initial encounter for open fracture type I or II
S82391C	Other fracture of lower end of right tibia, initial encounter for open fracture type IIIA, IIIB, or IIIC
S82392A	Other fracture of lower end of left tibia, initial encounter for closed fracture
S82392B	Other fracture of lower end of left tibia, initial encounter for open fracture type I or II
S82392C	Other fracture of lower end of left tibia, initial encounter for open fracture type IIIA, IIIB, or IIIC
S82399A	Other fracture of lower end of unspecified tibia, initial encounter for closed fracture
S82399B	Other fracture of lower end of unspecified tibia, initial encounter for open fracture type I or II
S82399C	Other fracture of lower end of unspecified tibia, initial encounter for open fracture type IIIA, IIIB, or IIIC
S82401A	Unspecified fracture of shaft of right fibula, initial encounter for closed fracture
S82401B	Unspecified fracture of shaft of right fibula, initial encounter for open fracture type I or II
S82401C	Unspecified fracture of shaft of right fibula, initial encounter for open fracture type IIIA, IIIB, or IIIC
S82402A	Unspecified fracture of shaft of left fibula, initial encounter for closed fracture
S82402B	Unspecified fracture of shaft of left fibula, initial encounter for open fracture type I or II
S82402C	Unspecified fracture of shaft of left fibula, initial encounter for open fracture type IIIA, IIIB, or IIIC
S82409A	Unspecified fracture of shaft of unspecified fibula, initial encounter for closed fracture
S82409B	Unspecified fracture of shaft of unspecified fibula, initial encounter for open fracture type I or II
S82409C	Unspecified fracture of shaft of unspecified fibula, initial encounter for open fracture type IIIA, IIIB, or IIIC
S82421A	Displaced transverse fracture of shaft of right fibula, initial encounter for closed fracture
S82421B	Displaced transverse fracture of shaft of right fibula, initial encounter for open fracture type I or II

S82421C	Displaced transverse fracture of shaft of right fibula, initial encounter for open fracture type IIIA, IIIB, or IIIC
S82422A	Displaced transverse fracture of shaft of left fibula, initial encounter for closed fracture
S82422B	Displaced transverse fracture of shaft of left fibula, initial encounter for open fracture type I or II
S82422C	Displaced transverse fracture of shaft of left fibula, initial encounter for open fracture type IIIA, IIIB, or IIIC
S82423A	Displaced transverse fracture of shaft of unspecified fibula, initial encounter for closed fracture
S82423B	Displaced transverse fracture of shaft of unspecified fibula, initial encounter for open fracture type I or II
S82423C	Displaced transverse fracture of shaft of unspecified fibula, initial encounter for open fracture type IIIA, IIIB, or IIIC
S82424A	Nondisplaced transverse fracture of shaft of right fibula, initial encounter for closed fracture
S82424B	Nondisplaced transverse fracture of shaft of right fibula, initial encounter for open fracture type I or II
S82424C	Nondisplaced transverse fracture of shaft of right fibula, initial encounter for open fracture type IIIA, IIIB, or IIIC
S82425A	Nondisplaced transverse fracture of shaft of left fibula, initial encounter for closed fracture
S82425B	Nondisplaced transverse fracture of shaft of left fibula, initial encounter for open fracture type I or II
S82425C	Nondisplaced transverse fracture of shaft of left fibula, initial encounter for open fracture type IIIA, IIIB, or IIIC
S82426A	Nondisplaced transverse fracture of shaft of unspecified fibula, initial encounter for closed fracture
S82426B	Nondisplaced transverse fracture of shaft of unspecified fibula, initial encounter for open fracture type I or II
S82426C	Nondisplaced transverse fracture of shaft of unspecified fibula, initial encounter for open fracture type IIIA, IIIB, or IIIC
S82431A	Displaced oblique fracture of shaft of right fibula, initial encounter for closed fracture
S82431B	Displaced oblique fracture of shaft of right fibula, initial encounter for open fracture type I or II
S82431C	Displaced oblique fracture of shaft of right fibula, initial encounter for open fracture type IIIA, IIIB, or IIIC
S82432A	Displaced oblique fracture of shaft of left fibula, initial encounter for closed fracture
S82432B	Displaced oblique fracture of shaft of left fibula, initial encounter for open fracture type I or II
S82432C	Displaced oblique fracture of shaft of left fibula, initial encounter for open fracture type IIIA, IIIB, or IIIC
S82433A	Displaced oblique fracture of shaft of unspecified fibula, initial encounter for closed fracture
S82433B	Displaced oblique fracture of shaft of unspecified fibula, initial encounter for open fracture type I or II
S82433C	Displaced oblique fracture of shaft of unspecified fibula, initial encounter for open fracture type IIIA, IIIB, or IIIC
S82434A	Nondisplaced oblique fracture of shaft of right fibula, initial encounter for closed fracture
S82434B	Nondisplaced oblique fracture of shaft of right fibula, initial encounter for open fracture type I or II
S82434C	Nondisplaced oblique fracture of shaft of right fibula, initial encounter for open fracture type IIIA, IIIB, or IIIC
S82435A	Nondisplaced oblique fracture of shaft of left fibula, initial encounter for closed fracture
S82435B	Nondisplaced oblique fracture of shaft of left fibula, initial encounter for open fracture type I or II
S82435C	Nondisplaced oblique fracture of shaft of left fibula, initial encounter for open fracture type IIIA, IIIB, or IIIC
S82436A	Nondisplaced oblique fracture of shaft of unspecified fibula, initial encounter for closed fracture
S82436B	Nondisplaced oblique fracture of shaft of unspecified fibula, initial encounter for open fracture type I or II
S82436C	Nondisplaced oblique fracture of shaft of unspecified fibula, initial encounter for open fracture type IIIA, IIIB, or IIIC

S82441A	Displaced spiral fracture of shaft of right fibula, initial encounter for closed fracture
S82441B	Displaced spiral fracture of shaft of right fibula, initial encounter for open fracture type I or II
S82441C	Displaced spiral fracture of shaft of right fibula, initial encounter for open fracture type IIIA, IIIB, or IIIC
S82442A	Displaced spiral fracture of shaft of left fibula, initial encounter for closed fracture
S82442B	Displaced spiral fracture of shaft of left fibula, initial encounter for open fracture type I or II
S82442C	Displaced spiral fracture of shaft of left fibula, initial encounter for open fracture type IIIA, IIIB, or IIIC
S82443A	Displaced spiral fracture of shaft of unspecified fibula, initial encounter for closed fracture
S82443B	Displaced spiral fracture of shaft of unspecified fibula, initial encounter for open fracture type I or II
S82443C	Displaced spiral fracture of shaft of unspecified fibula, initial encounter for open fracture type IIIA, IIIB, or IIIC
S82444A	Nondisplaced spiral fracture of shaft of right fibula, initial encounter for closed fracture
S82444B	Nondisplaced spiral fracture of shaft of right fibula, initial encounter for open fracture type I or II
S82444C	Nondisplaced spiral fracture of shaft of right fibula, initial encounter for open fracture type IIIA, IIIB, or IIIC
S82445A	Nondisplaced spiral fracture of shaft of left fibula, initial encounter for closed fracture
S82445B	Nondisplaced spiral fracture of shaft of left fibula, initial encounter for open fracture type I or II
S82445C	Nondisplaced spiral fracture of shaft of left fibula, initial encounter for open fracture type IIIA, IIIB, or IIIC
S82446A	Nondisplaced spiral fracture of shaft of unspecified fibula, initial encounter for closed fracture
S82446B	Nondisplaced spiral fracture of shaft of unspecified fibula, initial encounter for open fracture type I or II
S82446C	Nondisplaced spiral fracture of shaft of unspecified fibula, initial encounter for open fracture type IIIA, IIIB, or IIIC
S82451A	Displaced comminuted fracture of shaft of right fibula, initial encounter for closed fracture
S82451B	Displaced comminuted fracture of shaft of right fibula, initial encounter for open fracture type I or II
S82451C	Displaced comminuted fracture of shaft of right fibula, initial encounter for open fracture type IIIA, IIIB, or IIIC
S82452A	Displaced comminuted fracture of shaft of left fibula, initial encounter for closed fracture
S82452B	Displaced comminuted fracture of shaft of left fibula, initial encounter for open fracture type I or II
S82452C	Displaced comminuted fracture of shaft of left fibula, initial encounter for open fracture type IIIA, IIIB, or IIIC
S82453A	Displaced comminuted fracture of shaft of unspecified fibula, initial encounter for closed fracture
S82453B	Displaced comminuted fracture of shaft of unspecified fibula, initial encounter for open fracture type I or II
S82453C	Displaced comminuted fracture of shaft of unspecified fibula, initial encounter for open fracture type IIIA, IIIB, or IIIC
S82454A	Nondisplaced comminuted fracture of shaft of right fibula, initial encounter for closed fracture
S82454B	Nondisplaced comminuted fracture of shaft of right fibula, initial encounter for open fracture type I or II
S82454C	Nondisplaced comminuted fracture of shaft of right fibula, initial encounter for open fracture type IIIA, IIIB, or IIIC
S82455A	Nondisplaced comminuted fracture of shaft of left fibula, initial encounter for closed fracture
S82455B	Nondisplaced comminuted fracture of shaft of left fibula, initial encounter for open fracture type I or II
S82455C	Nondisplaced comminuted fracture of shaft of left fibula, initial encounter for open fracture type IIIA, IIIB, or IIIC
S82456A	Nondisplaced comminuted fracture of shaft of unspecified fibula, initial encounter for closed fracture
S82456B	Nondisplaced comminuted fracture of shaft of unspecified fibula, initial encounter for open fracture

	type I or II
S82456C	Nondisplaced comminuted fracture of shaft of unspecified fibula, initial encounter for open fracture type IIIA, IIIB, or IIIC
S82461A	Displaced segmental fracture of shaft of right fibula, initial encounter for closed fracture
S82461B	Displaced segmental fracture of shaft of right fibula, initial encounter for open fracture type I or II
S82461C	Displaced segmental fracture of shaft of right fibula, initial encounter for open fracture type IIIA, IIIB, or IIIC
S82462A	Displaced segmental fracture of shaft of left fibula, initial encounter for closed fracture
S82462B	Displaced segmental fracture of shaft of left fibula, initial encounter for open fracture type I or II
S82462C	Displaced segmental fracture of shaft of left fibula, initial encounter for open fracture type IIIA, IIIB, or IIIC
S82463A	Displaced segmental fracture of shaft of unspecified fibula, initial encounter for closed fracture
S82463B	Displaced segmental fracture of shaft of unspecified fibula, initial encounter for open fracture type I or II
S82463C	Displaced segmental fracture of shaft of unspecified fibula, initial encounter for open fracture type IIIA, IIIB, or IIIC
S82464A	Nondisplaced segmental fracture of shaft of right fibula, initial encounter for closed fracture
S82464B	Nondisplaced segmental fracture of shaft of right fibula, initial encounter for open fracture type I or II
S82464C	Nondisplaced segmental fracture of shaft of right fibula, initial encounter for open fracture type IIIA, IIIB, or IIIC
S82465A	Nondisplaced segmental fracture of shaft of left fibula, initial encounter for closed fracture
S82465B	Nondisplaced segmental fracture of shaft of left fibula, initial encounter for open fracture type I or II
S82465C	Nondisplaced segmental fracture of shaft of left fibula, initial encounter for open fracture type IIIA, IIIB, or IIIC
S82466A	Nondisplaced segmental fracture of shaft of unspecified fibula, initial encounter for closed fracture
S82466B	Nondisplaced segmental fracture of shaft of unspecified fibula, initial encounter for open fracture type I or II
S82466C	Nondisplaced segmental fracture of shaft of unspecified fibula, initial encounter for open fracture type IIIA, IIIB, or IIIC
S82491A	Other fracture of shaft of right fibula, initial encounter for closed fracture
S82491B	Other fracture of shaft of right fibula, initial encounter for open fracture type I or II
S82491C	Other fracture of shaft of right fibula, initial encounter for open fracture type IIIA, IIIB, or IIIC
S82492A	Other fracture of shaft of left fibula, initial encounter for closed fracture
S82492B	Other fracture of shaft of left fibula, initial encounter for open fracture type I or II
S82492C	Other fracture of shaft of left fibula, initial encounter for open fracture type IIIA, IIIB, or IIIC
S82499A	Other fracture of shaft of unspecified fibula, initial encounter for closed fracture
S82499B	Other fracture of shaft of unspecified fibula, initial encounter for open fracture type I or II
S82499C	Other fracture of shaft of unspecified fibula, initial encounter for open fracture type IIIA, IIIB, or IIIC
S8251XA	Displaced fracture of medial malleolus of right tibia, initial encounter for closed fracture
S8251XB	Displaced fracture of medial malleolus of right tibia, initial encounter for open fracture type I or II
S8251XC	Displaced fracture of medial malleolus of right tibia, initial encounter for open fracture type IIIA, IIIB, or IIIC
S8252XA	Displaced fracture of medial malleolus of left tibia, initial encounter for closed fracture
S8252XB	Displaced fracture of medial malleolus of left tibia, initial encounter for open fracture type I or II
S8252XC	Displaced fracture of medial malleolus of left tibia, initial encounter for open fracture type IIIA, IIIB, or IIIC
S8253XA	Displaced fracture of medial malleolus of unspecified tibia, initial encounter for closed fracture

S8253XB	Displaced fracture of medial malleolus of unspecified tibia, initial encounter for open fracture type I or II
S8253XC	Displaced fracture of medial malleolus of unspecified tibia, initial encounter for open fracture type IIIA, IIIB, or IIIC
S8254XA	Nondisplaced fracture of medial malleolus of right tibia, initial encounter for closed fracture
S8254XB	Nondisplaced fracture of medial malleolus of right tibia, initial encounter for open fracture type I or II
S8254XC	Nondisplaced fracture of medial malleolus of right tibia, initial encounter for open fracture type IIIA, IIIB, or IIIC
S8255XA	Nondisplaced fracture of medial malleolus of left tibia, initial encounter for closed fracture
S8255XB	Nondisplaced fracture of medial malleolus of left tibia, initial encounter for open fracture type I or II
S8255XC	Nondisplaced fracture of medial malleolus of left tibia, initial encounter for open fracture type IIIA, IIIB, or IIIC
S8256XA	Nondisplaced fracture of medial malleolus of unspecified tibia, initial encounter for closed fracture
S8256XB	Nondisplaced fracture of medial malleolus of unspecified tibia, initial encounter for open fracture type I or II
S8256XC	Nondisplaced fracture of medial malleolus of unspecified tibia, initial encounter for open fracture type IIIA, IIIB, or IIIC
S8261XA	Displaced fracture of lateral malleolus of right fibula, initial encounter for closed fracture
S8261XB	Displaced fracture of lateral malleolus of right fibula, initial encounter for open fracture type I or II
S8261XC	Displaced fracture of lateral malleolus of right fibula, initial encounter for open fracture type IIIA, IIIB, or IIIC
S8262XA	Displaced fracture of lateral malleolus of left fibula, initial encounter for closed fracture
S8262XB	Displaced fracture of lateral malleolus of left fibula, initial encounter for open fracture type I or II
S8262XC	Displaced fracture of lateral malleolus of left fibula, initial encounter for open fracture type IIIA, IIIB, or IIIC
S8263XA	Displaced fracture of lateral malleolus of unspecified fibula, initial encounter for closed fracture
S8263XB	Displaced fracture of lateral malleolus of unspecified fibula, initial encounter for open fracture type I or II
S8263XC	Displaced fracture of lateral malleolus of unspecified fibula, initial encounter for open fracture type IIIA, IIIB, or IIIC
S8264XA	Nondisplaced fracture of lateral malleolus of right fibula, initial encounter for closed fracture
S8264XB	Nondisplaced fracture of lateral malleolus of right fibula, initial encounter for open fracture type I or II
S8264XC	Nondisplaced fracture of lateral malleolus of right fibula, initial encounter for open fracture type IIIA, IIIB, or IIIC
S8265XA	Nondisplaced fracture of lateral malleolus of left fibula, initial encounter for closed fracture
S8265XB	Nondisplaced fracture of lateral malleolus of left fibula, initial encounter for open fracture type I or II
S8265XC	Nondisplaced fracture of lateral malleolus of left fibula, initial encounter for open fracture type IIIA, IIIB, or IIIC
S8266XA	Nondisplaced fracture of lateral malleolus of unspecified fibula, initial encounter for closed fracture
S8266XB	Nondisplaced fracture of lateral malleolus of unspecified fibula, initial encounter for open fracture type I or II
S8266XC	Nondisplaced fracture of lateral malleolus of unspecified fibula, initial encounter for open fracture type IIIA, IIIB, or IIIC
S82811A	Torus fracture of upper end of right fibula, initial encounter for closed fracture
S82812A	Torus fracture of upper end of left fibula, initial encounter for closed fracture
S82819A	Torus fracture of upper end of unspecified fibula, initial encounter for closed fracture
S82821A	Torus fracture of lower end of right fibula, initial encounter for closed fracture
S82822A	Torus fracture of lower end of left fibula, initial encounter for closed fracture

S82829A	Torus fracture of lower end of unspecified fibula, initial encounter for closed fracture
S82831A	Other fracture of upper and lower end of right fibula, initial encounter for closed fracture
S82831B	Other fracture of upper and lower end of right fibula, initial encounter for open fracture type I or II
S82831C	Other fracture of upper and lower end of right fibula, initial encounter for open fracture type IIIA, IIIB, or IIIC
S82832A	Other fracture of upper and lower end of left fibula, initial encounter for closed fracture
S82832B	Other fracture of upper and lower end of left fibula, initial encounter for open fracture type I or II
S82832C	Other fracture of upper and lower end of left fibula, initial encounter for open fracture type IIIA, IIIB, or IIIC
S82839A	Other fracture of upper and lower end of unspecified fibula, initial encounter for closed fracture
S82839B	Other fracture of upper and lower end of unspecified fibula, initial encounter for open fracture type I or II
S82839C	Other fracture of upper and lower end of unspecified fibula, initial encounter for open fracture type IIIA, IIIB, or IIIC
S82841A	Displaced bimalleolar fracture of right lower leg, initial encounter for closed fracture
S82841B	Displaced bimalleolar fracture of right lower leg, initial encounter for open fracture type I or II
S82841C	Displaced bimalleolar fracture of right lower leg, initial encounter for open fracture type IIIA, IIIB, or IIIC
S82842A	Displaced bimalleolar fracture of left lower leg, initial encounter for closed fracture
S82842B	Displaced bimalleolar fracture of left lower leg, initial encounter for open fracture type I or II
S82842C	Displaced bimalleolar fracture of left lower leg, initial encounter for open fracture type IIIA, IIIB, or IIIC
S82843A	Displaced bimalleolar fracture of unspecified lower leg, initial encounter for closed fracture
S82843B	Displaced bimalleolar fracture of unspecified lower leg, initial encounter for open fracture type I or II
S82843C	Displaced bimalleolar fracture of unspecified lower leg, initial encounter for open fracture type IIIA, IIIB, or IIIC
S82844A	Nondisplaced bimalleolar fracture of right lower leg, initial encounter for closed fracture
S82844B	Nondisplaced bimalleolar fracture of right lower leg, initial encounter for open fracture type I or II
S82844C	Nondisplaced bimalleolar fracture of right lower leg, initial encounter for open fracture type IIIA, IIIB, or IIIC
S82845A	Nondisplaced bimalleolar fracture of left lower leg, initial encounter for closed fracture
S82845B	Nondisplaced bimalleolar fracture of left lower leg, initial encounter for open fracture type I or II
S82845C	Nondisplaced bimalleolar fracture of left lower leg, initial encounter for open fracture type IIIA, IIIB, or IIIC
S82846A	Nondisplaced bimalleolar fracture of unspecified lower leg, initial encounter for closed fracture
S82846B	Nondisplaced bimalleolar fracture of unspecified lower leg, initial encounter for open fracture type I or II
S82846C	Nondisplaced bimalleolar fracture of unspecified lower leg, initial encounter for open fracture type IIIA, IIIB, or IIIC
S82851A	Displaced trimalleolar fracture of right lower leg, initial encounter for closed fracture
S82851B	Displaced trimalleolar fracture of right lower leg, initial encounter for open fracture type I or II
S82851C	Displaced trimalleolar fracture of right lower leg, initial encounter for open fracture type IIIA, IIIB, or IIIC
S82852A	Displaced trimalleolar fracture of left lower leg, initial encounter for closed fracture
S82852B	Displaced trimalleolar fracture of left lower leg, initial encounter for open fracture type I or II
S82852C	Displaced trimalleolar fracture of left lower leg, initial encounter for open fracture type IIIA, IIIB, or IIIC
S82853A	Displaced trimalleolar fracture of unspecified lower leg, initial encounter for closed fracture
S82853B	Displaced trimalleolar fracture of unspecified lower leg, initial encounter for open fracture type I or II

S82853C	Displaced trimalleolar fracture of unspecified lower leg, initial encounter for open fracture type IIIA, IIIB, or IIIC
S82854A	Nondisplaced trimalleolar fracture of right lower leg, initial encounter for closed fracture
S82854B	Nondisplaced trimalleolar fracture of right lower leg, initial encounter for open fracture type I or II
S82854C	Nondisplaced trimalleolar fracture of right lower leg, initial encounter for open fracture type IIIA, IIIB, or IIIC
S82855A	Nondisplaced trimalleolar fracture of left lower leg, initial encounter for closed fracture
S82855B	Nondisplaced trimalleolar fracture of left lower leg, initial encounter for open fracture type I or II
S82855C	Nondisplaced trimalleolar fracture of left lower leg, initial encounter for open fracture type IIIA, IIIB, or IIIC
S82856A	Nondisplaced trimalleolar fracture of unspecified lower leg, initial encounter for closed fracture
S82856B	Nondisplaced trimalleolar fracture of unspecified lower leg, initial encounter for open fracture type I or II
S82856C	Nondisplaced trimalleolar fracture of unspecified lower leg, initial encounter for open fracture type IIIA, IIIB, or IIIC
S82861A	Displaced Maisonneuve's fracture of right leg, initial encounter for closed fracture
S82861B	Displaced Maisonneuve's fracture of right leg, initial encounter for open fracture type I or II
S82861C	Displaced Maisonneuve's fracture of right leg, initial encounter for open fracture type IIIA, IIIB, or IIIC
S82862A	Displaced Maisonneuve's fracture of left leg, initial encounter for closed fracture
S82862B	Displaced Maisonneuve's fracture of left leg, initial encounter for open fracture type I or II
S82862C	Displaced Maisonneuve's fracture of left leg, initial encounter for open fracture type IIIA, IIIB, or IIIC
S82863A	Displaced Maisonneuve's fracture of unspecified leg, initial encounter for closed fracture
S82863B	Displaced Maisonneuve's fracture of unspecified leg, initial encounter for open fracture type I or II
S82863C	Displaced Maisonneuve's fracture of unspecified leg, initial encounter for open fracture type IIIA, IIIB, or IIIC
S82864A	Nondisplaced Maisonneuve's fracture of right leg, initial encounter for closed fracture
S82864B	Nondisplaced Maisonneuve's fracture of right leg, initial encounter for open fracture type I or II
S82864C	Nondisplaced Maisonneuve's fracture of right leg, initial encounter for open fracture type IIIA, IIIB, or IIIC
S82865A	Nondisplaced Maisonneuve's fracture of left leg, initial encounter for closed fracture
S82865B	Nondisplaced Maisonneuve's fracture of left leg, initial encounter for open fracture type I or II
S82865C	Nondisplaced Maisonneuve's fracture of left leg, initial encounter for open fracture type IIIA, IIIB, or IIIC
S82866A	Nondisplaced Maisonneuve's fracture of unspecified leg, initial encounter for closed fracture
S82866B	Nondisplaced Maisonneuve's fracture of unspecified leg, initial encounter for open fracture type I or II
S82866C	Nondisplaced Maisonneuve's fracture of unspecified leg, initial encounter for open fracture type IIIA, IIIB, or IIIC
S82871A	Displaced pilon fracture of right tibia, initial encounter for closed fracture
S82871B	Displaced pilon fracture of right tibia, initial encounter for open fracture type I or II
S82871C	Displaced pilon fracture of right tibia, initial encounter for open fracture type IIIA, IIIB, or IIIC
S82872A	Displaced pilon fracture of left tibia, initial encounter for closed fracture
S82872B	Displaced pilon fracture of left tibia, initial encounter for open fracture type I or II
S82872C	Displaced pilon fracture of left tibia, initial encounter for open fracture type IIIA, IIIB, or IIIC
S82873A	Displaced pilon fracture of unspecified tibia, initial encounter for closed fracture
S82873B	Displaced pilon fracture of unspecified tibia, initial encounter for open fracture type I or II
S82873C	Displaced pilon fracture of unspecified tibia, initial encounter for open fracture type IIIA, IIIB, or IIIC

S82874A	Nondisplaced pilon fracture of right tibia, initial encounter for closed fracture
S82874B	Nondisplaced pilon fracture of right tibia, initial encounter for open fracture type I or II
S82874C	Nondisplaced pilon fracture of right tibia, initial encounter for open fracture type IIIA, IIIB, or IIIC
S82875A	Nondisplaced pilon fracture of left tibia, initial encounter for closed fracture
S82875B	Nondisplaced pilon fracture of left tibia, initial encounter for open fracture type I or II
S82875C	Nondisplaced pilon fracture of left tibia, initial encounter for open fracture type IIIA, IIIB, or IIIC
S82876A	Nondisplaced pilon fracture of unspecified tibia, initial encounter for closed fracture
S82876B	Nondisplaced pilon fracture of unspecified tibia, initial encounter for open fracture type I or II
S82876C	Nondisplaced pilon fracture of unspecified tibia, initial encounter for open fracture type IIIA, IIIB, or IIIC
S82891A	Other fracture of right lower leg, initial encounter for closed fracture
S82891B	Other fracture of right lower leg, initial encounter for open fracture type I or II
S82891C	Other fracture of right lower leg, initial encounter for open fracture type IIIA, IIIB, or IIIC
S82892A	Other fracture of left lower leg, initial encounter for closed fracture
S82892B	Other fracture of left lower leg, initial encounter for open fracture type I or II
S82892C	Other fracture of left lower leg, initial encounter for open fracture type IIIA, IIIB, or IIIC
S82899A	Other fracture of unspecified lower leg, initial encounter for closed fracture
S82899B	Other fracture of unspecified lower leg, initial encounter for open fracture type I or II
S82899C	Other fracture of unspecified lower leg, initial encounter for open fracture type IIIA, IIIB, or IIIC
S8290XA	Unspecified fracture of unspecified lower leg, initial encounter for closed fracture
S8290XB	Unspecified fracture of unspecified lower leg, initial encounter for open fracture type I or II
S8290XC	Unspecified fracture of unspecified lower leg, initial encounter for open fracture type IIIA, IIIB, or IIIC
S8291XA	Unspecified fracture of right lower leg, initial encounter for closed fracture
S8291XB	Unspecified fracture of right lower leg, initial encounter for open fracture type I or II
S8291XC	Unspecified fracture of right lower leg, initial encounter for open fracture type IIIA, IIIB, or IIIC
S8292XA	Unspecified fracture of left lower leg, initial encounter for closed fracture
S8292XB	Unspecified fracture of left lower leg, initial encounter for open fracture type I or II
S8292XC	Unspecified fracture of left lower leg, initial encounter for open fracture type IIIA, IIIB, or IIIC

(e) Falls

Code	Description
R296	Repeated falls
V00111A	Fall from in-line roller-skates, initial encounter
V00121A	Fall from non-in-line roller-skates, initial encounter
V00131A	Fall from skateboard, initial encounter
V00141A	Fall from scooter (nonmotorized), initial encounter
V00151A	Fall from heelies, initial encounter
V00181A	Fall from other rolling-type pedestrian conveyance, initial encounter
V00211A	Fall from ice-skates, initial encounter
V00221A	Fall from sled, initial encounter
V00281A	Fall from other gliding-type pedestrian conveyance, initial encounter
V00311A	Fall from snowboard, initial encounter
V00321A	Fall from snow-skis, initial encounter

V00381A	Fall from other flat-bottomed pedestrian conveyance, initial encounter
V00811A	Fall from moving wheelchair (powered), initial encounter
V00821A	Fall from baby stroller, initial encounter
V00831A	Fall from motorized mobility scooter, initial encounter
V00841A	Fall from standing electric scooter, initial encounter
V00891A	Fall from other pedestrian conveyance, initial encounter
V80010A	Animal-rider injured by fall from or being thrown from horse in noncollision accident, initial encounter
V80018A	Animal-rider injured by fall from or being thrown from other animal in noncollision accident, initial encounter
V8002XA	Occupant of animal-drawn vehicle injured by fall from or being thrown from animal-drawn vehicle in noncollision accident, initial encounter
V815XXA	Occupant of railway train or railway vehicle injured by fall in railway train or railway vehicle, initial encounter
V816XXA	Occupant of railway train or railway vehicle injured by fall from railway train or railway vehicle, initial encounter
V825XXA	Occupant of streetcar injured by fall in streetcar, initial encounter
V826XXA	Occupant of streetcar injured by fall from streetcar, initial encounter
V9120XA	Fall due to collision between merchant ship and other watercraft or other object, initial encounter
V9121XA	Fall due to collision between passenger ship and other watercraft or other object, initial encounter
V9122XA	Fall due to collision between fishing boat and other watercraft or other object, initial encounter
V9123XA	Fall due to collision between other powered watercraft and other watercraft or other object, initial encounter
V9124XA	Fall due to collision between sailboat and other watercraft or other object, initial encounter
V9125XA	Fall due to collision between canoe or kayak and other watercraft or other object, initial encounter
V9126XA	Fall due to collision between (nonpowered) inflatable craft and other watercraft or other object, initial encounter
V9129XA	Fall due to collision between unspecified watercraft and other watercraft or other object, initial encounter
V9200XA	Drowning and submersion due to fall off merchant ship, initial encounter
V9201XA	Drowning and submersion due to fall off passenger ship, initial encounter
V9202XA	Drowning and submersion due to fall off fishing boat, initial encounter
V9203XA	Drowning and submersion due to fall off other powered watercraft, initial encounter
V9204XA	Drowning and submersion due to fall off sailboat, initial encounter
V9205XA	Drowning and submersion due to fall off canoe or kayak, initial encounter
V9206XA	Drowning and submersion due to fall off (nonpowered) inflatable craft, initial encounter
V9207XA	Drowning and submersion due to fall off water-skis, initial encounter
V9208XA	Drowning and submersion due to fall off other unpowered watercraft, initial encounter

V9209XA	Drowning and submersion due to fall off unspecified watercraft, initial encounter
V9330XA	Fall on board merchant ship, initial encounter
V9331XA	Fall on board passenger ship, initial encounter
V9332XA	Fall on board fishing boat, initial encounter
V9333XA	Fall on board other powered watercraft, initial encounter
V9334XA	Fall on board sailboat, initial encounter
V9335XA	Fall on board canoe or kayak, initial encounter
V9336XA	Fall on board (nonpowered) inflatable craft, initial encounter
V9338XA	Fall on board other unpowered watercraft, initial encounter
V9339XA	Fall on board unspecified watercraft, initial encounter
V940XXA	Hitting object or bottom of body of water due to fall from watercraft, initial encounter
W000XXA	Fall on same level due to ice and snow, initial encounter
W001XXA	Fall from stairs and steps due to ice and snow, initial encounter
W002XXA	Other fall from one level to another due to ice and snow, initial encounter
W009XXA	Unspecified fall due to ice and snow, initial encounter
W010XXA	Fall on same level from slipping, tripping and stumbling without subsequent striking against object, initial encounter
W0110XA	Fall on same level from slipping, tripping and stumbling with subsequent striking against unspecified object, initial encounter
W01110A	Fall on same level from slipping, tripping and stumbling with subsequent striking against sharp glass, initial encounter
W01111A	Fall on same level from slipping, tripping and stumbling with subsequent striking against power tool or machine, initial encounter
W01118A	Fall on same level from slipping, tripping and stumbling with subsequent striking against other sharp object, initial encounter
W01119A	Fall on same level from slipping, tripping and stumbling with subsequent striking against unspecified sharp object, initial encounter
W01190A	Fall on same level from slipping, tripping and stumbling with subsequent striking against furniture, initial encounter
W01198A	Fall on same level from slipping, tripping and stumbling with subsequent striking against other object, initial encounter
W03XXXA	Other fall on same level due to collision with another person, initial encounter
W04XXXA	Fall while being carried or supported by other persons, initial encounter
W050XXA	Fall from non-moving wheelchair, initial encounter
W051XXA	Fall from non-moving nonmotorized scooter, initial encounter
W052XXA	Fall from non-moving motorized mobility scooter, initial encounter
W06XXXA	Fall from bed, initial encounter
W07XXXA	Fall from chair, initial encounter
W08XXXA	Fall from other furniture, initial encounter
W090XXA	Fall on or from playground slide, initial encounter
W091XXA	Fall from playground swing, initial encounter
W092XXA	Fall on or from jungle gym, initial encounter
W098XXA	Fall on or from other playground equipment, initial encounter
W100XXA	Fall (on)(from) escalator, initial encounter

W101XXA	Fall (on)(from) sidewalk curb, initial encounter
W102XXA	Fall (on)(from) incline, initial encounter
W108XXA	Fall (on) (from) other stairs and steps, initial encounter
W109XXA	Fall (on) (from) unspecified stairs and steps, initial encounter
W11XXXA	Fall on and from ladder, initial encounter
W12XXXA	Fall on and from scaffolding, initial encounter
W130XXA	Fall from, out of or through balcony, initial encounter
W131XXA	Fall from, out of or through bridge, initial encounter
W132XXA	Fall from, out of or through roof, initial encounter
W133XXA	Fall through floor, initial encounter
W134XXA	Fall from, out of or through window, initial encounter
W138XXA	Fall from, out of or through other building or structure, initial encounter
W139XXA	Fall from, out of or through building, not otherwise specified, initial encounter
W14XXXA	Fall from tree, initial encounter
W15XXXA	Fall from cliff, initial encounter
W16011A	Fall into swimming pool striking water surface causing drowning and submersion, initial encounter
W16012A	Fall into swimming pool striking water surface causing other injury, initial encounter
W16021A	Fall into swimming pool striking bottom causing drowning and submersion, initial encounter
W16022A	Fall into swimming pool striking bottom causing other injury, initial encounter
W16031A	Fall into swimming pool striking wall causing drowning and submersion, initial encounter
W16032A	Fall into swimming pool striking wall causing other injury, initial encounter
W16111A	Fall into natural body of water striking water surface causing drowning and submersion, initial encounter
W16112A	Fall into natural body of water striking water surface causing other injury, initial encounter
W16121A	Fall into natural body of water striking bottom causing drowning and submersion, initial encounter
W16122A	Fall into natural body of water striking bottom causing other injury, initial encounter
W16131A	Fall into natural body of water striking side causing drowning and submersion, initial encounter
W16132A	Fall into natural body of water striking side causing other injury, initial encounter
W16211A	Fall in (into) filled bathtub causing drowning and submersion, initial encounter
W16212A	Fall in (into) filled bathtub causing other injury, initial encounter
W16221A	Fall in (into) bucket of water causing drowning and submersion, initial encounter
W16222A	Fall in (into) bucket of water causing other injury, initial encounter
W16311A	Fall into other water striking water surface causing drowning and submersion, initial encounter
W16312A	Fall into other water striking water surface causing other injury, initial encounter
W16321A	Fall into other water striking bottom causing drowning and submersion, initial encounter
W16322A	Fall into other water striking bottom causing other injury, initial encounter

W16331A	Fall into other water striking wall causing drowning and submersion, initial encounter
W16332A	Fall into other water striking wall causing other injury, initial encounter
W1641XA	Fall into unspecified water causing drowning and submersion, initial encounter
W1642XA	Fall into unspecified water causing other injury, initial encounter
W170XXA	Fall into well, initial encounter
W171XXA	Fall into storm drain or manhole, initial encounter
W172XXA	Fall into hole, initial encounter
W173XXA	Fall into empty swimming pool, initial encounter
W174XXA	Fall from dock, initial encounter
W1781XA	Fall down embankment (hill), initial encounter
W1782XA	Fall from (out of) grocery cart, initial encounter
W1789XA	Other fall from one level to another, initial encounter
W1800XA	Striking against unspecified object with subsequent fall, initial encounter
W1801XA	Striking against sports equipment with subsequent fall, initial encounter
W1802XA	Striking against glass with subsequent fall, initial encounter
W1809XA	Striking against other object with subsequent fall, initial encounter
W1811XA	Fall from or off toilet without subsequent striking against object, initial encounter
W1812XA	Fall from or off toilet with subsequent striking against object, initial encounter
W182XXA	Fall in (into) shower or empty bathtub, initial encounter
W1830XA	Fall on same level, unspecified, initial encounter
W1831XA	Fall on same level due to stepping on an object, initial encounter
W1839XA	Other fall on same level, initial encounter
W19XXXA	Unspecified fall, initial encounter
X003XXA	Fall from burning building or structure in uncontrolled fire, initial encounter
X013XXA	Fall due to uncontrolled fire, not in building or structure, initial encounter
X023XXA	Fall from burning building or structure in controlled fire, initial encounter
X033XXA	Fall due to controlled fire, not in building or structure, initial encounter
Y211XXA	Drowning and submersion after fall into bathtub, undetermined intent, initial encounter
Y213XXA	Drowning and submersion after fall into swimming pool, undetermined intent, initial encounter

Appendix B

ICD-10 and ICD-9 codes for diagnosis groups: (a) pruritus, (b) neuropathic pain

(a) Pruritus

ICD-10 code	ICD-10 description
**L280**	Lichen simplex chronicus
**L281**	Prurigo nodularis
**L282**	Other prurigo
**L29**	Pruritus
**L290**	Pruritus ani
**L291**	Pruritus scroti
**L292**	Pruritus vulvae
**L293**	Anogenital pruritus, unspecified
**L298**	Other pruritus
**L299**	Pruritus, unspecified

ICD-9 code	ICD-9 description
**698**	Pruritus and related conditions
**6980**	Pruritus ani
**6981**	Pruritus of genital organs
**6982**	Prurigo
**6983**	Lichenification and lichen simplex chronicus
**6984**	Dermatitis factitia [artefacta]
**6988**	Other specified pruritic conditions
**6989**	Unspecified pruritic disorder

(b) Neuropathic pain

ICD-10 code	ICD-10 description
**L981**	Factitial dermatitis
**A5215**	Late syphilitic neuropathy
**E0840**	Diabetes mellitus due to underlying condition with diabetic neuropathy, unspecified
**E0841**	Diabetes mellitus due to underlying condition with diabetic mononeuropathy
**E0842**	Diabetes mellitus due to underlying condition with diabetic polyneuropathy
**E0849**	Diabetes mellitus due to underlying condition with other diabetic neurological complication
**E0940**	Drug or chemical induced diabetes mellitus with neurological complications with diabetic neuropathy, unspecified
**E0941**	Drug or chemical induced diabetes mellitus with neurological complications with diabetic mononeuropathy
**E0942**	Drug or chemical induced diabetes mellitus with neurological complications with diabetic polyneuropathy
**E0949**	Drug or chemical induced diabetes mellitus with neurological complications

	with other diabetic neurological complication
**E1040**	Type 1 diabetes mellitus with diabetic neuropathy, unspecified
**E1041**	Type 1 diabetes mellitus with diabetic mononeuropathy
**E1042**	Type 1 diabetes mellitus with diabetic polyneuropathy
**E1049**	Type 1 diabetes mellitus with other diabetic neurological complication
**E1140**	Type 2 diabetes mellitus with diabetic neuropathy, unspecified
**E1141**	Type 2 diabetes mellitus with diabetic mononeuropathy
**E1142**	Type 2 diabetes mellitus with diabetic polyneuropathy
**E1149**	Type 2 diabetes mellitus with other diabetic neurological complication
**E1340**	Other specified diabetes mellitus with diabetic neuropathy, unspecified
**E1341**	Other specified diabetes mellitus with diabetic mononeuropathy
**E1342**	Other specified diabetes mellitus with diabetic polyneuropathy
**E1349**	Other specified diabetes mellitus with other diabetic neurological complication
**G3289**	Other specified degenerative disorders of nervous system in diseases classified elsewhere
**G500**	Trigeminal neuralgia
**G501**	Atypical facial pain
**G508**	Other disorders of trigeminal nerve
**G509**	Disorder of trigeminal nerve, unspecified
**G5640**	Causalgia of unspecified upper limb
**G5641**	Causalgia of right upper limb
**G5642**	Causalgia of left upper limb
**G5690**	Unspecified mononeuropathy of unspecified upper limb
**G5691**	Unspecified mononeuropathy of right upper limb
**G5692**	Unspecified mononeuropathy of left upper limb
**G5770**	Causalgia of unspecified lower limb
**G5771**	Causalgia of right lower limb
**G5772**	Causalgia of left lower limb
**G5790**	Unspecified mononeuropathy of unspecified lower limb
**G5791**	Unspecified mononeuropathy of right lower limb
**G5792**	Unspecified mononeuropathy of left lower limb
**G588**	Other specified mononeuropathies
**G589**	Mononeuropathy, unspecified
**G59**	Mononeuropathy in diseases classified elsewhere
**G600**	Hereditary motor and sensory neuropathy
**G602**	Neuropathy in association with hereditary ataxia
**G609**	Hereditary and idiopathic neuropathy, unspecified
**G63**	Polyneuropathy in diseases classified elsewhere
**G650**	Sequelae of Guillain-Barre syndrome
**G651**	Sequelae of other inflammatory polyneuropathy
**G652**	Sequelae of toxic polyneuropathy
**G890**	Central pain syndrome

**G902**	Horner's syndrome
**G9050**	Complex regional pain syndrome I, unspecified
**G90511**	Complex regional pain syndrome I of right upper limb
**G90512**	Complex regional pain syndrome I of left upper limb
**G90513**	Complex regional pain syndrome I of upper limb, bilateral
**G90519**	Complex regional pain syndrome I of unspecified upper limb
**G90521**	Complex regional pain syndrome I of right lower limb
**G90522**	Complex regional pain syndrome I of left lower limb
**G90523**	Complex regional pain syndrome I of lower limb, bilateral
**G90529**	Complex regional pain syndrome I of unspecified lower limb
**G9059**	Complex regional pain syndrome I of other specified site
**G908**	Other disorders of autonomic nervous system
**G909**	Disorder of the autonomic nervous system, unspecified
**G968**	Other specified disorders of central nervous system
**G969**	Disorder of central nervous system, unspecified
**G980**	Neurogenic arthritis, not elsewhere classified
**G988**	Other disorders of nervous system
**G990**	Autonomic neuropathy in diseases classified elsewhere
**G998**	Other specified disorders of nervous system in diseases classified elsewhere
**M3483**	Systemic sclerosis with polyneuropathy
**M5114**	Intervertebral disc disorders with radiculopathy, thoracic region
**M5115**	Intervertebral disc disorders with radiculopathy, thoracolumbar region
**M5116**	Intervertebral disc disorders with radiculopathy, lumbar region
**M5117**	Intervertebral disc disorders with radiculopathy, lumbosacral region
**M5381**	Other specified dorsopathies, occipito-atlanto-axial region
**M5382**	Other specified dorsopathies, cervical region
**M5383**	Other specified dorsopathies, cervicothoracic region
**M5410**	Radiculopathy, site unspecified
**M5414**	Radiculopathy, thoracic region
**M5415**	Radiculopathy, thoracolumbar region
**M5416**	Radiculopathy, lumbar region
**M5417**	Radiculopathy, lumbosacral region
**M5418**	Radiculopathy, sacral and sacrococcygeal region
**M5430**	Sciatica, unspecified side
**M5431**	Sciatica, right side
**M5432**	Sciatica, left side
**M5440**	Lumbago with sciatica, unspecified side
**M5441**	Lumbago with sciatica, right side
**M5442**	Lumbago with sciatica, left side

ICD-9 code	ICD-9 description

**3371**	Aut neuropthy in oth dis
**3379**	Autonomic nerve dis NEC
**3380**	Central pain syndrome
**3499**	Cns disorder NOS
**3501**	Trigeminal neuralgia
**3502**	Atypical face pain
**3508**	Trigeminal nerve dis NEC
**3509**	Trigeminal nerve dis NOS
**3544**	Causalgia upper limb
**3549**	Mononeuritis arm NOS
**3558**	Mononeuritis leg NOS
**3559**	Mononeuritis NOS
**3560**	Hered periph neuropathy
**3569**	Idio periph neurpthy NOS
**3572**	Neuropathy in diabetes
**3574**	Neuropathy in other dis
**7238**	Cervical syndrome NEC
**7243**	Sciatica
**7244**	Lumbosacral neuritis NOS
**7292**	Neuralgia/neuritis NOS
**33720**	Unsp rflx sympth dystrph
**33721**	Rflx sym dystrph up limb
**33722**	Rflx sym dystrph lwr lmb
**33729**	Rflx sym dystrph oth st
**34989**	Cns disorder NEC
**35571**	Causalgia lower limb
